# Cross-genus analysis reveals architecturally programmed sgRNA synthesis patterns in coronaviruses

**DOI:** 10.1038/s44320-026-00239-0

**Published:** 2026-07-30

**Authors:** Zi Wen, Lei Chen, Dehua Luo, Ju Sun, Liangrong Guo, Yingxiang Deng, Zhiyuan Huang, Yuxiang Wang, Ke Pan, Fan Wang, Shaobo Xiao, Li Li, Dengguo Wei

**Affiliations:** 1 National Key Laboratory of Agricultural Microbiology, College of Veterinary Medicine, Huazhong Agricultural University, Wuhan, Hubei 430070 China; 2 Hubei Hongshan Laboratory, Interdisciplinary Sciences Institute, Huazhong Agricultural University, Wuhan, Hubei 430070 China; 3 College of Informatics, Huazhong Agricultural University, Wuhan, Hubei 430070 China; 4 Yazhou Bay National Laboratory, Sanya, Hainan 572024 China; 5 National Reference Laboratory of Veterinary Drug Residues (HZAU) and National Safety Laboratory of Veterinary Drug (HZAU), MOA Key Laboratory for Detection of Veterinary Drug Residues, MOA Laboratory for Risk Assessment of Quality and Safety of Livestock and Poultry Products, Wuhan, Hubei 430070 China; 6 National Key Laboratory of Green Pesticide, International Joint Research Center for Intelligent Biosensor Technology and Health, Central China Normal University, Wuhan, Hubei 430079 China; 7 Frontiers Science Center for Animal Breeding and Sustainable Production, Wuhan, Hubei 430070 China; 8 Shenzhen Institute of Nutrition and Health, Huazhong Agricultural University, Shenzhen, 518000 China; 9 Shenzhen Branch, Guangdong Laboratory for Lingnan Modern Agriculture, Genome Analysis Laboratory of the Ministry of Agriculture, Agricultural Genomics Institute at Shenzhen, Chinese Academy of Agricultural Sciences, Shenzhen, 518000 China

**Keywords:** Chromatin, Transcription & Genomics, Microbiology, Virology & Host Pathogen Interaction

## Abstract

Coronaviruses employ discontinuous transcription to produce canonical subgenomic RNAs (sgRNAs) essential for gene expression. Although TRS-dependent template switching mechanism has been proposed, its structural basis remains poorly defined, and the functional significance of abundant non-canonical sgRNAs persists as a critical gap since the discovery of discontinuous RNA synthesis. Here, we help bridge this gap through the first cross-genus integrated analysis of coronavirus transcriptomes and RNA interactomes. We show that canonical sgRNA formation is associated with same-direction RNA-RNA interactions. In contrast, non-canonical sgRNAs form through distinct architectural mechanisms: short-range junctions mediated by stem-loop structures overlapping genomic deletion hotspots, and conserved long-range ORF1a-N interactions generating sgRNAs encoding immune-modulatory ORFs — a function not previously attributed to non-canonical transcription. These findings suggest architecturally programmed discontinuous RNA synthesis and highlight a potential link between non-canonical sgRNAs, genomic plasticity, and immune modulation, which may have implications for coronavirus adaptation.

## Synopsis

A cross‑genus integration of transcriptomes and RNA interactomes uncovers three distinct RNA architectural programs that govern coronavirus subgenomic RNA synthesis and link non‑canonical transcription to genomic deletions and immune modulation.

- A cross-genus resource of 234 transcriptomes and 12 RNA-RNA interactomes (CODE) was assembled, revealing a conserved U-shaped distance-dependent interaction profile across four coronavirus genera.
- Non-canonical junction abundance was observed to increase with serial passage and to vary with infection time and viral genetic background, revealing complex context-dependent transcription dynamics.
- Canonical sgRNA synthesis was associated with same-direction RNA-RNA interactions, whereas short-range non-canonical junctions were mediated by stem-loop structures overlapping genomic deletion hotspots.
- Long-range ORF1a-N junctions generated sgRNAs encoding immune-modulatory ORFs within the 3′ untranslated region, a mechanism distinct from canonical transcription.

## Abstract (web summary)

A cross‑genus integration of transcriptomes and RNA interactomes uncovers three distinct RNA architectural programs that govern coronavirus subgenomic RNA synthesis and link non‑canonical transcription to genomic deletions and immune modulation.

## Graphical abstract

**Figure:**
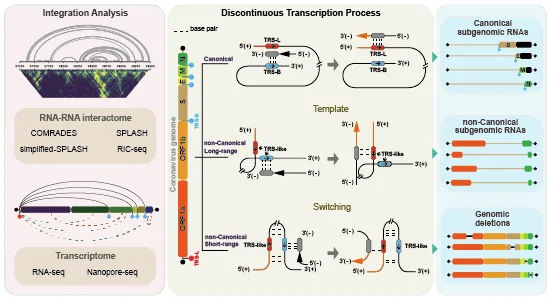

**Figure:**
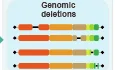

## Introduction

Coronaviruses utilize discontinuous transcription to produce subgenomic RNAs (sgRNAs), a conserved mechanism critical for regulating viral gene expression and adaptation (Sola et al, 2011; Kim et al, 2020; Chang et al, 2021; Gribble et al, 2021; Wells et al, 2023). While canonical sgRNA production relies on template switching from the transcription regulatory sequence in the body (TRS-B) to that in the leader (TRS-L) during RNA synthesis (Sola et al, 2011, 2015; Mori et al, 2023), this process requires genome-wide spatial coordination—a feature poorly addressed in existing models. For instance, the influential Sola model (Sola et al, 2015) posits TRS-L/TRS-B proximity facilitates RdRp switching, but fails to elucidate how they interact structurally. Diverging from this canonical framework, non‑canonical junctions are defined as discontinuous transcription events that do not involve the TRS‑L. During the replicative cycle of SARS-CoV-2 (Kim et al, 2020; Chang et al, 2021; Mori et al, 2023) and other coronaviruses (Sashittal et al, 2021; Lyu et al, 2022), such TRS‑independent junctions have been found to generate non‑canonical sgRNAs. These non‑canonical sgRNAs could be translated into polypeptides (Wu et al, 2022), or be defective viral genomes (Wells et al, 2023). However, their structural determinants and evolutionary significance remain controversial. This demands direct interrogation of the RNA–RNA interactome driving discontinuous transcription.

Advances in transcriptome and RNA–RNA interactome sequencing techniques have offered new tools to study the discontinuous transcription mechanism. RNA-seq data provides precise short sequencing reads, facilitating the identification of junction sites within sgRNAs (Mori et al, 2023). Nanopore sequencing allows the comprehensive identification of sgRNAs and enables accurate quantification of their transcription levels (Chang et al, 2021). Genome-wide RNA–RNA interactions within the coronavirus have been inferred by methodologies such as COMRADES (Ziv et al, 2020), SPLASH (Yang et al, 2021a), simplified-SPLASH (Zhang et al, 2021), and RIC-seq (Cao et al, 2021a), which rely on capturing proximity-ligated chimeric reads. The abundance of these chimeric reads indicates the RNA–RNA interaction strength (Cao et al, 2021a), with stronger interactions suggesting closer spatial proximity. This significant interaction strength can thus serve as predictive constraints for base pairing within RNA architectures, which are intricately linked to transcription and replication (Ziv et al, 2020; Yang et al, 2021a; Zhang et al, 2021; Cao et al, 2021a). Zhang et al. identified distant interactions between TRS-L and TRS-B in the SARS-CoV-2 genome through simplified-SPLASH, which suggests a potential role of genome architecture in mediating canonical sgRNA formation (Zhang et al, 2021). However, the specific RNA architectures governing canonical sgRNA formation lack systematic characterization, while the structural basis and functional roles of non-canonical sgRNAs remain elusive—collectively highlighting the need for comprehensive investigation.

The Coronaviridae family comprises four genera: α-, β-, γ-, and δ-coronaviruses. Current RNA–RNA interactome studies predominantly focus on β-coronaviruses, such as SARS-CoV-2 and MERS-CoV. To address the limited data coverage for other coronaviruses, we performed RNA-seq and RIC-seq experiments on the α-coronavirus PEDV (Porcine Epidemic Diarrhea Virus) and the δ-coronavirus PDCoV (Porcine deltacoronavirus). By integrating public datasets with our results, we constructed a cross-genus comprehensive resource of 234 transcriptomes and 12 RNA–RNA interactomes, hosted in the publicly accessible Coronavirus Omics Data Explorer (CODE: https://webofgroup.cn/dgwei/hngene/#/index). Through this integrated analysis, we show that the ratio of canonical to non-canonical junctions is influenced by context-dependent factors. Comparative analysis of the RNA architectures associated with the production of canonical versus non-canonical sgRNAs revealed distinct underlying mechanisms governing their synthesis. Our analysis offers insights into the potential involvement of distinct non-canonical sgRNA types in viral evolution and host adaptation. By mapping genus-conserved RNA–RNA interactions, this study establishes a framework for investigating the structural basis of discontinuous transcription in coronaviruses.

## Results

### Pan-coronavirus transcriptome and RNA–RNA interactome atlas

**Figure 1:**
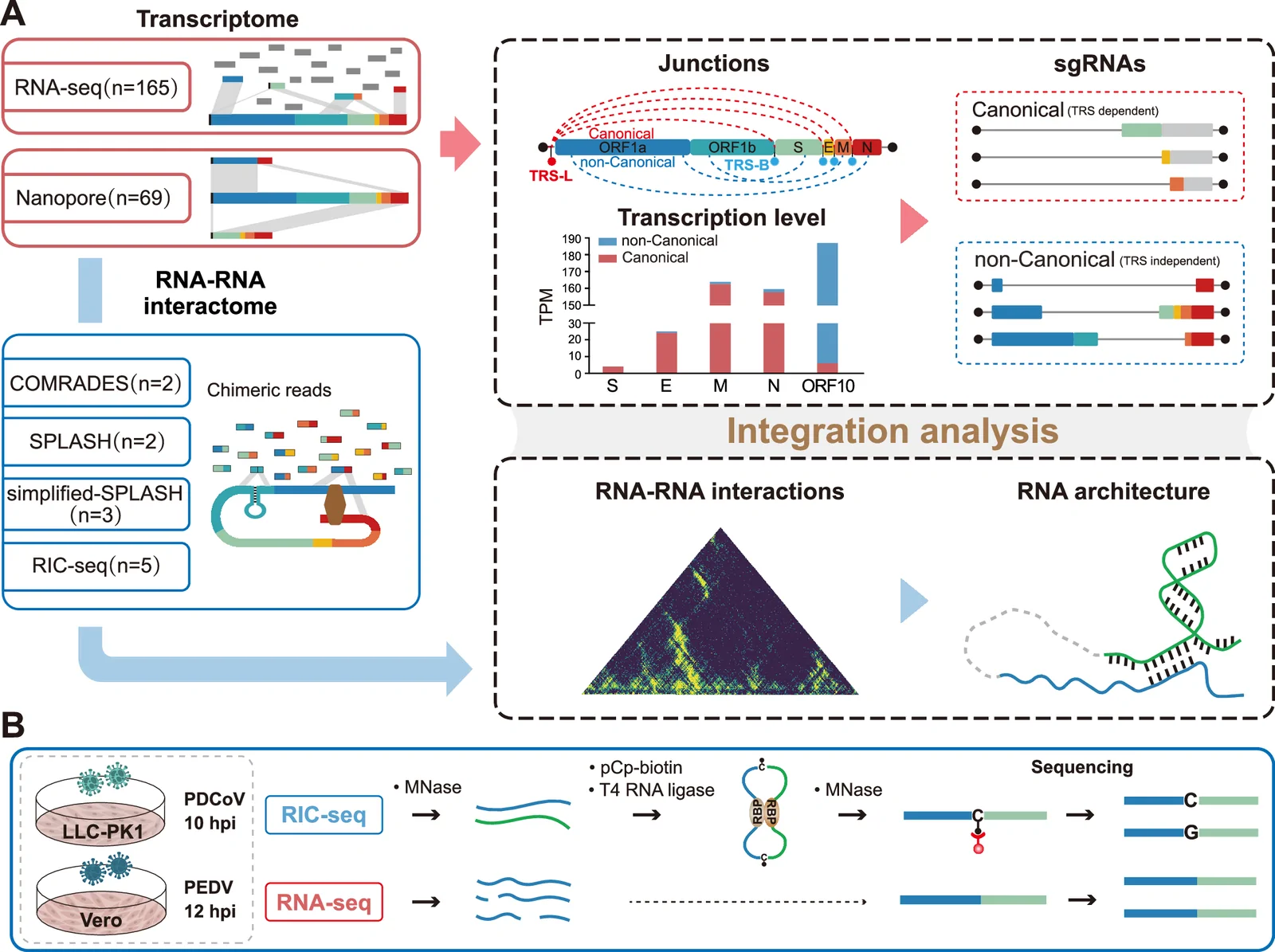
Integration analysis for RNA–RNA interactome and transcriptome profiles. (**A**) Integrative analysis framework. Schematic of RNA–RNA interactome and transcriptome data integration to link RNA architectures with subgenomic RNA (sgRNA) transcription in coronaviruses. (**B**) Experimental workflow for RIC‑seq and RNA‑seq. Vero E6 cells (1 × 10^7^) were infected with porcine epidemic diarrhea virus (PEDV) at an MOI of 0.1 for 12 h (4 biological replicates). LLC‑PK1 cells (1 × 10^7^) were infected with porcine deltacoronavirus (PDCoV) at an MOI of 0.1 for 10 h (3 biological replicates). Infected cells were either crosslinked for proximity ligation (RIC‑seq) or directly harvested for total RNA extraction (RNA‑seq). The resulting data were used to generate genome‑wide RNA–RNA interaction maps (RIC‑seq) and to identify canonical and non‑canonical sgRNA junctions (RNA‑seq).

To investigate the impact of genome architecture on transcriptional regulation, we integrated 12 RNA–RNA interactome maps with 234 transcriptome profiles (165 RNA-seq and 69 nanopore long-read samples) spanning four coronavirus genera (Fig. 1A). After filtering based on viral genome mapping efficiency (>0.5%) and sufficient discontinuous events (>5000 junctions per sample), 103 of the 165 RNA-seq samples were retained for subsequent analysis (Dataset EV1). The dataset generated in this study was processed and deposited in CODE (available at: https://webofgroup.cn/dgwei/hngene/#/index), a publicly accessible repository we developed. RNA-seq and nanopore sequencing were used to quantify sgRNA species across diverse host cell types, delineating dynamic transcriptomic changes during coronavirus infection. Additionally, full-length sgRNA reconstruction from nanopore reads enabled precise quantification of individual viral gene expression levels (Fig. 1A). By analyzing genome-wide RNA–RNA interactions captured through proximity-ligated chimeric reads, we inferred both global and local architectures of coronavirus genomes (Fig. 1A).

### Shared genome folding features across coronaviruses

While existing RNA interactome studies have focused on β-coronaviruses (e.g., SARS-CoV-2 and MERS-CoV), the structural principles governing transcription in other genera of coronaviruses remain unexplored. To address this gap, we performed an integrated analysis of RIC-seq and RNA-seq during PEDV (α-coronavirus) and PDCoV (δ-coronavirus) infections (Fig. 1B), capturing both RNA–RNA interactions and transcriptional outputs. Quality control analyses confirmed high reproducibility between biological replicates and recapitulation of known RNA structures, including the 5′ and 3′ UTR stem‑loops (Appendix Figs. S1–S3).

Quantitative analysis revealed a consistent U-shaped RNA–RNA interaction frequency distribution across all coronaviruses and experimental methodologies, as shown by both absolute and genome-length-normalized distances (Appendix Fig. S4A,B). Within 5 kb genomic distances, interactions followed a power-law decay (slope close to −1), reaching minimal frequencies between 5 and 20 kb before gradually increasing at distances >20 kb. This consistent U-shaped architecture, with preferential enrichment of short- and long-range RNA interactions, suggests that coronaviruses employ a conserved hierarchical genome folding strategy to potentially coordinate transcription-replication processes during infection.

### Condition-dependent shifts between canonical and non-canonical transcription junctions in coronaviruses

**Figure 2:**
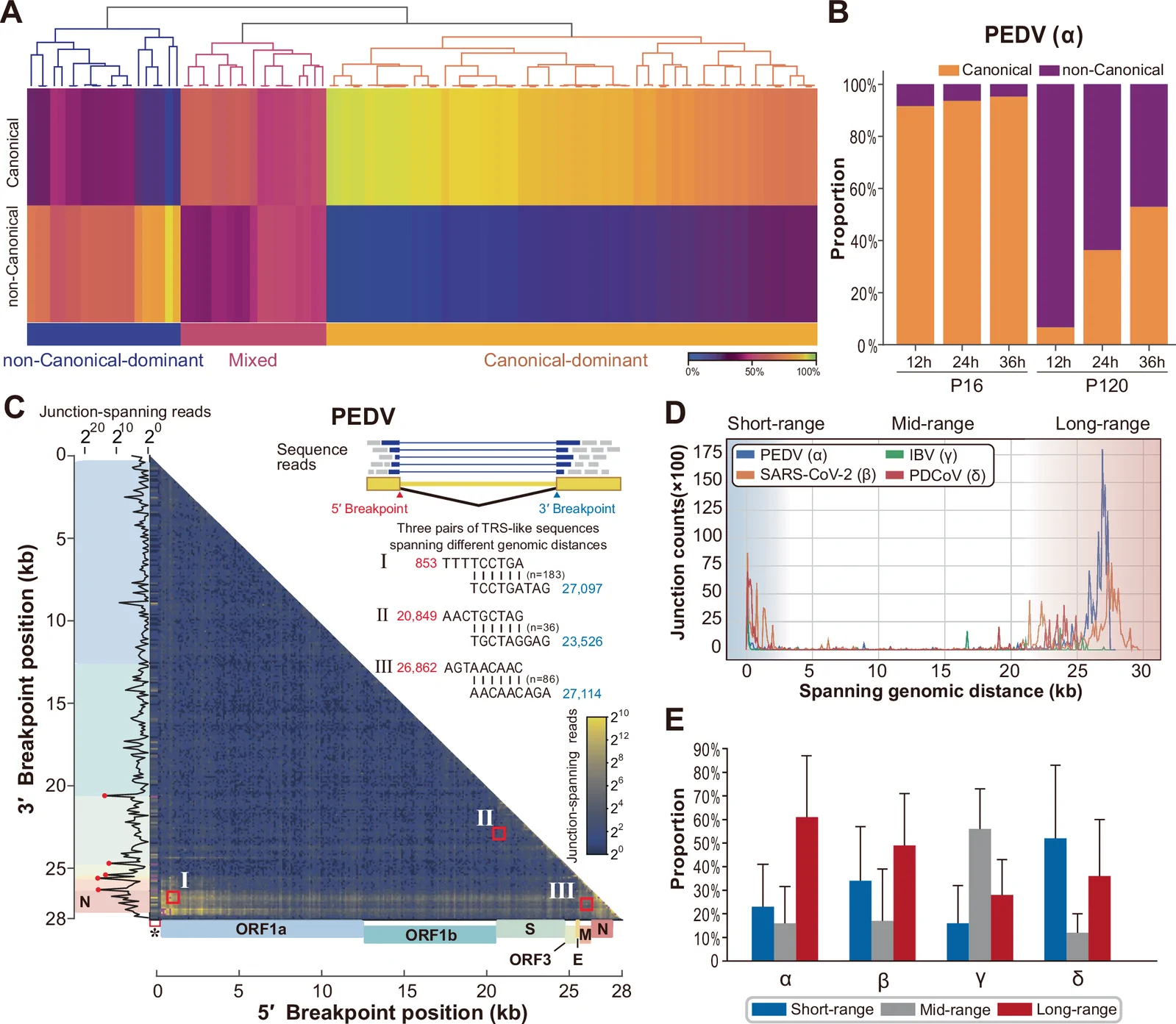
Characterization of non-canonical junctions. (**A**) Clustering of canonical and non-canonical transcription events. Heatmap showing junction type proportions across 103 RNA-seq samples that passed filtering (viral mapping efficiency >0.5% and >5000 junctions per sample). Samples are classified into canonical-dominant, mixed, and non-canonical-dominant groups by hierarchical clustering. (**B**) Proportion of canonical and non‑canonical junctions in RNA‑seq samples from porcine small intestinal 2‑D enteroid cells infected with PEDV (NCBI BioProject PRJNA801927). Cells were infected with low‑passage (P16) or high‑passage (P120) PEDV strain at an MOI of 1.0 and harvested at 12, 24, and 36 hpi. Three independent biological replicates were analyzed per condition. Each bar shows the mean proportion across the three replicates. (**C**) Genome-wide distribution of junction sites in PEDV. The heatmap shows junction density across the PEDV genome, with coordinates and gene annotations on the axes. All junctions (both canonical and non-canonical) are included. The starred region at the 5′ end of the genome indicates the leader region containing TRS‑L. The starred region at the 5′ end indicates the leader TRS‑L. The left line plot shows 3′ breakpoints of chimeric reads; red dots mark local peaks at TRS‑B sites. The top‑right panel highlights three TRS‑like sequence pairs at short, mid, and long genomic distances. (**D**) Visualization of the distribution of non-canonical junctions at different spanning genomic distances from four genera of coronaviruses. Non-canonical junctions predominantly occur in short-range and long-range genomic distances. (**E**) Average proportion of non‑canonical junctions with short‑range, mid‑range, and long‑range genomic distances across four coronavirus genera. Bars represent the mean proportion across all samples within each genus; error bars indicate standard deviation. The number of samples for α‑, β‑, γ‑, and δ‑coronaviruses are 21, 73, 5, and 4, respectively. Source data are available online for this figure.

We systematically classified canonical (TRS-dependent) and non-canonical (TRS-independent) discontinuous transcription events across these collected RNA samples. This dataset spans 14 coronaviruses under diverse experimental conditions, including passages, hpi, cell types, multiplicity of infection (MOI), and drug treatments. Unsupervised hierarchical clustering based on junction type proportions partitioned samples into three distinct clusters (Fig. 2A): a canonical-dominant group (63.1%; *n* = 65), a mixed group (19.4%; *n* = 20), and a non-canonical-dominant group (17.5%; *n* = 18). Canonical junctions were frequently observed in SARS-CoV-2 (23/31 samples) and MERS-CoV (16/20 samples), collectively contributing to an average canonical junction proportion of 69.07% across all samples (Appendix Fig. S4C).

In a public PEDV dataset (Mao et al, 2022; Data ref: Mao et al, 2022), a temporal decline in non-canonical junction proportions was observed with increasing hpi in both low- and high-passage samples (Fig. 2B). To systematically evaluate the dynamics of non-canonical transcription, the analysis was extended to all available RNA-seq datasets with differences in time courses, serial passages, or other experimental perturbations (Appendix Figs. S5 and S6). The effect of infection time varied across viruses (Appendix Fig. S5): for MERS-CoV and MHV, the proportion of canonical junctions decreased over time; for SARS-CoV and IBV, it increased; and for SARS-CoV-2, the proportion of canonical junctions generally decreased over time, except for transient increases in Vero E6 cells (24 to 48 hpi) and A549 cells (18 to 24 hpi). The accumulation of non-canonical junctions with serial passage is a general phenomenon, observed in five out of six independent passage datasets (Appendix Fig. S6A). Furthermore, certain perturbations markedly altered the ratio of canonical to non-canonical junctions (Appendix Fig. S6B–E), such as luteolin treatment or a PEDV mutant strain, whereas others, including treatment with the nucleoside analog NHC (EIDD-1931), IRE1 knockout, or ZBTB7A overexpression, had minimal impact.

Together, these findings indicate that coronavirus transcription exhibits complicated, context‑dependent dynamics, influenced by factors including passage history, infection time, viral genetic background, and specific environmental cues. The regulatory mechanisms governing these shifts warrant further investigation.

### Divergent sequence pairs underpin non-canonical transcription with pan-coronavirus distance dependency

We systematically mapped canonical and non-canonical discontinuous transcription events in PEDV- and PDCoV-infected cells using RNA-seq (Fig. 1B). In PEDV-infected Vero cells, we identified 144,832 reads spanning canonical junctions and 159,290 reads spanning non-canonical junctions (Fig. 2C). Canonical discontinuous transcription occurs between pairs of conserved TRSs, exemplified by the α-coronavirus consensus motif AACTAAAC (Yang et al, 2021b). Intriguingly, these non-canonical junctions were mediated by divergent sequence pairs. Here, divergent sequence pairs are defined as genomic region pairs with high intra-pair sequence similarity, even though the two sequences are not globally conserved. Across different genera of coronavirus, these pairs showed significantly higher intra-pair sequence similarity compared to randomly selected sequence pairs (Appendix Fig. S7; one-tailed Wilcoxon rank-sum test, *p* < 0.001). For instance, three distinct sequence pairs drove non-canonical junctions at varying genomic distances; each pair comprised six consecutive identical bases, showing perfect intra-pair conservation but no inter-pair homology (Fig. 2C). These divergent sequence pairs are therefore designated as TRS-like sequences due to their capacity to drive discontinuous transcription analogous to canonical TRSs.

Non-canonical junctions exhibited a pan-coronavirus U-shaped distance dependency (Fig. 2D), and the same pattern was observed after normalization by total genome size (Appendix Fig. S7F). In our PEDV RNA-seq sample, 91.88% of events spanned long-range (>22 kb), 3.13% were mid-range (1.7–22 kb), and 4.99% short-range (<1.7 kb) genomic distance (Fig. 2C,D). This pattern extended to α- (PEDV), β- (SARS-CoV-2), γ- (IBV), and δ-coronaviruses (PDCoV), where long-range (49.84%) and short-range (31.46%) junctions predominated over mid-range (18.70%) events among all RNA-seq samples (Fig. 2D,E; Appendix Fig. S8A; Appendix Table S1). More specifically, non-canonical junctions predominantly form between the N gene and ORF1a over long genomic distances and cluster between structural protein-coding regions at close distances, with both types recurring across diverse coronavirus genera (Appendix Fig. S8B).

### TRS-B and TRS-L flanking regions exhibit RNA–RNA interactions with the same genomic direction

**Figure 3:**
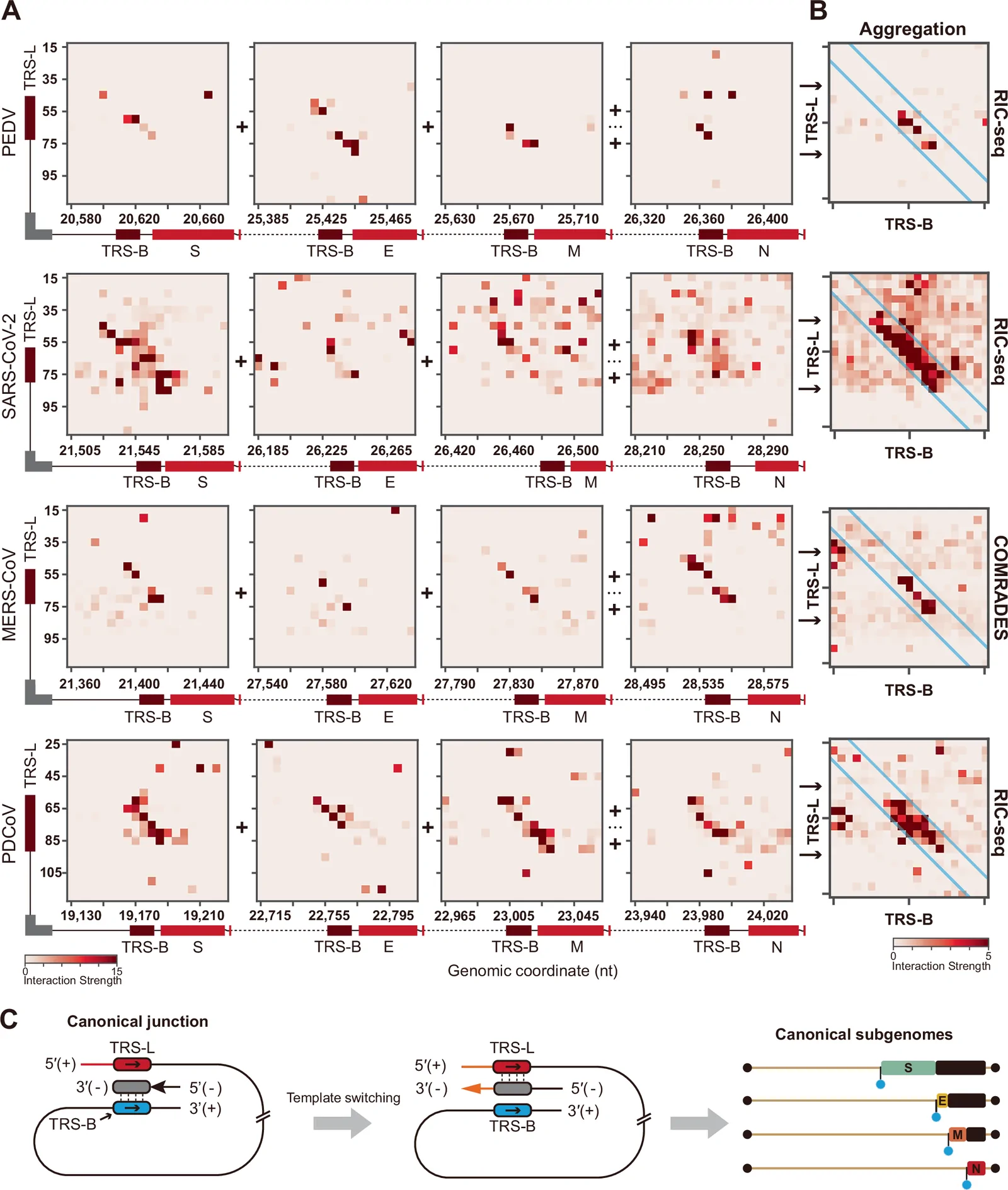
Shared RNA–RNA interaction patterns in canonical sgRNA synthesis across coronaviruses. (**A**,** B**) Heatmaps of genomic interactions between TRS‑B and TRS‑L flanking regions (±50 nt) for individual genes (**A**) and aggregated genes (**B**). For each coronavirus, heatmaps are shown for representative genes (S, E, M, N). “+” symbols between adjacent heatmaps indicate genes included in aggregation; “+ … +” denotes additional genes (e.g., ORF3a, ORF6, ORF7a, ORF7b, and ORF8) not individually displayed due to space and variable gene numbers. The arrow from (**A**) to (**B**) represents aggregation into the APA plot (**B**). Data from RIC-seq (PEDV, SARS-CoV-2, and PDCoV) and COMRADES (MERS-CoV). RNA‑seq samples for junction classification were from our matched experiments (PEDV and PDCoV) or public datasets with similar conditions (SARS‑CoV‑2 and MERS‑CoV). All canonical junctions (involving TRS‑L) were included. The APA heatmap sums interactions centered on all canonical junction‑flanking regions (100 nt windows). Two parallel diagonal lines highlight same‑direction (parallel) interactions. (**C**) The model for canonical sgRNA formation: same‑direction RNA–RNA interactions between the TRS-L and TRS-B flanking regions result in a pair of TRSs being in spatial proximity; this alignment facilitates RdRp template switching from TRS-B to TRS-L during sgRNA template synthesis; ultimately, canonical sgRNAs are synthesized by transcription from these templated strands. Arrows located at the ends of the genome or within the TRS regions indicate the 5′‑to‑3′ direction of the genomic RNA for both positive and negative strands. Source data are available online for this figure.

With the data derived from RIC‑seq (PEDV, SARS‑CoV‑2, PDCoV) and COMRADES (MERS‑CoV), we mapped these interactions within ±50 nucleotides of canonical junction sites (Fig. 3A,B). Based on the patterns observed in interaction heatmaps, the orientation of RNA–RNA interactions can be inferred: diagonal enrichment indicates interactions between regions in the same genomic direction (parallel orientation), whereas anti‑diagonal enrichment indicates interactions in convergent (antiparallel) orientations. Integrated analysis of transcriptome and RNA–RNA interaction data across four coronaviruses revealed that RNA–RNA interaction hotspots preferentially occur between TRS‑L and TRS‑B flanking regions sharing the same genomic direction, as illustrated by heatmap visualization showing pronounced diagonal enrichment of interaction frequencies (Fig. 3A). Aggregate peak analysis (APA) (Rao et al, 2014) confirmed that these interactions consistently aligned with the same genomic directionality (Fig. 3B).

For comparison, we constructed control APA heatmaps based on randomly selected genomic site pairs matched to the canonical junction sites in number and inter-site distance distribution. The canonical junction APA heatmap showed central enrichment, whereas the control heatmap (Appendix Fig. S9A) did not, and the difference was statistically significant (Appendix Fig. S9B; one-tailed *t*‑test, *p* < 0.001). This directional interaction pattern was independently validated by multiple methods (COMRADES, SPLASH and simplified-SPLASH) in SARS-CoV-2 (Appendix Fig. S9C,D).

The consistent diagonal enrichment in APA heatmaps (Fig. 3A,B) indicates that these regions are in close contact, which may facilitate template switching during sgRNA synthesis. We propose that these interactions mediate physical proximity of TRS-L and TRS-B, which may promote RdRp template switching during sgRNA synthesis (Fig. 3C). The molecular basis of this proximity—whether mediated by direct RNA–RNA interactions, RNA-binding proteins, or replication intermediates—remains to be determined.

### Short-range non-canonical junctions associate with reverse-complementary RNA–RNA interactions

**Figure 4:**
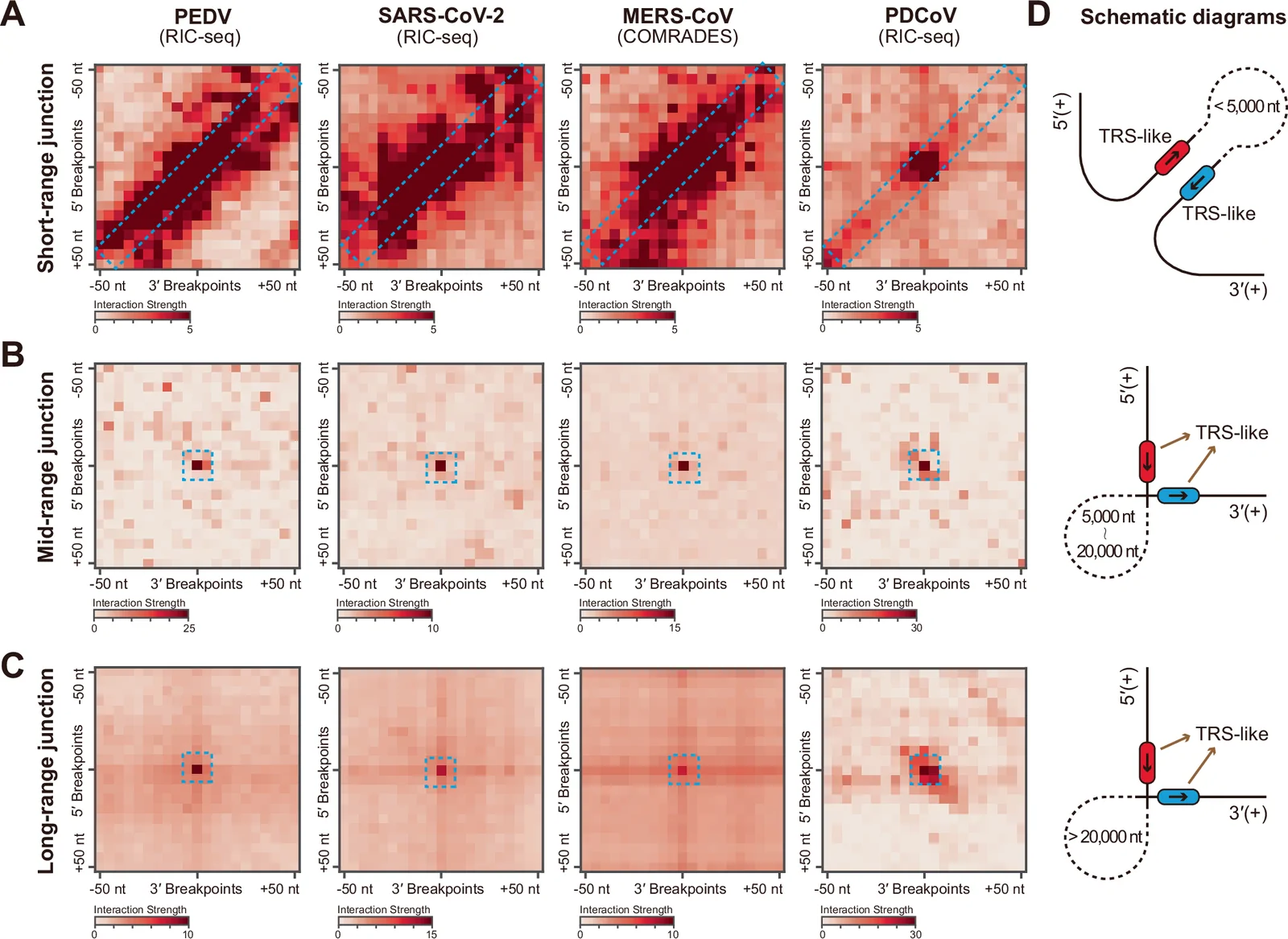
RNA architecture features of different types of non-canonical junctions. **A**–**C** APA heatmaps of aggregated RNA‑RNA interactions from short‑range (**A**), mid‑range (**B**), and long‑range (**C**) non‑canonical junction‑flanking regions of PEDV, SARS‑CoV‑2, MERS‑CoV, and PDCoV. Data from RIC‑seq (PEDV, SARS‑CoV‑2, and PDCoV) and COMRADES (MERS‑CoV). All non‑canonical junctions included. Each heatmap sums 100 nt × 100 nt submatrices centered on each junction site; axes show genomic distance (nt) relative to the center. Red color intensity reflects interaction frequency. Highlighted points (central bright spots) denote positions of enriched RNA‑RNA contacts. Anti‑diagonal lines (running from bottom‑left to top‑right) indicate interactions between genomic regions with convergent orientations. (**D**) Schematic diagrams of RNA architecture underlying non-canonical junction: stem-loops contribute to the formation of short-range junctions; mid-range and long-range junctions are facilitated by distant genomic interactions. Source data are available online for this figure.

On the genome-wide scale, short- and long-range non-canonical junctions are more prevalent than mid-range junctions (Fig. 2D), a pattern consistent with the genome-wide RNA–RNA interaction profiles (Appendix Fig. S4A), suggesting a potential association between non-canonical discontinuous transcription events and RNA architectures. We then investigated the RNA–RNA interaction patterns associated with different types of non-canonical junctions. For PEDV, SARS-CoV-2, and MERS-CoV, short-range junctions exhibit pronounced anti-diagonal enrichments in APA heatmaps (Fig. 4A; Appendix Fig. S10A–C), indicating directional RNA–RNA interactions between genomic regions with convergent orientations. In contrast, this pattern is less discernible in PDCoV, but the interaction strength between short-range junction site-flanking regions is significantly greater than that of controls across these coronavirus genomes (Appendix Figs. S10 and S11).

In contrast to the same genomic-direction interactions observed between TRS-L and TRS-B flanking regions (Fig. 3B), the anti-diagonal enrichment in APA heatmaps reflects convergent-orientation interactions (Fig. 4A). These interactions are likely mediated by reverse-complementary base pairing between flanking regions of short-range junction sites. Further examination reveals that some short-range TRS-like sequence pairs localize within RNA stem-loops stabilized by reverse-complementary base pairing (Appendix Fig. S12). Specifically, in SARS-CoV-2-infected Vero cells (Kim et al, 2020), 5,014 non-canonical junctions were identified between three TRS-like sequence pairs spatially co-localized by a stem-loop spanning nucleotides 29,262–29,870 (Appendix Fig. S12A). These findings indicate that the co-localization of TRS-like sequence pairs with stem-loops could be a crucial factor in promoting short-range non-canonical junctions.

### Mid- and long-range non-canonical junctions correlate with RNA–RNA interaction hotspots

For mid-range non-canonical junctions, a central enrichment signal is observed in the APA heatmaps for PEDV, SARS-CoV-2, MERS-CoV, and PDCoV (Fig. 4B; Appendix Fig. S10A). Similarly, the APA heatmap patterns for long-range junctions in these coronaviruses mirror the mid-range interaction profiles, with PDCoV exhibiting a faint parallel diagonal line (Fig. 4C). Additionally, the average interaction strength between mid- or long-range junction-flanking regions significantly exceeds that of controls across these coronaviruses (Appendix Fig. S11B), suggesting spatial proximity between TRS-like sequence pairs.

Of note, while mid‑range junctions (Fig. 4B) show only a central enrichment, the APA heatmap for long‑range junctions (Fig. 4C) displays a weak cross‑shaped pattern consisting of horizontal and vertical stripes intersecting at the center. Structural analysis revealed that PDCoV exhibits shorter average base‑pairing distances and fewer consecutive paired bases in its predicted stem‑loops compared to the other three coronaviruses (Appendix Fig. S13), consistent with its weaker APA enrichment signals. Despite these differences, the three non-canonical junction types exhibit similar interaction patterns across coronavirus genera (Fig. 4D).

### Short-range non-canonical junctions correlate with genomic deletion hotspots

**Figure 5:**
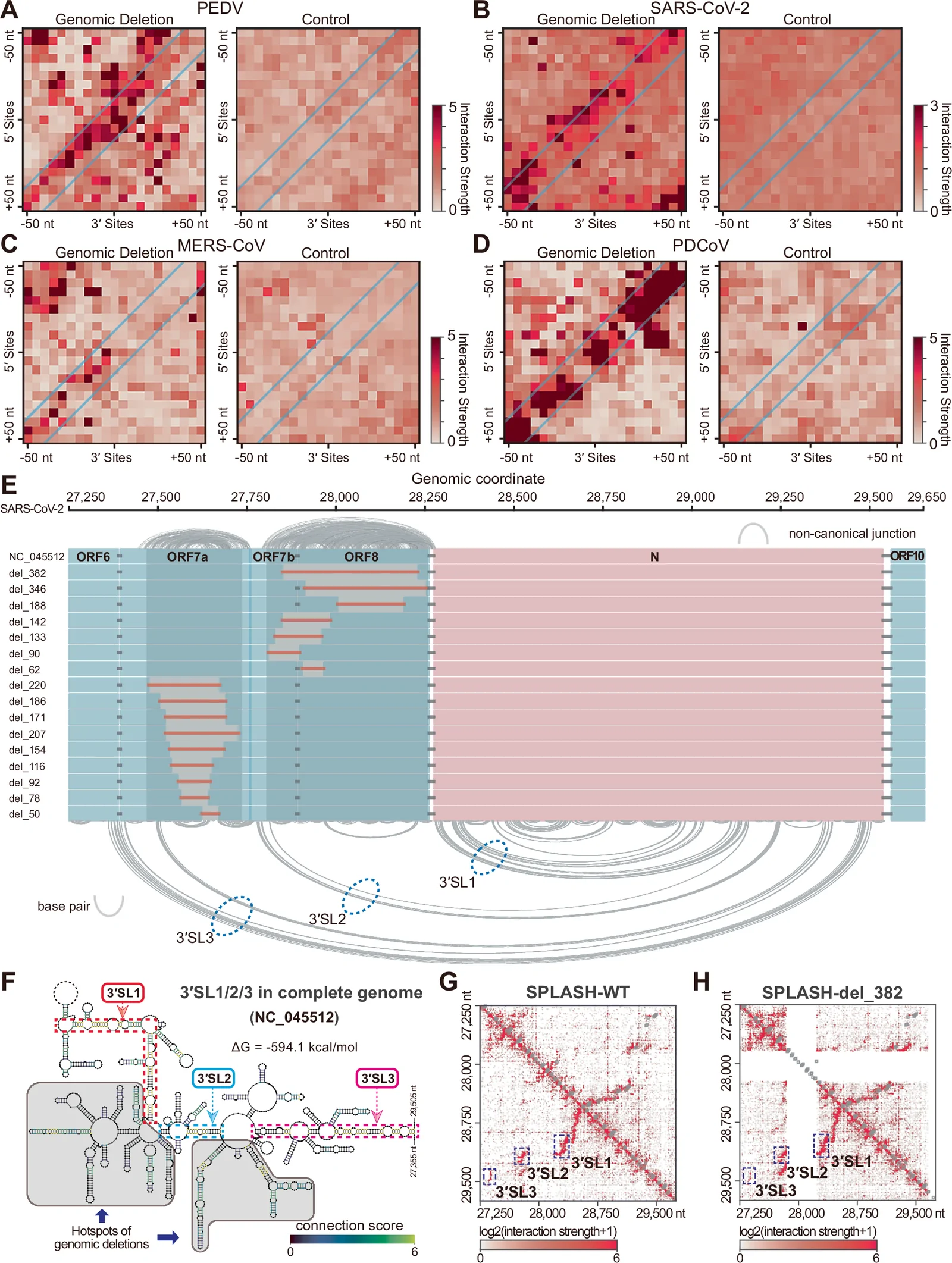
Genomic deletions overlap with stem-loops. (**A**–**D**) APA heatmaps showing aggregated RNA–RNA interactions from deletion flanking regions (50 nt upstream and downstream of each deletion boundary) of PEDV (**A**), SARS‑CoV‑2 (**B**), MERS‑CoV (**C**), and PDCoV (**D**). Data for PEDV, SARS‑CoV‑2, and PDCoV were obtained by RIC‑seq; data for MERS‑CoV by COMRADES. Each heatmap sums 100 nt × 100 nt submatrices centered on each deletion boundary, where the axes represent genomic distance (nt) relative to the boundary center. Color intensity (red) reflects interaction frequency. Anti‑diagonal patterns (running from bottom‑left to top‑right) indicate convergent‑orientation interactions between the two sides of the deletion. Control heatmaps were generated using random genomic site pairs matched to the deletion regions in number and length; a representative control is shown for each virus. (**E**) 3′SL1/2/3 region in the SARS-CoV-2 genome (27,250–29,500 nt) contains 16 genomic deletions and abundant non-canonical junctions. The top panel shows the genomic coordinate scale. The middle panel displays aligned genome structures: the reference genome (NC_045512) with gene annotations is shown at the top; below are representative SARS-CoV-2 variants with deletions. Above the genome structures, arcs depict non-canonical junctions detected by RNA-seq. Below the genome structures, arcs show predicted base pairs derived from the RNA secondary structure of this region. Shaded regions highlight hotspots of frequent genomic deletions. (**F**) The RNA secondary structure of 3′SL1/2/3 in the SARS-CoV-2 genome shows hotspots of genomic deletions, which are shaded. (**G**,** H**) Heatmaps depict the RNA–RNA interactions within the 3′SL1/2/3 region of the SARS-CoV-2 genome, comparing interactions in the complete genome (**G**) with those in the defective genome (**H**). These genomic interactions are captured by SPLASH from Vero E6 cells infected with different SARS-CoV-2 strains. The gray dots in the upper‑right corner indicate predicted base‑pairing interactions derived from the RNA secondary structure of this region (each gray dot represents a paired nucleotide). The larger blue boxes in the lower‑right corner highlight the specific base pairs that constitute the 3′SL1, 3′SL2, and 3′SL3 stem‑loops, which are supported by high‑intensity RNA‑RNA interactions. Source data are available online for this figure.

Through multiple genome alignments, 105, 31, 17, and 8 genomic deletions (>20 nt) have been identified in the genomes of SARS-CoV-2, PEDV, MERS-CoV, and PDCoV, respectively (Appendix Table S2). Genomic deletions were identified independently for each coronavirus species by aligning available genome sequences to a species‑specific reference genome (PEDV: MK584552; SARS‑CoV‑2: NC_045512; MERS‑CoV: NC_019843; PDCoV: KT336560). Notably, the APA heatmaps for these genomic deletion regions show pronounced anti-diagonal enrichment in PEDV and PDCoV, and weaker anti-diagonal enrichment in SARS-CoV-2 and MERS-CoV (Fig. 5A–D).

To determine whether the observed anti-diagonal enrichment is specific to deletion boundaries, we constructed control APA heatmaps using random genomic site pairs matched to the deletion boundaries in number and distance distribution. The interaction strength at deletion ends was significantly higher than random controls for three of the four viruses (SARS-CoV-2, PEDV, and PDCoV; Appendix Fig. S14; one‑tailed *t*-test, *p* < 0.01). Notably, 29.5, 45.2, and 62.5% of deletions in these three viruses overlap with stem-loop regions, respectively (Appendix Table S2; Dataset EV2), suggesting a role for stem-loops in deletion formation. The co-localization of short-range non-canonical junctions with stem-loops (Fig. 4A) further implies a potential role for these junctions in initiating deletions.

We next examined the spatial co-localization between the non-canonical junction sites and the regions where genomic deletions occur. In the genomes of PEDV, SARS-CoV-2, and MERS-CoV, the genomic distance between junction sites and deletion regions is significantly closer than in control regions (Appendix Fig. S15). For example, non-canonical junction sites are enriched within three representative stem-loops in the 3′ -proximal coding region of the genome, designated 3′SL1, 3′SL2, and 3′SL3 (coordinates 27,250–29,500 nt), in the SARS-CoV-2 genome (Appendix Fig. S16A,B). Strikingly, this region also exhibits a significant enrichment of genomic deletions (16 deletions within 2250 nt vs. 105 deletions genome-wide; *p* value = 9.23e-93, Chi-square test) predominantly in the accessory protein genes ORF7a, ORF7b, and ORF8 (Fig. 5E,F; Appendix Table S3). Similarly, a PEDV stem-loop (MK584552.1: 3260–3450 nt) overlapping with 10 ORF1a deletions shows non-canonical junction enrichment (Appendix Fig. S16C; Table S4). These data suggest that short-range non-canonical junctions may drive genomic deletions.

### 3′SL1/2/3 stem-loops bridge TRS-B and ORF10 to drive sgRNA synthesis in SARS-CoV-2

The sequences of 3′SL1/2/3 are highly conserved across different SARS-CoV-2-related genomes, with a sequence identity ranging from 0.84 to 0.93 (Appendix Fig. S17). Predictions of complementary base pairings indicate that these SARS-CoV-2-related SL1s share similar folding patterns and base pair counts (Appendix Fig. S18A). Notably, 3′SL1/2/3 regions are consistently detected in genome-wide RNA interaction maps of SARS-CoV-2 strains, including the full-length genome (hCoV-19/Singapore/2/2020) and an ORF8-deletion variant (hCoV-19/Singapore/12/2020), via SPLASH (Yang et al, 2021a) (Fig. 5G,H; Appendix Fig. S18A). These data indicate the structural preservation of 3′SL1/2/3 across SARS-CoV-2 evolution.

**Figure 6:**
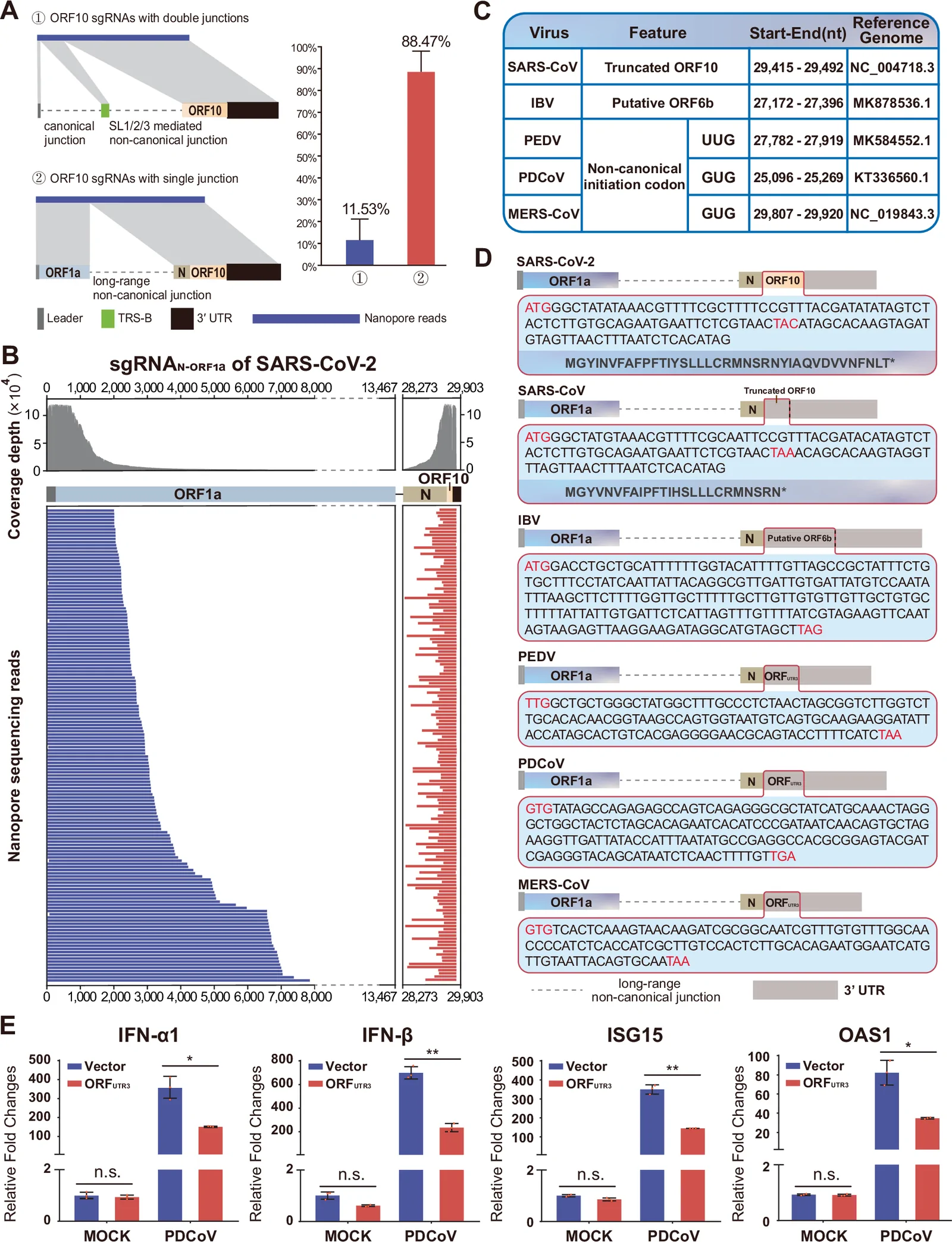
Long-range non-canonical sgRNAs associate with immune responses. (**A**) Two types of non-canonical ORF10 sgRNAs detected from nanopore sequencing reads of SARS-CoV-2-infected Vero cells. One is formed by single junction between N gene and ORF1a, and the other by double junctions including the canonical junction and 3′SL1/2/3-mediated non-canonical junction (left panel). The right panel shows the average proportion of ORF10 sgRNAs arising from single junctions versus double junctions across 49 nanopore samples. Error bars indicate standard deviation. (**B**) Detection of sgRNA_N-ORF1a_ in SARS-CoV-2 by nanopore sequencing. The figure is divided into left and right panels, corresponding to the 5′ UTR–ORF1a region (left) and the N–3′ UTR region (right). Each panel contains three tracks from top to bottom: genomic coverage (gray line), genome structure annotation (5′ UTR in gray, 3′ UTR in black; coding regions labeled), and individual nanopore reads. Each full-length read spans the junction between ORF1a and N; the portion aligning to the 5′ UTR–ORF1a region is shown in blue (left panel), and the portion aligning to the N–3′ UTR region is shown in red (right panel). The two colors together represent a single contiguous sgRNA molecule. (**C**) Characterization of a potential gene identified in the 3′ UTR of different coronavirus genomes. The initiation codon GUG or UUG, with a low translation efficiency, is termed a non-canonical initiation codon here. (**D**) Illustrative representations of sgRNA_N-ORF1a_ in the genomes of different coronaviruses. And the detailed nucleotide sequence of each potential gene is given. (**E**) LLC-PK1 cells were transfected with the ORF_UTR3_ plasmid or empty vector and were then infected with PDCoV. The transcription levels of IFN-α1, IFN-β, ISG15, and OAS1 were monitored by qRT–PCR. Data were presented as mean ± standard deviation from three independent biological replicates (*n* = 3). Statistical significance was assessed using a two-tailed *t*-test comparing ORF_UTR3_-expressing cells to empty vector controls. For ORF_UTR3_ vs. empty vector under PDCoV infection: *p* = 0.0285 (IFN-α1), 0.0073 (IFN-β), 0.0046 (ISG15), and 0.0264 (OAS1). For ORF_UTR3_ vs. empty vector under mock infection: *p* = 0.4172 (IFN-α1), 0.0556 (IFN-β), 0.1275 (ISG15), and 0.7412 (OAS1). Source data are available online for this figure.

Coincidentally, the 5′ parts of 3′SL1/2/3 are located downstream of the TRS-B of genes such as N, ORF7a, and ORF7b, and its 3′ parts are located upstream of ORF10 (Fig. 5E). This spatial arrangement implies that 3′SL1/2/3-mediated non-canonical junctions may enable TRS-B transfer from upstream genes to ORF10 (Fig. 6A). This hypothesis is supported by the identification of sgRNAs with double junctions, containing ORF10, TRS-B of upstream genes and leader sequence, from nanopore sequencing data of SARS-CoV-2-infected Vero cells (Kim et al, 2020) (Fig. 6B; Appendix Fig. S18B). Furthermore, we developed the Corona-mRNA algorithm to calculate transcripts per million (TPM) using nanopore sequencing reads to assess the transcription level of the ORF10 gene in SARS-CoV-2 (Fig. 6A; Appendix Fig. S19A).

Analysis of SARS-CoV-2 nanopore samples revealed that ORF10 sgRNAs are predominantly non-canonical in most samples (Appendix Fig. S19B). In contrast, for all other SARS-CoV-2 genes, canonical sgRNAs vastly outnumber non-canonical sgRNAs (Appendix Fig. S20), confirming that canonical transcription is the dominant mode of viral gene expression. Under the classification rules of Corona-mRNA (see Methods), sgRNAs containing any non-canonical junction are categorized as non-canonical. Therefore, the double‑junction ORF10 sgRNAs are counted as non‑canonical, despite containing a canonical TRS‑L/TRS‑B component (Fig. 6A). Among non-canonical ORF10 sgRNAs, an average of 11.37% arise through double junctions (Fig. 6A; Appendix Fig. S19C; Datasets EV3 and EV4). These findings suggest that the stable 3′SL1/2/3 might act as a structural regulatory element for ORF10 transcription.

### Recurrent N-ORF1a junctions generate non-canonical sgRNAs across coronavirus genera

Besides the generation through double junctions, the majority of ORF10 sgRNAs are formed by a single junction between the N gene and ORF1a among all SARS-CoV-2 samples (Fig. 6A; Appendix Fig. S19C). These sgRNAs, here designated as sgRNA_N-ORF1a_, are distinctly characterized by this unique junction (Fig. 6B). Notably, recurrent junctions between the N gene and ORF1a are also observed in other coronaviruses (Appendix Fig. S21A), resulting in sgRNA_N-ORF1a_ with a 3′ UTR rather than an ORF with a defined function. Nanopore sequencing data for PEDV, MERS-CoV, MHV and HCoV-229E further confirmed the presence of full-length sgRNA_N-ORF1a_ molecules (Appendix Fig. S22; Dataset EV3). Furthermore, reproducible interactions between these two genomic regions were detected across different coronaviruses (Appendix Fig. S21B), suggesting a shared mechanism for sgRNA_N-ORF1a_ biogenesis.

We further investigated the correlation between the transcription level of sgRNA_N-ORF1a_ and RNA–RNA interaction strength using nanopore sequencing data (Chang et al, 2021) and simplified-SPLASH data (Zhang et al, 2021) from SARS-CoV-2 at 24 hpi and 48 hpi (Appendix Fig. S23). A 12.76% decrease is observed in the genomic interaction strength between the N gene and ORF1a at 48 hpi compared to 24 hpi, which corresponds to a significant reduction in the TPM of sgRNA_N-ORF1a_ (Appendix Fig. S23A,B). In contrast, in negative control samples captured by simplified-SPLASH without using RNA ligase, the strength of these interactions increases by 14.68% at 48 hpi compared to 24 hpi (Appendix Fig. S23C). The observed interaction counts (from samples with RNA ligase) and their ratio to the negative control (without RNA ligase) were both significantly lower at 48 hpi than at 24 hpi (Appendix Fig. S23D, two‑tailed Wilcoxon rank-sum test, *P* < 0.001), confirming a true temporal decline in interactions between N gene and ORF1a at 48 hpi. These findings indicate that the transcription level of sgRNA_N-ORF1a_ coincides with alterations in RNA–RNA interaction strength.

### Functional gene emergence within coronavirus sgRNA_N-ORF1a_ 3′ UTRs

Emerging evidence supports the coding ability of ORF10 within sgRNA_N-ORF1a_ of SARS-CoV-2, and ORF10 protein has been identified with the function of suppressing the antiviral innate immune response (Wu et al, 2022; Finkel et al, 2021; Li et al, 2022; Gordon et al, 2020). A truncated version of ORF10, denoted as ORF10_truncated_, is found in the sgRNA_N-ORF1a_ of SARS-CoV with the termination codon (TAA) at 76 nt (Fig. 6C,D). ORF10_truncated_, which shares sequence homology with the 117 nt ORF10 of SARS-CoV-2 (Fig. 6D), is located in the 3′ UTR region; however, its function has not been reported. Notably, a TAC mutation in SARS-CoV-2 ORF10 is proposed to enable functional protein synthesis (Michel et al, 2020; Cagliani et al, 2020), suggesting that 3′ UTR-located sequences may acquire coding potential via nucleotide substitutions.

Except for a putative ORF6b with an undefined function in the 3′ UTR of IBV genome, no known open reading frames (ORFs) were identified in the 3′ UTR of PEDV, PDCoV, and MERS-CoV genomes (Fig. 6C). The 3′ UTR lengths of these coronaviruses are all longer than that of SARS-CoV-2 (Appendix Fig. S24A). Particularly, the 3′ UTR within the PDCoV genome is 383 nt long, surpassing the combined length of the 3′ UTR (155 nt) and ORF10 (117 nt) within the SARS-CoV-2 genome. This suggests that a short gene might exist within these relatively long 3′ UTRs. Non-canonical initiation codons (UUG/GUG) were predicted in MERS-CoV, PEDV, and PDCoV 3′ UTRs, termed ORF_UTR3_ (Fig. 6C,D). Single-base substitutions could convert these to canonical AUG codons, hinting at latent coding capacity.

To investigate the role of ORF_UTR3_ in the antiviral innate immune response, we engineered a variant ORF_UTR3_ of PDCoV by mutating the non-canonical initiation codon GUG to the canonical AUG. Subsequently, LLC-PK1 cells were transfected with this modified ORF_UTR3_ construct (pCAGGS-ORF_UTR3_) alongside a control vector (pCAGGS-Flag) and whole-cell lysates were assessed using quantitative real-time PCR (qRT–PCR). The overexpression of ORF_UTR3_ significantly reduced the transcription of IFN-α1 and IFN-β upon PDCoV infection (Fig. 6E). Furthermore, the mRNA levels of ISG15 and OAS1 in ORF_UTR3_-overexpressing cells are lower compared to the control in the presence of PDCoV (Fig. 6E). An increase in the quantity of N sgRNAs, indicating enhanced viral replication, is also observed in LLC-PK1 cells overexpressing ORF_UTR3_ (Appendix Fig. S24B). The inhibitory effect of ORF_UTR3_ on the antiviral innate immune response is consistent with the reported function of ORF10 in SARS-CoV-2 (Li et al, 2022).

## Discussion

In this study, the RNA–RNA interaction data for different coronaviruses were generated using different methods. Despite these technical differences, all methods share the same principle of capturing inter‑RNA spatial contacts via proximity ligation (Xu et al, 2022; Cao et al, 2021b), without distinguishing genuine interactions (direct base pairing or protein‑mediated) from potential false‑positive signals that may arise from discontinuous transcription intermediates. To minimize such influence, all chimeric reads matching known sgRNA junctions were removed. The key architectural features described above were consistently observed across all methods and viruses, indicating that the observed patterns are robust against potential indirect contributions. Given that the same method (RIC‑seq) was used for PEDV, PDCoV, and SARS‑CoV‑2, the weaker anti‑diagonal signal in PDCoV (Fig. 4C) is more likely to reflect a genuine biological difference. This interpretation is supported by its distinct RNA folding architecture, characterized by shorter base‑pairing distances and fewer consecutive paired bases (Appendix Fig. S13), which may relate to the evolutionary distance of δ‑coronaviruses (Yang et al, 2021b).

Discontinuous transcription serves as a conserved and essential mechanism in coronaviruses, with its regulatory complexity offering critical insights for understanding viral evolution, vaccine design, and novel antiviral target discovery. Early models laid foundational frameworks: Lai et al. proposed the Leader-Primed Transcription model based on murine hepatitis virus (MHV) observations (Lai, 1986), while Sawicki and Sawicki established the discontinuous elongation paradigm (Sawicki and Sawicki, 1995, 1998). Building on these, Sola et al. integrated experimental evidence into a multilevel regulatory framework (Sola et al, 2015), including long-range RNA–RNA interactions that enhance transcriptional efficiency. For instance, complementary pairing between distal and proximal sequences boosts N-gene transcription in TGEV (Moreno et al, 2008). Our integrative analysis of transcriptomes and RNA interactomes across four coronavirus genera further uncovered distinct structural paradigms. For canonical junctions, diagonal enrichment suggests that TRS‑L and TRS‑B are brought into parallel proximity, likely important for template switching during negative‑strand RNA synthesis via the replication‑transcription complex. For short‑range non‑canonical junctions, anti‑diagonal enrichment is consistent with stem‑loop‑mediated antiparallel interactions. Mid‑range non‑canonical junctions show only central enrichment. Long‑range non‑canonical junctions display a weak cross‑shaped pattern, reminiscent of chromatin loop signatures in Hi‑C maps (Rao et al, 2014), suggesting that they may arise from multi‑point contacts mediated by RNA‑binding proteins.

Coronaviruses employ discontinuous transcription to generate canonical sgRNAs, each encoding specific structural or accessory proteins essential for virion assembly and host interaction. However, canonical sgRNAs were not consistently predominant, and the prevalence of non-canonical sgRNAs varied depending on infection phase, viral strain, passage history, and other experimental perturbations (Fig. 2A,B; Appendix Figs. S5 and S6). While some studies attribute non-canonical transcripts to defective genome generation (Kim et al, 2020; Wells et al, 2023), our data show that these sgRNAs are associated with genomic deletions at structural hotspot regions (Fig. 5E; Appendix Fig. S14). We speculate that non-canonical sgRNAs may constitute a pre-adaptive genomic reservoir that could contribute to viral adaptation, though direct evidence remains to be obtained. Our findings also suggest that the dynamic balance between canonical and non-canonical sgRNA production may influence viral evolvability, highlighting the need for further investigation into the regulatory mechanisms that govern this balance.

Deletions are common during the evolution of coronavirus, and previous studies suggest that deletions in SARS-CoV-2 are assumed to arise at template-switching hotspots (Chrisman et al, 2021; Akaishi et al, 2022). We investigated the RNA–RNA interaction patterns in regions where deletions exceed 20 nucleotides in length to uncover the precise RNA architectures that drive genomic deletions in the virus. In the SARS-CoV-2 genome, the 3′SL1-3 region, which is enriched with 16 genomic deletions, overlaps with stem-loops mediating abundant non-canonical junctions (Fig. 5E). Similarly, in the PEDV genome, a region containing ten genomic deletions in ORF1a overlaps with a stem-loop and is also enriched with non-canonical junctions (Appendix Fig. S16C). Based on these findings, we propose that short-range non-canonical junctions mediated by stem-loops may be one of the driving factors for long genomic deletions. Advances in high-resolution RNA architecture detection may enable the exploration of finer details, including the driving force for deletions shorter than 20 nucleotides.

Genomic deletions could be an important way to drive viral evolution and develop attenuated vaccines. ORF8 is a hypervariable gene rapidly evolving among SARS-related coronaviruses, with a tendency to undergo deletions (Zinzula, 2021). Notably, infections with SARS-CoV-2 strains harboring ORF8 deletions are associated with milder clinical symptoms and less systemic release of pro-inflammatory cytokines (Young et al, 2020). Virus attenuation based on passage culture is a traditional strategy for vaccine development (Peng et al, 2023; Li et al, 2021). The caPEDV (JQ023162; isolated from a commercial vaccine of GreenCross, South Korea), which contains a deletion within the ORF3 gene, was obtained by passage culture and showed an attenuated phenotype in pigs (Wicht et al, 2014). Understanding the mechanisms of genomic deletion generation is beneficial for further predicting virus evolution and designing more stable attenuated vaccines.

In SARS‑CoV‑2, while canonical sgRNAs vastly outnumber non-canonical sgRNAs for most viral genes (Appendix Fig. S20), a notable exception is ORF10, which lacks a canonical TRS-B and relies entirely on non-canonical transcription. ORF10 expression involves two distinct non‑canonical mechanisms: a species‑specific pathway mediated by the 3′ SL1‑3 stem‑loops and a cross‑genus pathway that generates sgRNA_N-ORF1a_ through long‑range ORF1a‑N junctions, which is also observed in other coronaviruses to drive expression of 3′ terminal ORFs such as ORF_UTR3_. Insertions, deletions and mutations are the main pathways for viruses to generate new genes and contribute to the virus’s adaptive evolution (Jeronimo et al, 2024; Cui et al, 2019). The recurrence of sgRNA_N-ORF1a_ across coronaviruses could arise from either convergent evolution or co-evolution with the host. Furthermore, overexpression of PDCoV ORF_UTR3_ suppressed the innate immune response (Fig. 6E), similar to the reported function of ORF10 in SARS‑CoV‑2. These observations indicate that non-canonical transcription, despite its low overall abundance, can play a potentially essential role in expressing specific terminal ORFs with immune-modulatory functions. Whether such mechanisms operate in other Nidovirales and whether ORF_UTR3_ influences viral virulence in vivo require further investigation.

## Methods

**Table:** Reagents and tools table

Reagent/resource	Reference or source	Identifier or catalog number
**Experimental models**		
Vero E6 cells	ATCC	CRL-1586
LLC-PK1 cells	ATCC	CL-101
Porcine Epidemic Diarrhea Virus (PEDV) AJ1102 strain	GenBank	MK584552
Porcine Deltacoronavirus (PDCoV) CHN-HN-2014 strain	GenBank	KT336560
**Recombinant DNA**		
pCAGGS-Flag	MiaoLing	P1267
pCAGGS-ORF_UTR3_	This study	Custom-made, no catalog number
**Antibodies**		
**Oligonucleotides and other sequence-based reagents**		
PCR primers q-IFNα1-F: 5′-CTGGCTGTGAGGAAATAC-3′	This study	Custom DNA oligo
PCR primers q-IFNα1-R: 5′-CTGGAGGAAGAGAAGGAT-3′	This study	Custom DNA oligo
PCR primers q-ISG15-F: 5′-CAGCACAGTCCTGTTGATG-3′	This study	Custom DNA oligo
PCR primers q-ISG15-R: 5′-CGTCAGCCAGACCTCATA-3′	This study	Custom DNA oligo
PCR primers q-OAS1-F: 5′-CTGCTATTCCGTGTCTGTCT-3′	This study	Custom DNA oligo
PCR primers q-OAS1-R: 5′-GGTTCCACTCTTGTCTAAGTCT-3′	This study	Custom DNA oligo
PCR primers q-β-actin-F: 5′-ATGCTCCGCTTATTGAAC-3′	This study	Custom DNA oligo
PCR primers q-β-actin-R: 5′-AGGCTCTTACCTGTGATT-3′	This study	Custom DNA oligo
PCR primers ORF_UTR3_-mutagenesis-F: 5′-CCGGGTACCatgtatagccagagagcc-3′	This study	Custom DNA oligo
PCR primers ORF_UTR3_-mutagenesis-R: 5′-CCGCTCGAGtcaacaaaagttgagatta-3′	This study	Custom DNA oligo
PCR primers q-PDCoV-N-F: 5′-TTCGTGGCAATGGAGTTC-3′	This study	Custom DNA oligo
PCR primers q-PDCoV-N-R: 5′-GGATGGAGGAATCGGAGTA-3′	This study	Custom DNA oligo
**Chemicals, enzymes and other reagents**		
TRIzol Reagent	Invitrogen (Thermo Fisher Scientific)	15596026CN
HiScript II Q RT SuperMix for qPCR	Vazyme	R222-01
VAHTS Universal V6 RNA-seq Library Prep Kit for Illumina	Vazyme	NR606-01
Formaldehyde (37%)	Sigma-Aldrich	F8775
Glycine	Sigma-Aldrich	G7126
Trypsin	Gibco (Thermo Fisher Scientific)	25200072
Dulbecco’s Modified Eagle’s Medium (DMEM)	Invitrogen (Thermo Fisher Scientific)	11965092
Minimum Essential Medium (MEM)	Cytiva	SH30024.02
Fetal Bovine Serum (FBS)	Invitrogen (Thermo Fisher Scientific)	16000044
Penicillin-Streptomycin	Invitrogen (Thermo Fisher Scientific)	15140122
**Software**		
fastp	https://github.com/OpenGene/fastp	Version 0.20.0;
STAR	https://github.com/alexdobin/STAR	Version 2.7.10a;
BWA	https://github.com/lh3/bwa	Version 0.7.17;
minimap2	https://github.com/lh3/minimap2	Version 2.17;
RNAfold	https://www.tbi.univie.ac.at/RNA	Version 2.4.18; /
RNAstructure	https://rna.urmc.rochester.edu/RNAstructure.html	Version 6.4;
VARNA	http://varna.lri.fr/	Version 3.9;
Corona-mRNA	https://github.com/WenZi0809/RNA-architecture-underlies-discontinuous-transcription-and-evolution-of-coronavirus	
MAFFT	https://mafft.cbrc.jp/alignment/software/	Version 7.505;
SnapGene	https://www.snapgene.com/	
Python	Python Software Foundation	Version 3.9
RNAeval	http://rna.tbi.univie.ac.at/cgi-bin/RNAWebSuite/RNAeval.cgi	
Juicer Tools	https://github.com/aidenlab/juicer	Version 1.22.01
**Other**		
Illumina HiSeq X Ten Sequencing System	Illumina	
MGISEQ-2000 Sequencer	MGI Tech	

### Cell culture and virus infection

Vero E6 cells were maintained in Dulbecco’s modified Eagle’s medium (DMEM; Invitrogen, CA, USA), supplemented with 10% fetal bovine serum (FBS) and 1% penicillin-streptomycin (Invitrogen, CA, USA). LLC-PK1 cells were cultured in Minimum Essential Medium (MEM; Cytiva), supplemented with 10% fetal bovine serum (FBS) and 1% penicillin-streptomycin (Invitrogen, CA, USA). Cells were cultured at 37 °C in an atmosphere of 5% CO_2_ and 95–99% humidity. Vero E6 cells (1 × 10^7^) were infected with PEDV AJ1102 (GenBank accession: MK584552) at a multiplicity of infection (MOI) of 0.1 for 12 h. LLC-PK1 cells (1 × 10^7^) were infected with PDCoV CHN-HN-2014 (GenBank accession: KT336560) at an MOI of 0.1 for 10 h. Infections were performed in DMEM (for PEDV) or MEM (for PDCoV) supplemented with 7.5 μg/mL trypsin. Vero E6 and LLC-PK1 cells were not authenticated by STR profiling. Both cell lines tested negative for mycoplasma contamination.

### RIC-seq library preparation

At 12 hpi for PEDV and 10 hpi for PDCoV, infected cells were crosslinked with 1% formaldehyde for 20 min at room temperature and then quenched by the addition of glycine. The crosslinked cells were subsequently scraped off the plate and collected by centrifugation at 1000×*g* for 10 min at 4 °C. The subsequent library preparation was conducted as previously described (Cao et al, 2021b), followed by sequencing on an Illumina HiSeq X Ten platform.

### RNA-seq library preparation

At 12 hpi for PEDV and 10 hpi for PDCoV, infected cells were harvested. Total RNA was extracted using TRIzol (Invitrogen, USA) according to the manufacturer’s instructions. RNA-seq libraries were constructed from 2 μg of RNA using the VAHTS Universal V6 RNA-seq Library Prep Kit (Vazyme, CHN), and subsequently sequenced on the MGISEQ-2000 platform with paired-end 150 bp reads.

### Plasmid constructs

Total RNA was extracted from PDCoV-infected LLC-PK1 cells using TRIzol. First‑strand cDNA was synthesized using a reverse transcription kit (HiScript II Q RT SuperMix, Vazyme). The ORF_UTR3_ sequence was then mutated to convert non-canonical start codons (UUGs) to canonical start codons (AUGs) using targeted mutation primers. After mutation, the mutated ORF_UTR3_ was cloned into the pCAGGS-Flag vector. All enzymes used for the cloning procedures were purchased from Vazyme, Nanjing, China.

### Overexpression of ORF_UTR3_ and qRT–PCR

The pCAGGS-ORF_UTR3_ and pCAGGS-Flag were transfected into LLC-PK1 cells, respectively, and the cells were infected with 0.1 MOI of PDCoV 24 h later. Total intracellular RNA was extracted from whole-cell lysates, and mRNA was quantified as described previously (Li et al, 2022). And qRT–PCR was used to detect mRNAs of IFN-I genes, IFN-stimulated genes and viral N gene. The β-actin gene was used as an internal reference to ensure relatively accurate mRNA expression and to avoid systematic and random errors during sample processing. No blinding was applied during the experiments.

### Processing of RNA–RNA interactome

The pipeline developed for RIC-seq data analysis (Cao et al, 2021b) was employed to uniformly process experimental data from RiC-seq, COMRADES, SPLASH, and simplified-SPLASH. These data consist of proximity‑ligated RNA fragments that are sequenced to generate paired‑end reads.

In such data, intra‑RNA interactions are defined as read pairs arising from random self‑ligation within the same RNA molecule (Cao et al, 2021b). These are considered experimental background and are not interpreted as genuine spatial contacts. Inter‑RNA interactions are defined as read pairs derived from proximity ligation between two separate RNA molecules, driven by biologically relevant mechanisms (e.g., RNA‑binding proteins or base‑pairing). Such inter‑RNA reads are specifically labeled with pCp‑biotin and constitute the valid signals for constructing the RNA‑RNA interaction matrix.

The pipeline includes the following five steps:

1. Fastp (Chen et al, 2018) is used for quality control, and reads are then aligned to the reference genome using STAR for both standard and chimeric alignments, while unaligned reads are re‑aligned with BWA to maximize chimeric fragment recovery;
2. The alignment outputs from STAR and BWA are merged, and paired reads are identified from the combined output;
3. Considering the existence of intra-RNA and inter-RNA interactions, paired reads are categorized as normal (intra-RNA, from random self-ligation) and chimeric (inter-RNA, representing genuine intermolecular contacts);
4. After identifying the junction sites of chimeric reads, an RNA–RNA interaction matrix in “.hic” format is constructed, while the Knight-Ruiz algorithm is used to balance this interaction matrix to eliminate experimental errors along the viral genome (Cao et al, 2021a);
5. Pseudo-topological associating domains (pTADs) are divided based on the RNA–RNA interactions matrix. Within each pTAD, RNA secondary structure is predicted using RNAstructure (Reuter and Mathews, 2010) using RNA–RNA interactions as constraints.

Importantly, to avoid contamination from transcriptional products, chimeric reads derived from sgRNA junctions are removed. Specifically, a list of junction coordinates from matched RNA‑seq samples is compiled, and any chimeric read whose breakpoints match these coordinates is filtered out. The remaining chimeric reads are used to construct the final RNA–RNA interaction matrix.

### Processing of RNA-seq data and sgRNA junction identification

RNA-seq data were processed using the same alignment and chimeric read extraction pipeline as described above for RNA–RNA interactome data. The key difference lies in the interpretation of the chimeric reads: in RNA-seq, these chimeric reads correspond to sgRNA junctions formed by discontinuous transcription, rather than physical RNA–RNA contacts. Therefore, after quality control and alignment (using both STAR and BWA), we extracted chimeric reads from the RNA-seq data and used them to identify canonical and non-canonical junction sites. The coordinates of these junctions were compiled into a filter list, which was used to remove transcription‑derived reads from the RNA–RNA interactome data (as described in the previous section). The junction lists were also used to calculate the abundance of sgRNAs.

### Processing of nanopore-seq data and workflow of Corona-mRNA

For nanopore sequencing data, a custom pipeline (Corona‑mRNA) was developed to quantify viral gene expression. Raw reads were basecalled and converted to FASTQ format, then quality‑controlled using NanoPlot and NanoFilt (De Coster and Rademakers, 2023). The reference genome was indexed with minimap2 (Li, 2018) (parameter: -d), and reads were aligned to the genome using minimap2 (parameter: -ax splice -c). For the resulting .sam files, the abundance of each viral gene was calculated using Corona‑mRNA as described below:

1. CIGAR (Compact Idiosyncratic Gapped Alignment Report) information is extracted from the “.sam” file and converted into sgRNAs or genomic RNAs information;
2. Screen sgRNAs that contain both the leader sequence, a complete gene reading frame, and the 3′ UTR region; sgRNAs formed by a single junction of TRS‑L to a sequence within 150 nt upstream of the ORF’s transcription start site that resembles the canonical TRS motif are considered canonical, and the rest are non-canonical;
3. The sgRNAs with the potential to express the same protein are grouped into the same category, where the gene closest to the leader sequence in the sgRNAs is considered to be transcribed;
4. Calculate the abundance of mRNAs and TPM of each gene by canonical and non-canonical junctions, and then the results of the two conditions are combined.

### Identification of canonical and non-canonical junctions

We identified canonical and non-canonical discontinuous transcription events across 103 qualified RNA samples with coronavirus infections, selected by viral genome mapping efficiency (>0.5%) and sufficient discontinuous events (>5000 junctions per sample). For canonical junction sites, we screened for pairs of junction sites that TRS-L is involved and the number of junctions exceeded 2000 or 1/100 of the maximum junctions. For non-canonical junction sites, we screened for pairs of junction sites with junctions exceeding 10. For RNA-seq datasets in Appendix Fig. S4A, we classified non-canonical junctions using a uniform criterion, both using 5000 nt and 20,000 nt as the dividing genomic distance.

### Sequence consistency

The transcription regulatory sequences TRS-L and TRS-B consist of approximately six conserved RNA bases (specifically ACGAAC in SARS-CoV-2), which facilitate template switching through complementarity between the nascent RNA and leader TRS-L. Consequently, an investigation was conducted to evaluate the sequence conservation of RNA pairs (6 nt before or after the junction site) involved in the formation of non-canonical junctions across coronavirus genomes.

Sequence consistency is employed to assess the similarity between pairs of positive-sense strand RNAs that have been identified to possess non-canonical junctions between them. The Bio.pairwise2 module in Python is used to evaluate the consistency between two RNA sequences, and “localms” is chosen as the “alignment type”.

Since G is able to form base complementary pairing with U in RNA, C in 3′ RNA can match U in 5′ RNA, and A in 3′ RNA can match G in 5′ RNA. Therefore, we constructed a match matrix “M” for RNA bases (A, U, C and G) alignment as follows:

1 $${{\rm{M}}}=\left|\begin{array}{cccc}1, & -1, & -1, & -1\\ -1, & 1, & 1, & -1\\ -1, & -1, & 1, & -1\\ 1, & -1, & -1, & 1\end{array}\right|$$

The rows and columns of matrix M correspond to the RNA bases A, U, C, G in that order, with rows representing the 5′ base and columns representing the 3′ base. As shown in the matching matrix “M”, one point is given for a match and one point is penalized for a mismatch. In addition, there is a 0.5 penalty point for either opening a gap or extending a gap. Pairs with a local alignment score ≥4 were designated as TRS‑like sequences.

### Aggregate peak analysis (APA)

We perform APA for canonical/non-canonical junctions (under matched experimental conditions for PEDV and PDCoV; under similar experimental conditions for MERS-CoV and SARS-CoV-2) and genomic deletions based on 5 nt resolution RNA–RNA interaction matrices. To measure the aggregate enrichment of a set of junction sites in an interaction matrix, the heatmap of sum of a series of submatrices derived from that interaction matrix are plotted. Each of these submatrices is a 100 nt × 100 nt square centered at a single junction site in the interaction matrix. The resulting APA heatmap shows the total number of interactions that lie within the entire set of junction sites at the center of the matrix. The APA heatmap of junction sites only includes those whose loci are at least 100 nt apart to avoid the effects of proximal RNA–RNA interactions.

To assess whether the observed interaction strength is specific to the test pairs or simply reflects distance-dependent background, we generate a control set of random genomic site pairs. The control set is constructed to have the same number of pairs and the same inter-site distance distribution (binned into 5 nt intervals) as the test set. This control set is subjected to the same APA aggregation procedure, producing a control APA heatmap that represents the null expectation. The observed APA heatmap and the control APA heatmap are then compared using a one-tailed *t*-test applied to the center values (or pixel-wise) of the two heatmaps. Enrichment is considered significant if *p* < 0.05, indicating that the spatial proximity at the test sites is significantly stronger than what would be expected by chance given the distance distribution. Structural analysis of the genomic regions encompassing deletions was conducted using APA, focusing on RNA–RNA interactions within 50-nucleotide regions surrounding the deletion boundaries. Random pairs of genomic sites with equal length to the actual deletion regions were also employed as the control. Most of the deletion sites in coronaviruses are less than 100 nt, so the “observed over expected” matrix, which eliminates the effect of genomic distance, is used to plot the APA heatmap. Points or lines highlighted in the APA heatmap, which indicate the underlying structural pattern between the two sites involved in the junctions or deletions.

### Prediction of RNA secondary structures

The RNA secondary structures of the 5′ and 3′ UTR regions of PEDV and PDCoV were predicted using an adaptive optimization algorithm based on RIC‑seq data, as previously described (Cao et al, 2021a). Briefly, the viral genome was segmented into short structural domains. Each domain was folded independently using RNAstructure, with the most significant RNA‑RNA interactions (≥95th percentile of interaction frequency) applied as folding constraints. Long‑range and full‑genome structures were then progressively assembled by combining the constrained domains. For regions lacking detectable RIC‑seq interactions, structures were predicted based on minimum free energy. The final structures were visualized using VARNA or RNAstructure.

### Identification of genomic deletions

A total of 833 PEDV genome sequences, 1110 SARS-CoV-2 genome sequences, 657 MERS-CoV genome sequences and 213 PDCoV genome sequences were downloaded from NCBI and GISAID. Then, those genome sequences of each virus were aligned using MAFFT. We defined MK584552, NC_004718, NC_019843, and KT336560 as reference genomes for PEDV, SARS-CoV-2, MERS-CoV, and PDCoV, respectively. Finally, manually examined regions with greater than 20 nucleotides missing on other genomes relative to the reference genome are treated as genomic deletions. Thus, deletions are defined relative to the reference genome of each species and are not directly comparable across genera in terms of absolute coordinates.

### Prediction of potential ORFs in the 3′ UTR of coronaviruses

SnapGene software was used to predict potential ORFs in the 3′ UTRs of PEDV, MERS-CoV, IBV, and PDCoV. In order to predict ORFs within the 3′ UTR region, the minimum number of amino acids was set to 10, and GUG or UUG could be used as the initiation codon in addition to AUG. The predicted ORFs were then screened for their location within most of the long-range non-canonical sgRNAs. ORFs that satisfied all three criteria (minimum 10 amino acids, initiation codon as AUG/GUG/UUG, and located within a long-range non-canonical sgRNA) were considered candidate ORFs.

### Public datasets

All public coronavirus transcriptome (RNA‑seq and nanopore) and RNA–RNA interactome (RIC‑seq, COMRADES, SPLASH, and simplified-SPLASH) datasets used in this study were collected from NCBI, EBI, BIG, and OSF. These datasets, along with their accession numbers, experimental conditions, and the specific analyses they contributed to, are listed in a supplementary file (Dataset EV5). Processed data are stored in the CODE database (https://webofgroup.cn/dgwei/hngene/#/index).

## Supplementary information

Appendix

Peer Review File

Dataset EV1

Dataset EV2

Dataset EV3

Dataset EV4

Dataset EV5

Source data Fig. 2

Source data Fig. 3

Source data Fig. 4

Source data Fig. 5

Source data Fig. 6

## Data Availability

The datasets and computer code produced in this study are available in the following databases: RIC‑seq and RNA‑seq data for PEDV and PDCoV: National Genomics Data Center BioProject PRJCA026550 (https://ngdc.cncb.ac.cn/bioproject/browse/PRJCA026550). Analysis scripts: GitHub (https://github.com/WenZi0809/RNA-architecture-underlies-discontinuous-transcription-and-evolution-of-coronavirus). The source data of this paper are collected in the following database record: biostudies:S-SCDT-10_1038-S44320-026-00239-0.
