# Supplementary material for: Cross-genus analysis reveals architecturally programmed sgRNA synthesis patterns in coronaviruses
Source: Mol Syst Biol. 2026 Jul 30;22(11):1771–88. doi: 10.1038/s44320-026-00239-0 (PMC13728164; doi:10.1038/s44320-026-00239-0)
Supplement: Supplementary file 1 [file 44320_2026_239_MOESM1_ESM.pdf]

Appendix for "Cross-Genus Analysis Reveals Architecturally Programmed sgRNA Synthesis Patterns in Coronaviruses"

Table of contents

Appendix Figure S1: Quality control of RIC-seq and RNA-seq libraries for PEDV ………. page 3

Appendix Figure S2: Quality control of RIC-seq and RNA-seq libraries for PDCoV ……... page 4

Appendix Figure S3: Recapitulation of UTR structures using RIC-seq data ………………. page 5

Appendix Figure S4: Statistics of RNA-RNA interaction data and transcriptome data ……. page 6

Appendix Figure S5: Time-dependent changes in canonical versus non-canonical junction proportions across coronaviruses…………………………………………………………. page 7

Appendix Figure S6: Canonical junction proportions under various perturbations……..…… page 8

Appendix Figure S7: RNA sequence consistency of non-canonical junctions in different coronavirus genomes ……………………………………………………………..…… page 9

Appendix Figure S8: Distribution of junction sites in different coronavirus genomes …… page 10

Appendix Figure S9: Aggregation peak analysis (APA) and comparison of interaction strength for canonical junctions ……………………………………………………………………… page 11

Appendix Figure S10. APA for non-canonical junctions of SARS-CoV-2 ……………….. page 12

Appendix Figure S11: APA for non-canonical junctions of PEDV, MERS-CoV and PDCoV…………………………………………………………………………….,,,,.. page 13

Appendix Figure S12: Stem-loops and TRS-like sequence pairs (highlighted in same color) in genomes of different viruses ……………………………………………………….… page 14

Appendix Figure S13: Comparison of RNA secondary structure properties across four coronaviruses …………………………………………………………………………..… page 15

Appendix Figure S14: Interaction strength comparison for genomic deletions in different coronaviruses ………………………………………………………………………… page 16

Appendix Figure S15: Distance between genomic deletion sites and non-canonical junction sites ……………………………………………………………………………………… page 17

Appendix Figure S16: RNA secondary structures and non-canonical junctions of SARS-CoV-2 and PEDV ……………………………………………………………………………….. page 18

Appendix Figure S17: Sequence identity in the 3′ and 5′ parts of 3′SL1-3 across SARS-CoV-2 related strain genomes ……………………………………………………………... page 19

Appendix Figure S18: Stable 3′SL1 and non-canonical ORF10 sgRNAs ………………… page 20

Appendix Figure S19: Transcript levels of ORF10 sgRNAs calculated using Corona-mRNA ……………………………………………………………………………... page 21

Appendix Figure S20: Transcription levels of individual genes in Vero E6 cells at different hpi with SARS-CoV-2 ……………………………………………………………… page 22

Appendix Figure S21: Non-canonical ORF1a-N junctions and RNA-RNA interactions across coronaviruses …………………………………………………………………………… page 23

Appendix Figure S22: Nanopore sequencing validation of sgRNA$_{N\text{-}ORF1a}$ in multiple coronaviruses …………………………………………………………………………… page 24

Appendix Figure S23: Correlation of junction counts with the RNA-RNA interaction strength ……………………………………………………………………………….. page 25

Appendix Figure S24: ORF$_{UTR3}$ in coronaviruses …………………………………………. page 26

Appendix TABLE S1: Proportion of non-canonical junctions at different genomic distances …………………………………………………………………………… page 27

Appendix TABLE S2: Deletions obtained based on multiple sequence alignment ……..… page 27

Appendix TABLE S3: Locations of enriched genomic deletions in SARS-CoV-2 genome (NC_045512.2) ………………………………………………………………………. page 28

Appendix TABLE S4: Locations of enriched genomic deletions in PEDV genome (MK584552) ……………………………………………………………………………. page 29

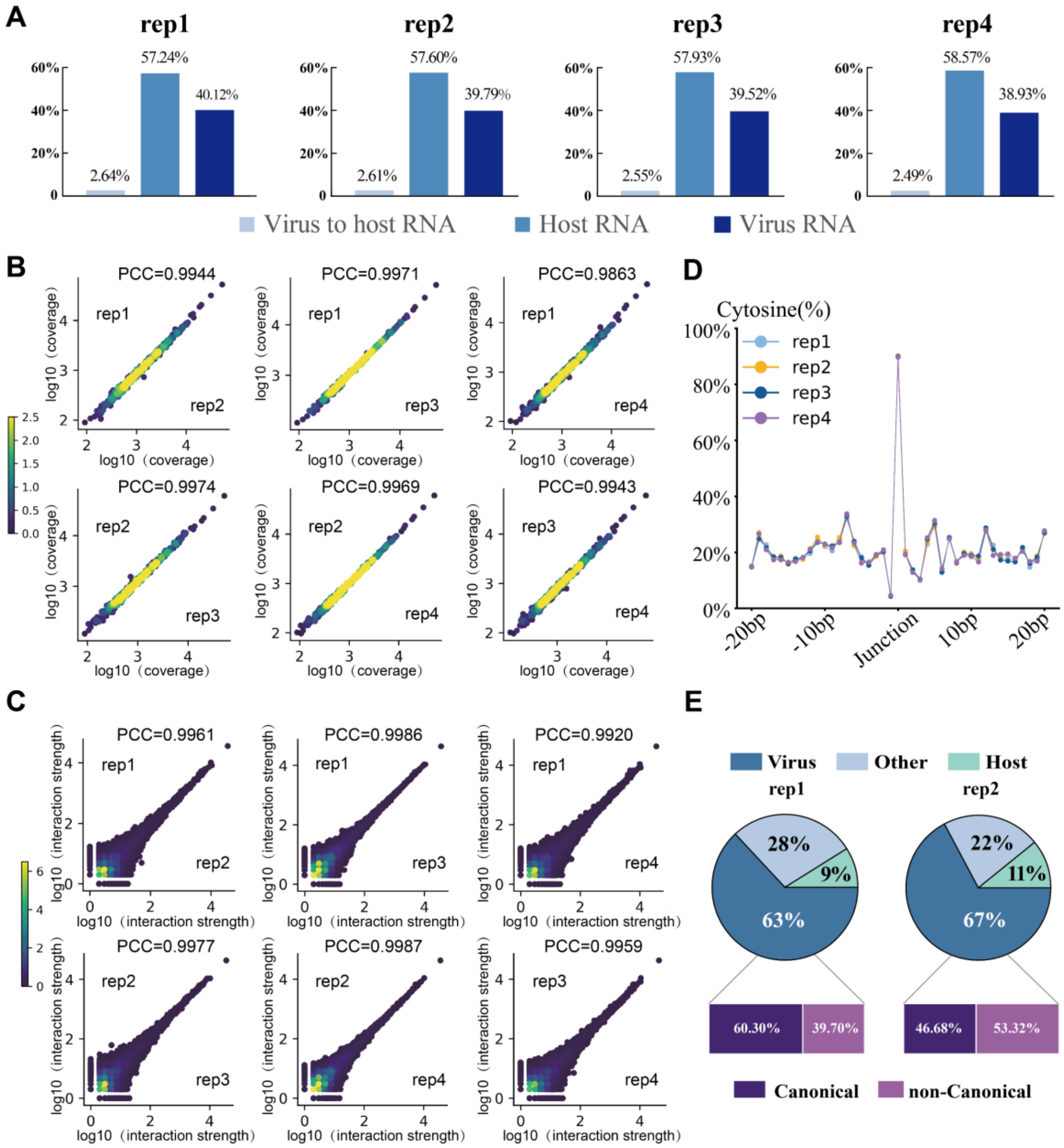

Appendix Figure S1. Quality control of RIC-seq and RNA-seq libraries for PEDV.

A, Read mapping proportions of chimeric reads. Shown are the percentages of chimeric reads mapping to the virus RNA, host RNA and virus-to-host (one end virus, one end host) RNA. **B-C,** Scatter plots for each pair of biological replicates, showing the correlation of chimeric read coverage along the genome (B) and the correlation of interaction strength (C). The Pearson correlation coefficient (PCC) was calculated for each replicate pair. **D,** Cytosine percentage at chimeric read junctions. The horizontal axis is centered at the junction site (position 0), spanning 20 nucleotides upstream and downstream. Each replicate is represented by a distinct color. **E,** RNA-seq data analysis. The upper pie chart shows read mapping proportions (virus, host, other). The lower bar plot shows the proportions of canonical versus non-canonical junctions among virus reads.

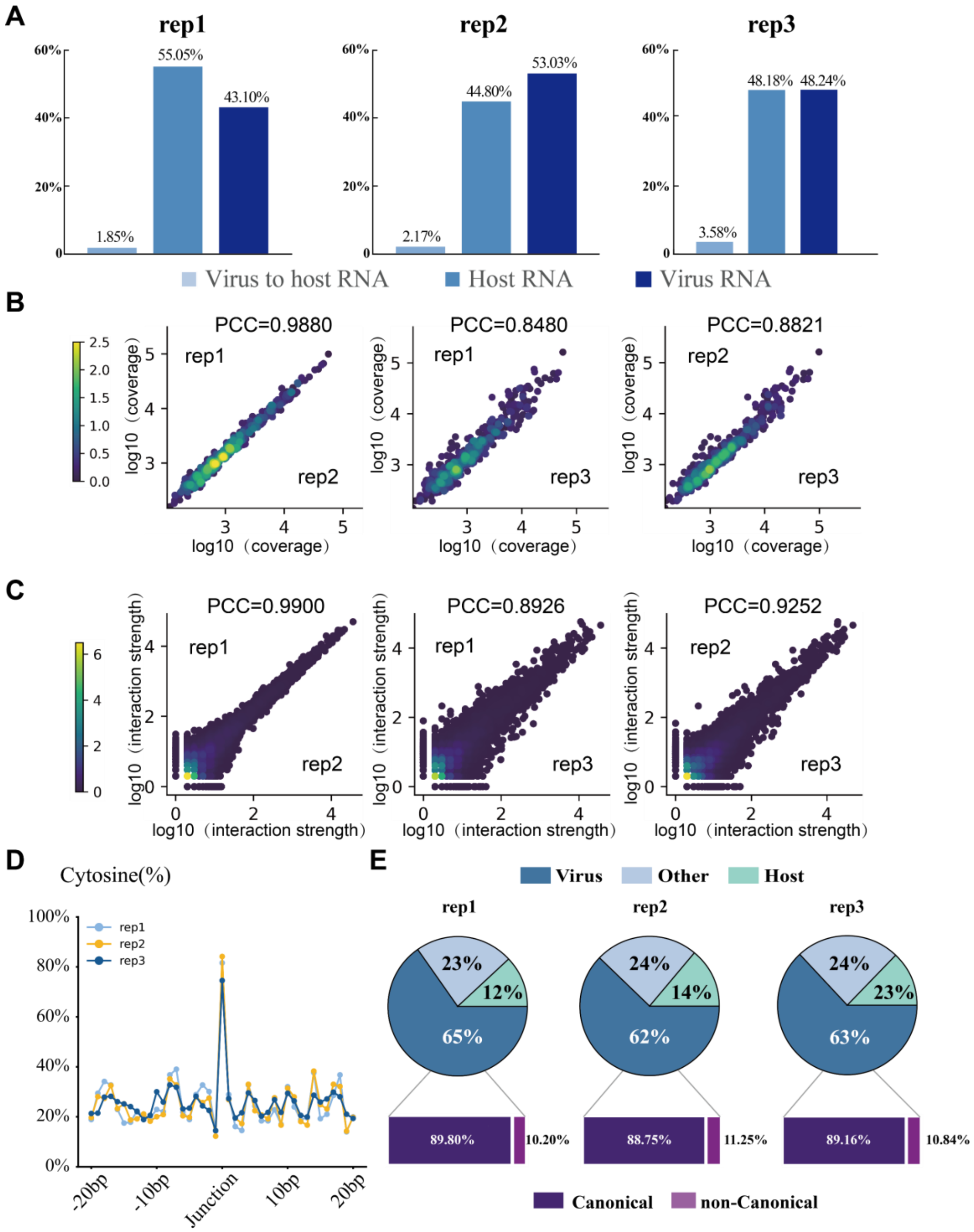

Appendix Figure S2. Quality control of RIC-seq and RNA-seq libraries for PDCoV.

A, Read mapping proportions of chimeric reads. Shown are the percentages of chimeric reads mapping to the virus RNA, host RNA and virus-to-host (one end virus, one end host) RNA. **B-C,** Scatter plots for each pair of biological replicates, showing the correlation of chimeric read coverage along the genome (B) and the correlation of interaction strength (C). The Pearson correlation coefficient (PCC) was calculated for each replicate pair. **D,** Cytosine percentage at chimeric read junctions. The horizontal axis is centered at the junction site (position 0), spanning 20 nucleotides upstream and downstream. Each replicate is represented by a distinct color. **E,** RNA-seq data analysis. The upper pie chart shows read mapping proportions (virus, host, other). The lower bar plot shows the proportions of canonical versus non-canonical junctions among virus reads.

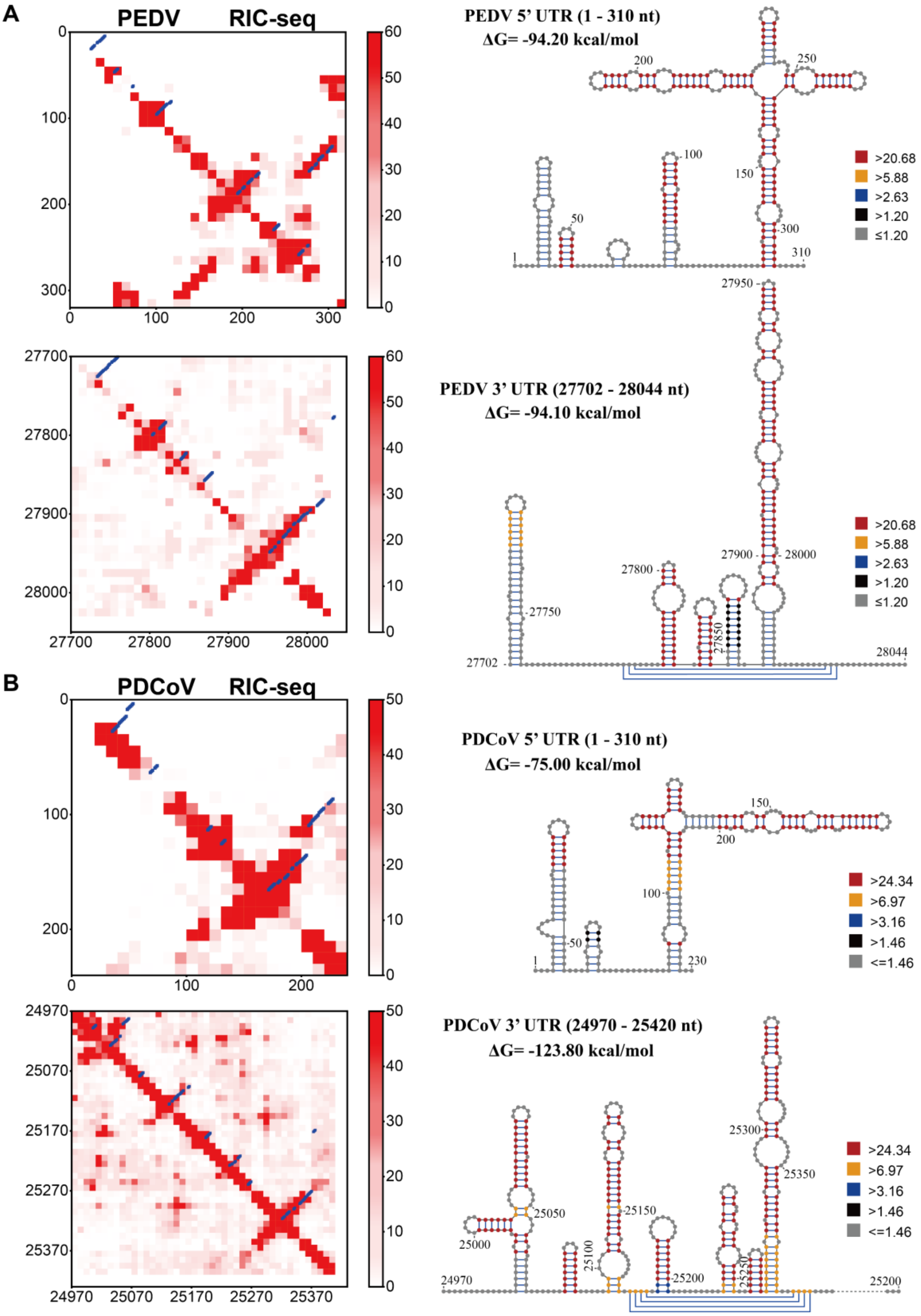

Appendix Figure S3. Recapitulation of UTR structures using RIC-seq data.

A-B, Shown are the 5′ UTR and the 3′ UTR of PEDV (A) and PDCoV (B). For each panel, the left heatmap displays RNA-RNA interaction frequencies along the UTR region, with darker red indicating stronger interactions. Dots above the heatmap mark nucleotide positions predicted to be base-paired, and dot colors correspond to interaction strength. The right panel shows the predicted RNA secondary structure. Bases are colored according to the strength of supporting RNA-RNA interactions.

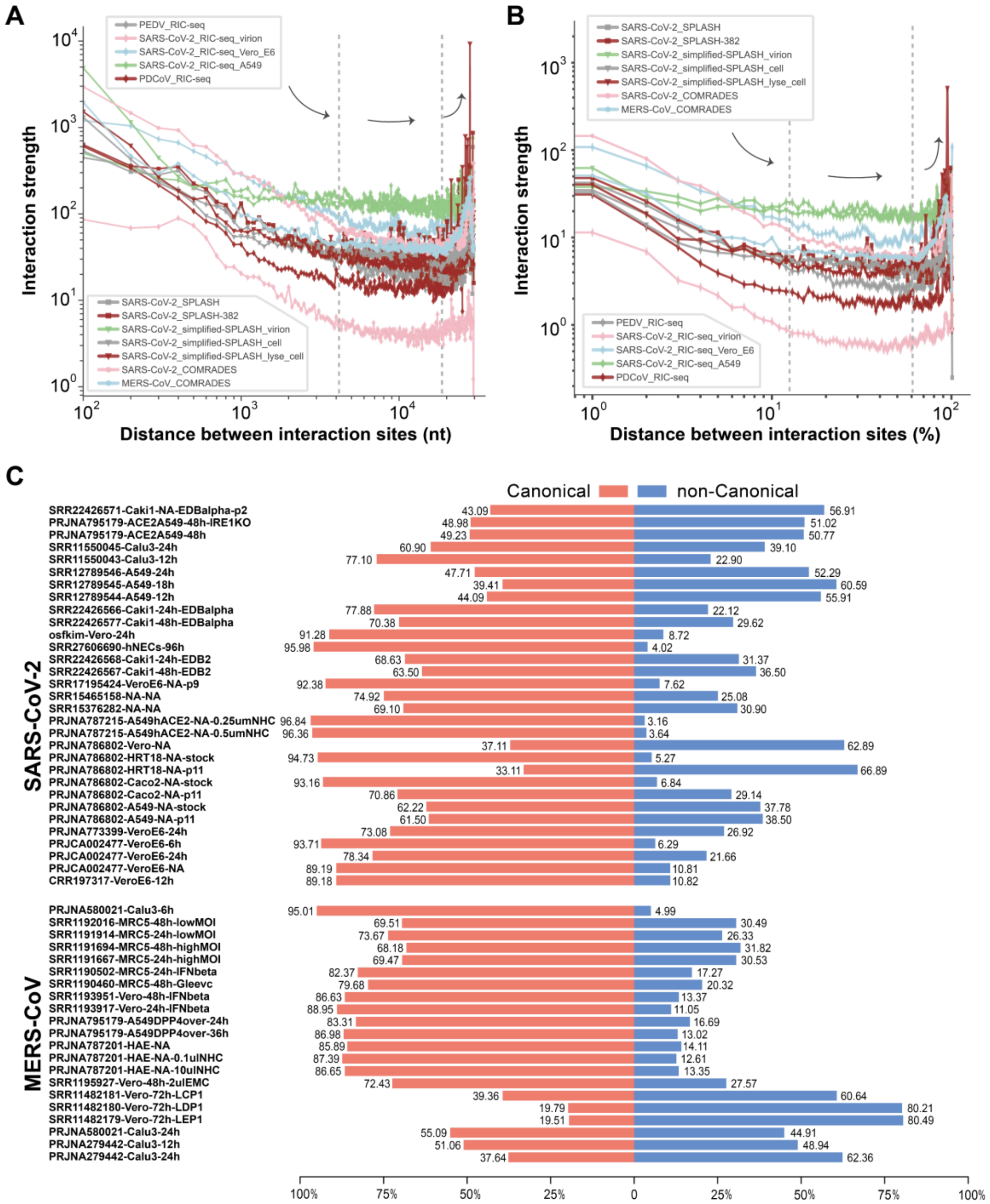

Appendix Figure S4. Statistics of RNA-RNA interaction data and transcriptome data. **A-B**, RNA-RNA interaction strength across a range of distances between pairwise interacting RNAs from different coronaviruses. The x-axis represents absolute genomic distance (kb) in (A) and relative genomic distance (100 equal bins) in (B); the y-axis shows the normalized interaction frequency. Two vertical dashed lines at 5 kb and 20 kb divide the distance range into three regions: short-range, mid-range, and long-range. Arrows indicate the overall trend of interaction frequency. **C**, Proportions of canonical and non-canonical junctions in RNA-seq samples of SARS-CoV-2 and MERS-CoV. The SRA or BioProject accession number is added to the sample name.

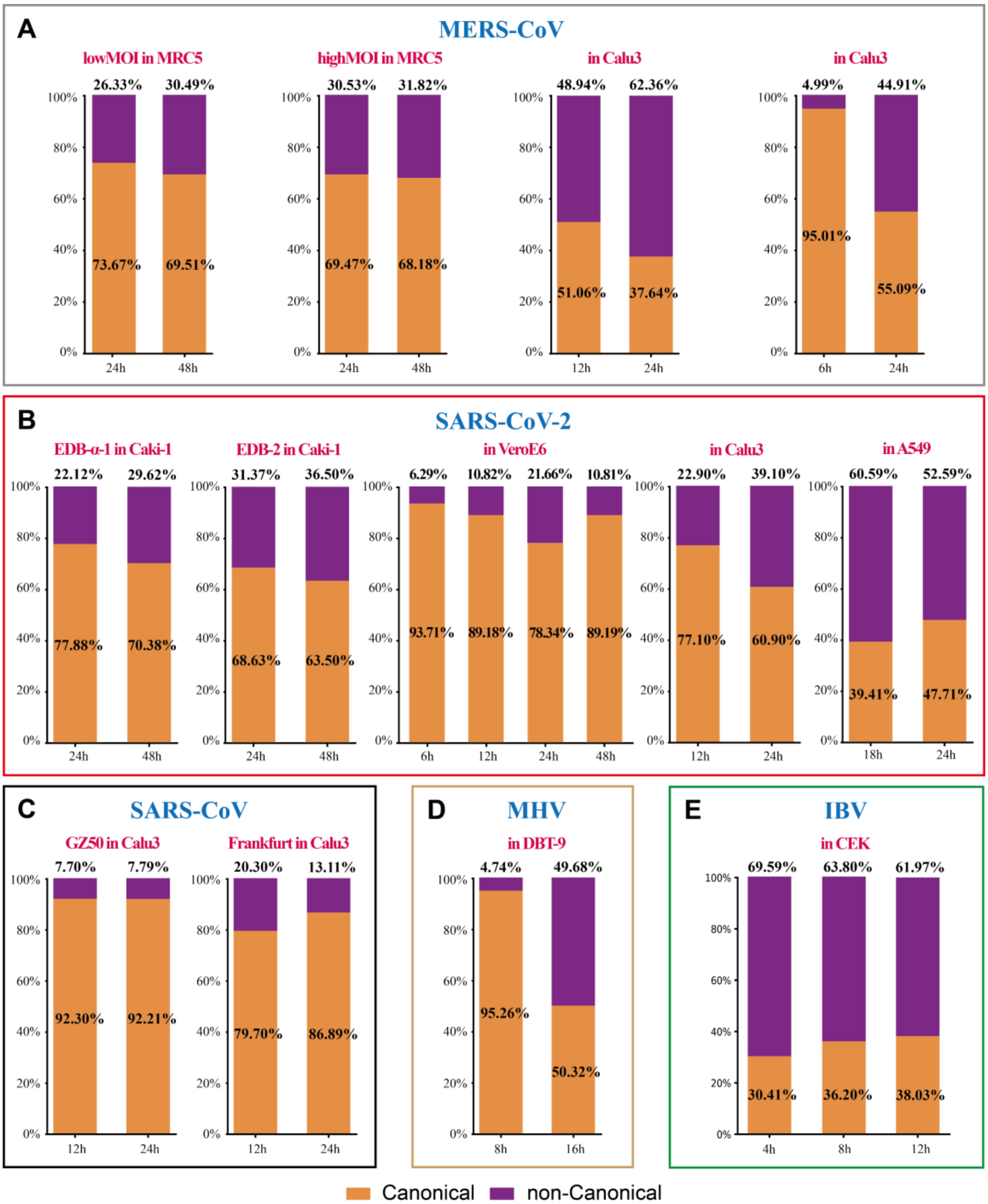

Appendix Figure S5. Time-dependent changes in canonical versus non-canonical junction proportions across coronaviruses.

A-E, Time-course RNA-seq datasets from MERS-CoV (A), SARS-CoV-2 (B), SARS-CoV (C), MHV (D) and IBV (E) were analyzed. For each virus, the proportion of canonical junctions is plotted against hours post-infection (hpi).

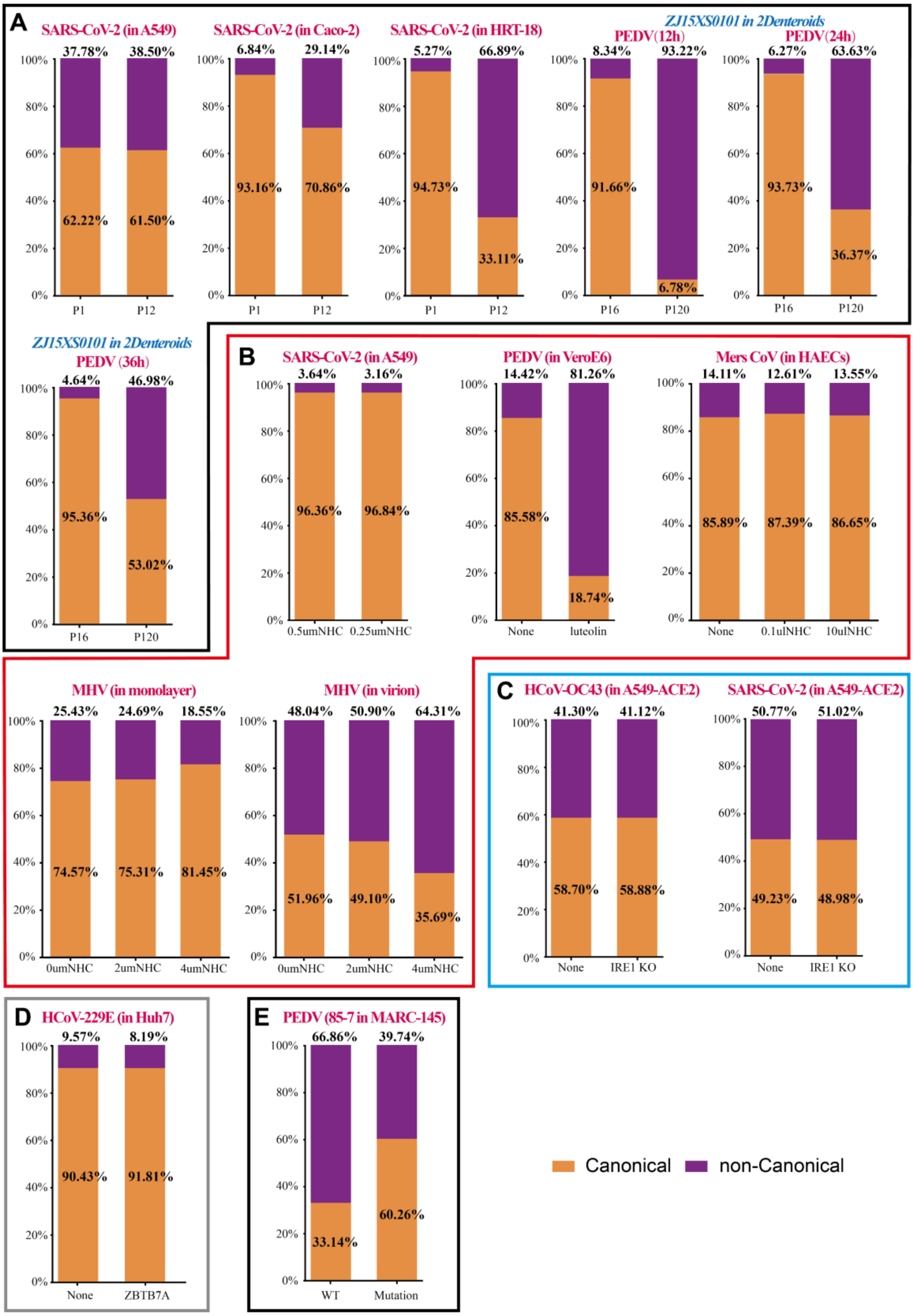

Appendix Figure S6. Canonical junction proportions under various perturbations.

A–E, RNA-seq datasets for low-passage versus high-passage strains (A), drug treatments with NHC (EIDD-1931) and luteolin (B), host gene perturbations of IRE1 knockout (C) and ZBTB7A overexpression (D), and viral mutations (E) were analyzed. For each perturbation, the proportion of canonical junctions is plotted

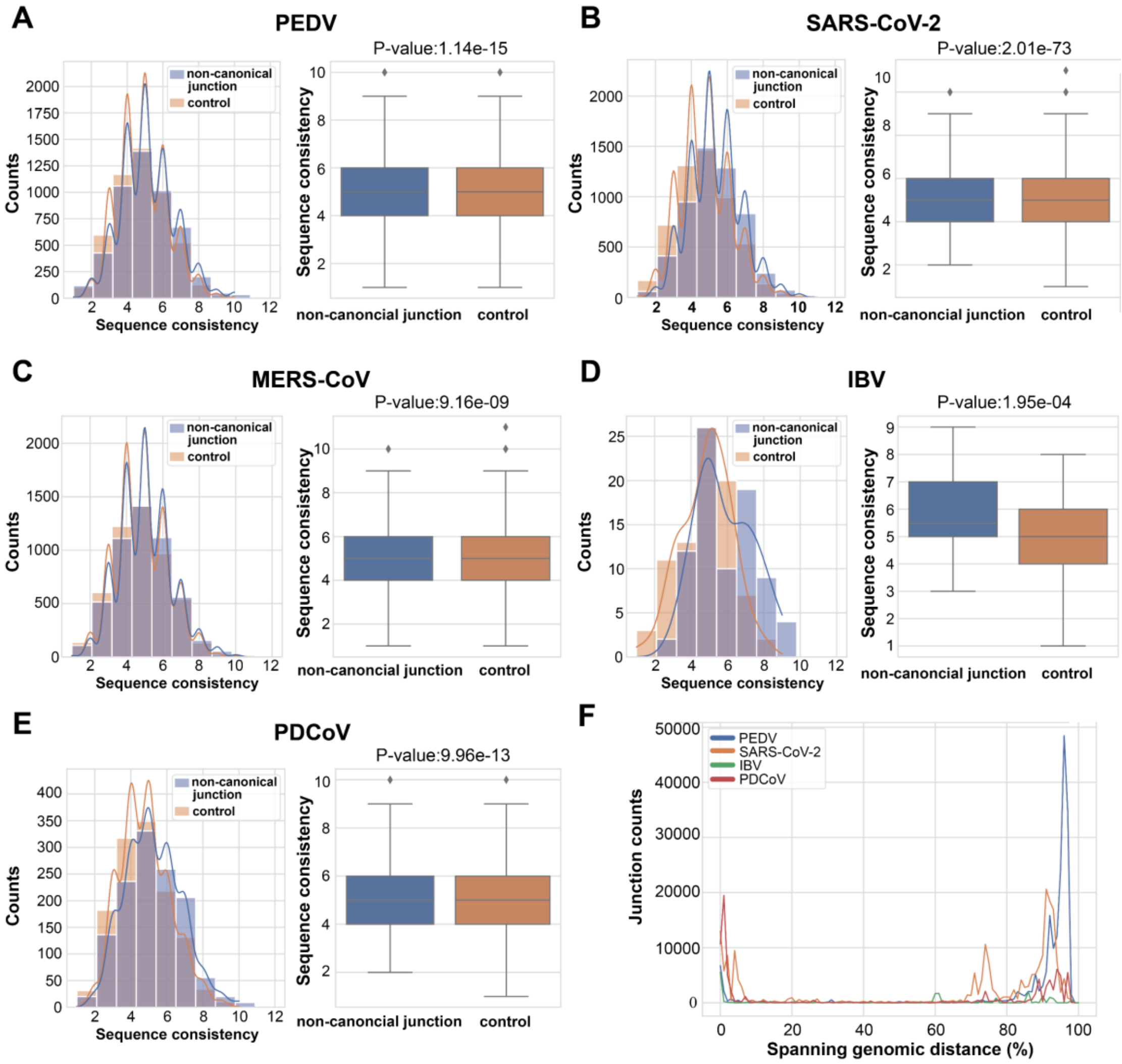

Appendix Figure S7. RNA sequence consistency of non-canonical junctions in different coronavirus genomes.

A-E, Histogram and Boxplot of sequence consistency between pairs of genomic RNAs involved in non-canonical junctions in PEDV (A), SARS-CoV-2 (B), MERS-CoV (C), IBV (D) and PDCoV (E) genomes. The p-value of two-tailed Wilcoxon rank-sum test was calculated for each group. **F**, Distribution of non-canonical junctions across the coronavirus genome. The genome of each virus was divided into 100 equal segments (relative genomic distance). For each segment, the counts of non-canonical junctions were calculated across four coronavirus genera (α, β, γ, δ). Non-canonical junctions predominantly occurred at short and long genomic distances, with fewer junctions in the mid-range.

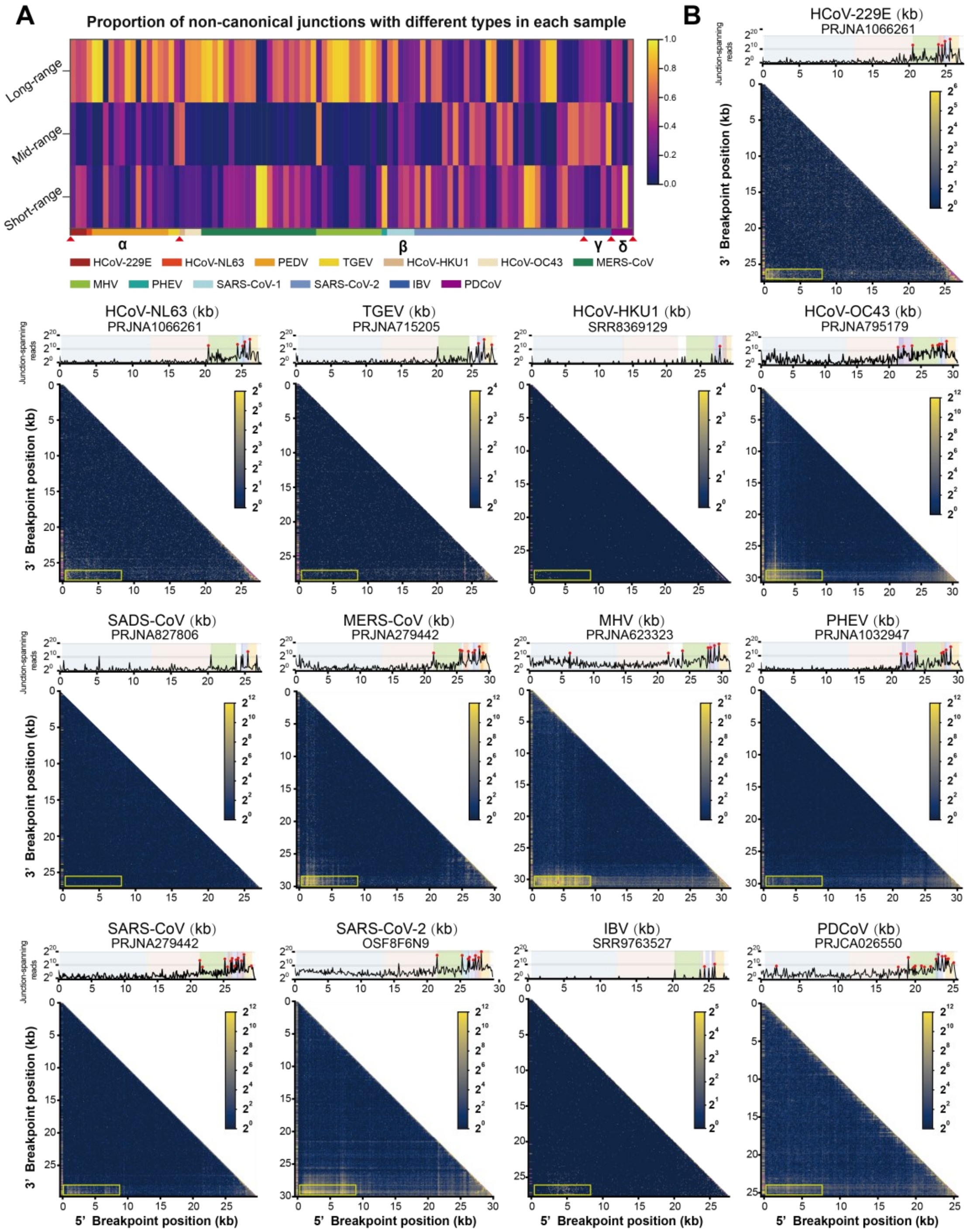

Appendix Figure S8. Distribution of junction sites in different coronavirus genomes.

A, Heatmap depicting the proportion of non-canonical junctions with different types among all RNA-seq samples. **B**, Heatmaps depicting the distribution of junction sites for different coronavirus genomes.

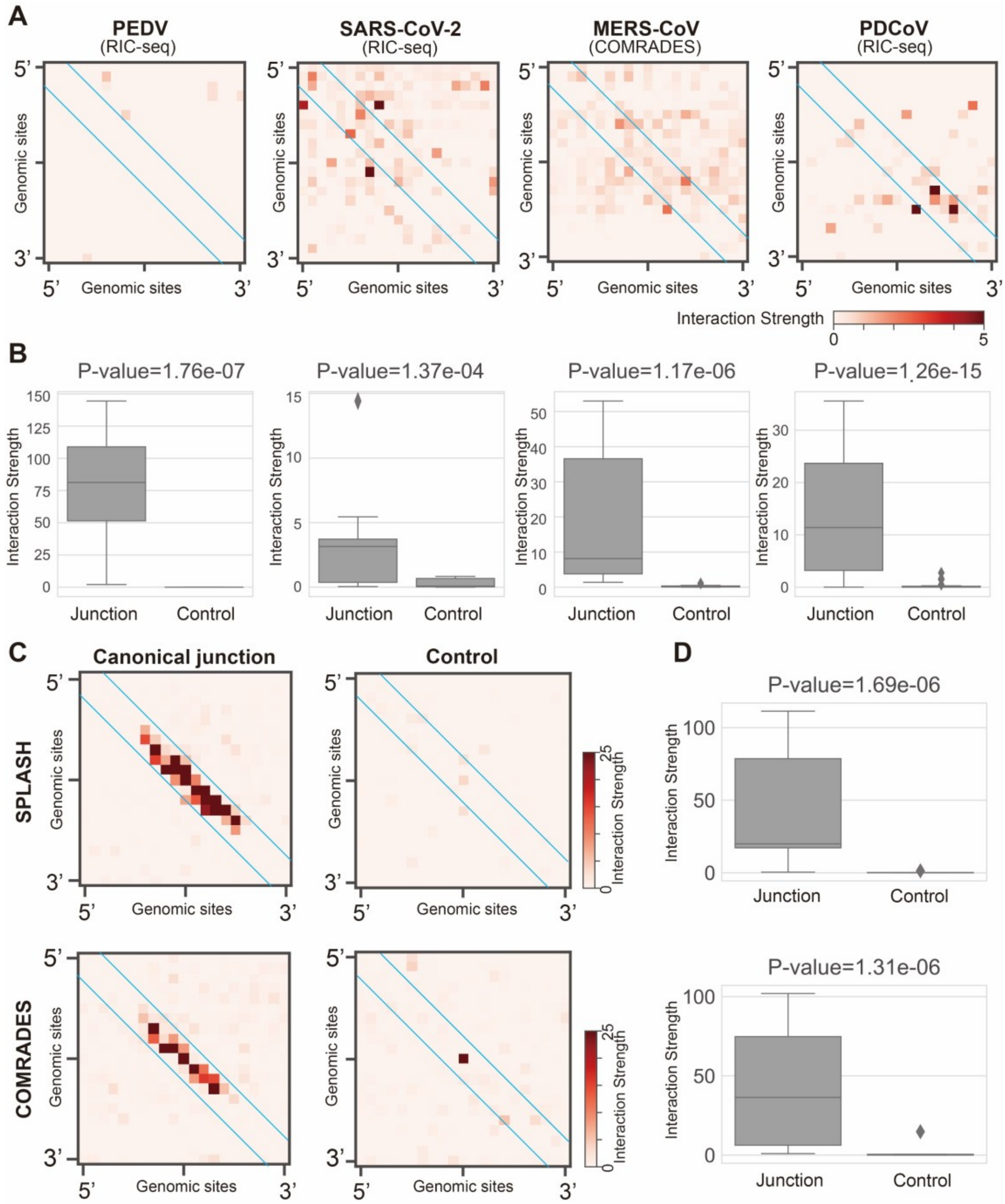

Appendix Figure S9. Aggregation peak analysis (APA) and comparison of interaction strength for canonical junctions.

A, Random pairs of sequences spanning lengths equivalent to those of the canonical junctions were employed as controls. APA plot shows the aggregated RNA-RNA interactions from control groups. RNA-RNA interactions for PEDV, SARS-CoV-2 and PDCoV are detected by RIC-seq, and RNA-RNA interactions for MERS-CoV are detected by COMRADES. **B**, Boxplots depicting the RNA-RNA interaction strengths between canonical junction sites, compared with control groups. Interaction strengths were determined by selecting the regions delineated between the two blue lines in (A). **C**, APA plot shows the aggregated RNA-RNA interactions from canonical junction site-flanking regions and control groups of SARS-CoV-2. RNA-RNA interactions are captured by SPLASH and COMRADES, respectively. **D**, Boxplots depicting the RNA-RNA interaction strengths between canonical junction sites, compared with control groups. Interaction strengths were determined by selecting the regions delineated between the two blue lines in (C). The p-value of one-tailed t-test was calculated for each group.

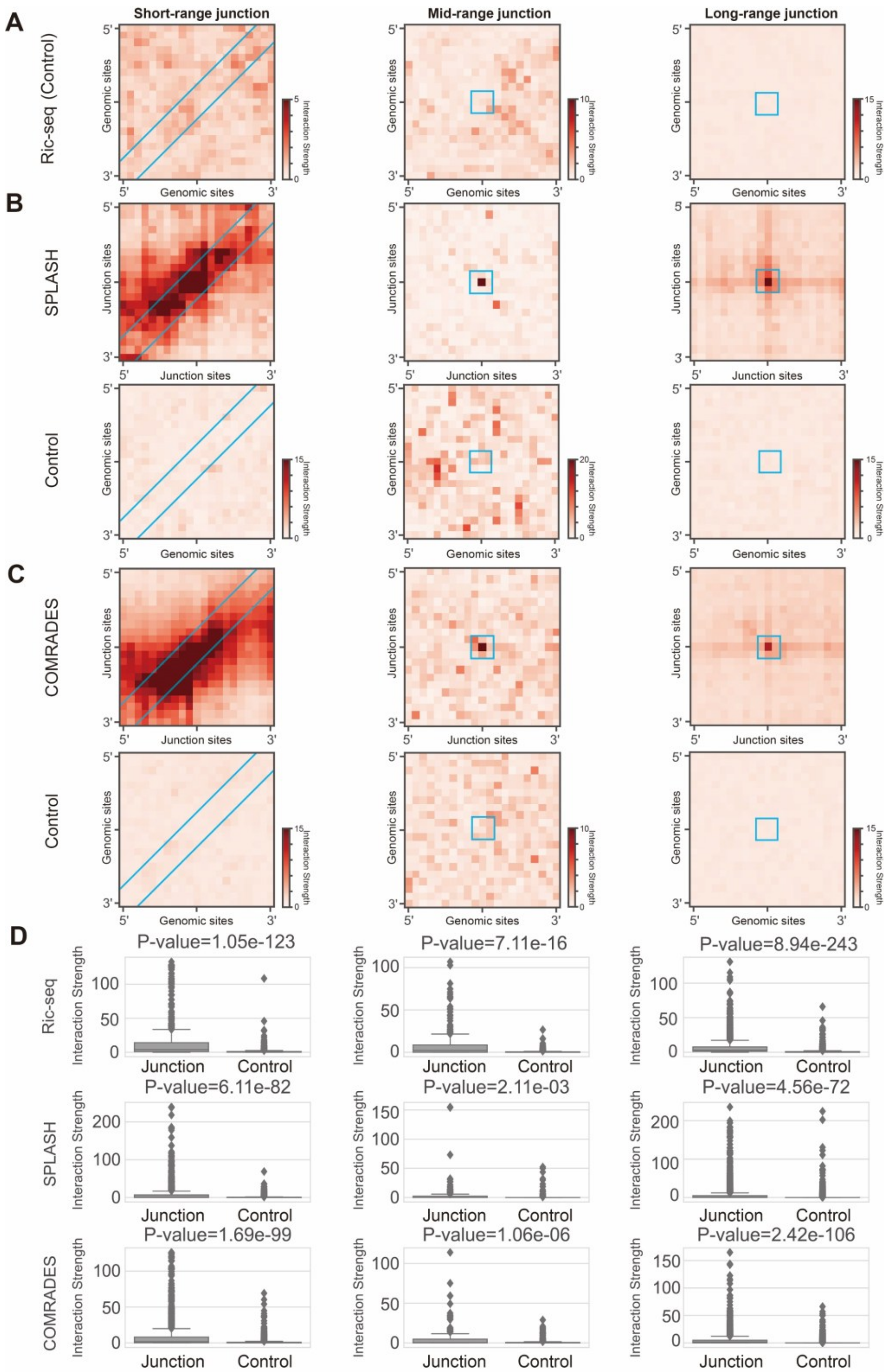

Appendix Figure S10. APA for non-canonical junctions of SARS-CoV-2.

A, Random pairs of sequences spanning equivalent lengths as the non-canonical junctions are employed as the control. APA plot shows the aggregated RNA-RNA interactions from control groups based on RIC-seq data. **B-C**, APA plot shows the aggregated RNA-RNA interactions from non-canonical junction site-flanking regions and control groups based on SPLASH (B) and COMRADES (C) data. **D**, Boxplots of RNA-RNA interaction strengths of non-canonical junctions and control groups. Interaction strengths were determined by selecting the regions delineated between the two blue lines or blue line boxes in Fig. 4 and Appendix Figure S10BC. The p-value of one-tailed t-test was calculated for each group.

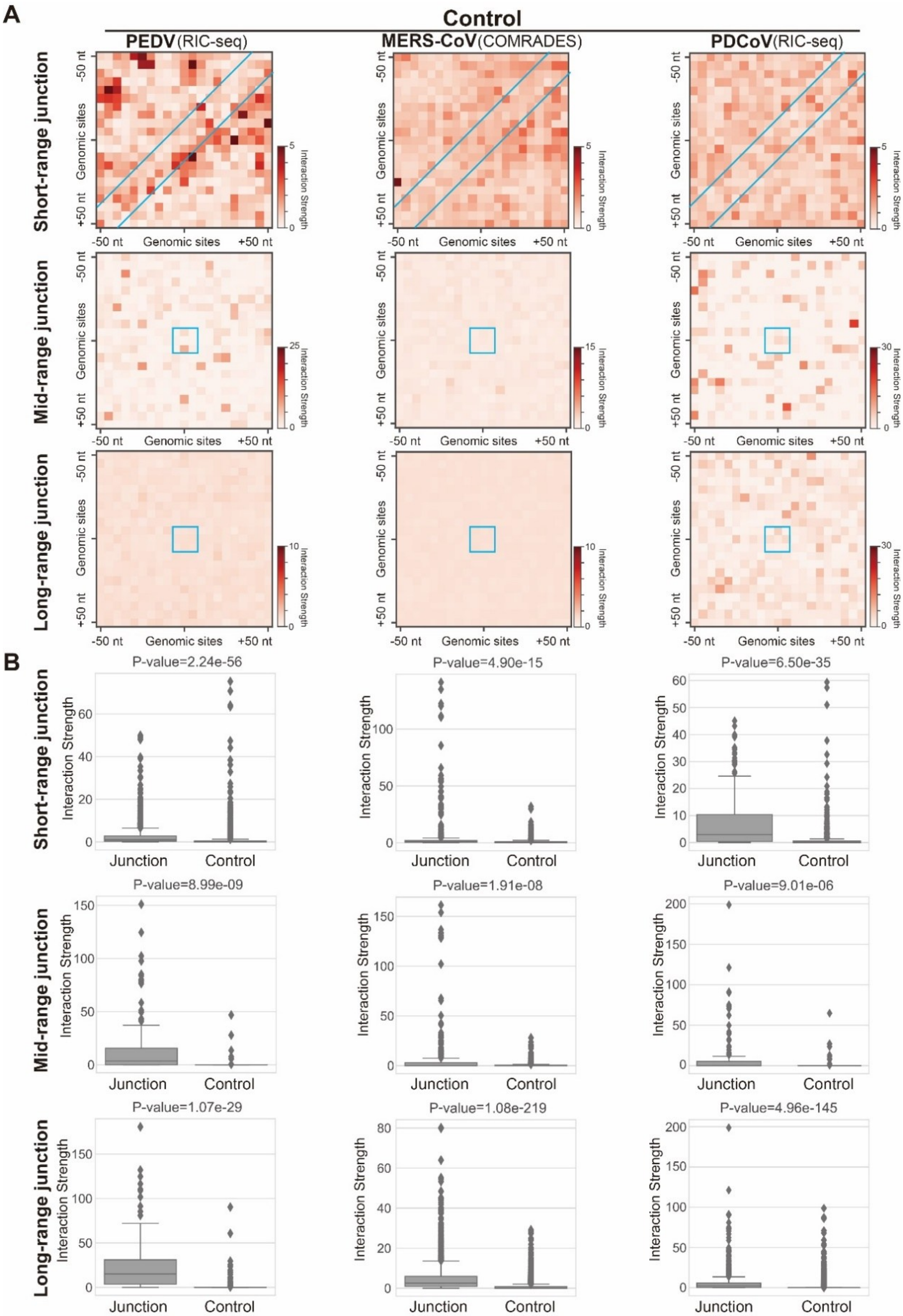

Appendix Figure S11. APA for non-canonical junctions of PEDV, MERS-CoV and PDCoV. **A**, APA plot shows the aggregated RNA-RNA interactions from control groups. **B**, Boxplots of RNA-RNA interaction strengths of non-canonical junctions and control groups. Interaction strengths were calculated from the regions located in the blue lines or blue line boxes in Fig. 4 and Appendix Figure S11 A. The p-value of one-tailed t-test was calculated for each group.

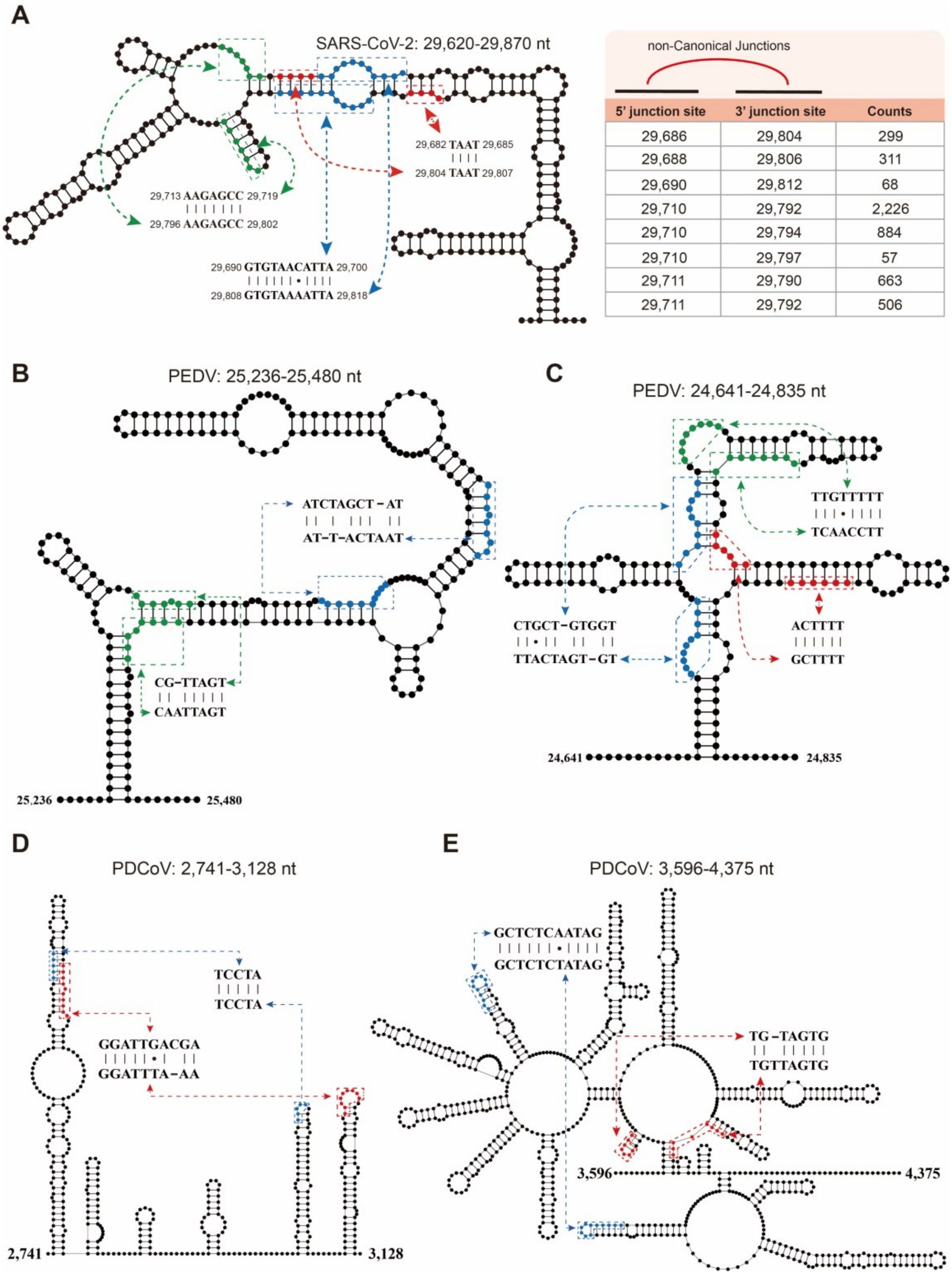

5' junction site	3' junction site	Counts
29,686	29,804	299
29,688	29,806	311
29,690	29,812	68
29,710	29,792	2,226
29,710	29,794	884
29,710	29,797	57
29,711	29,790	663
29,711	29,792	506

Appendix Figure S12. Stem-loops and TRS-like sequence pairs (highlighted in same color) in genomes of SARS-CoV-2 (A), PEDV (B-C) and PDCoV (D-E).

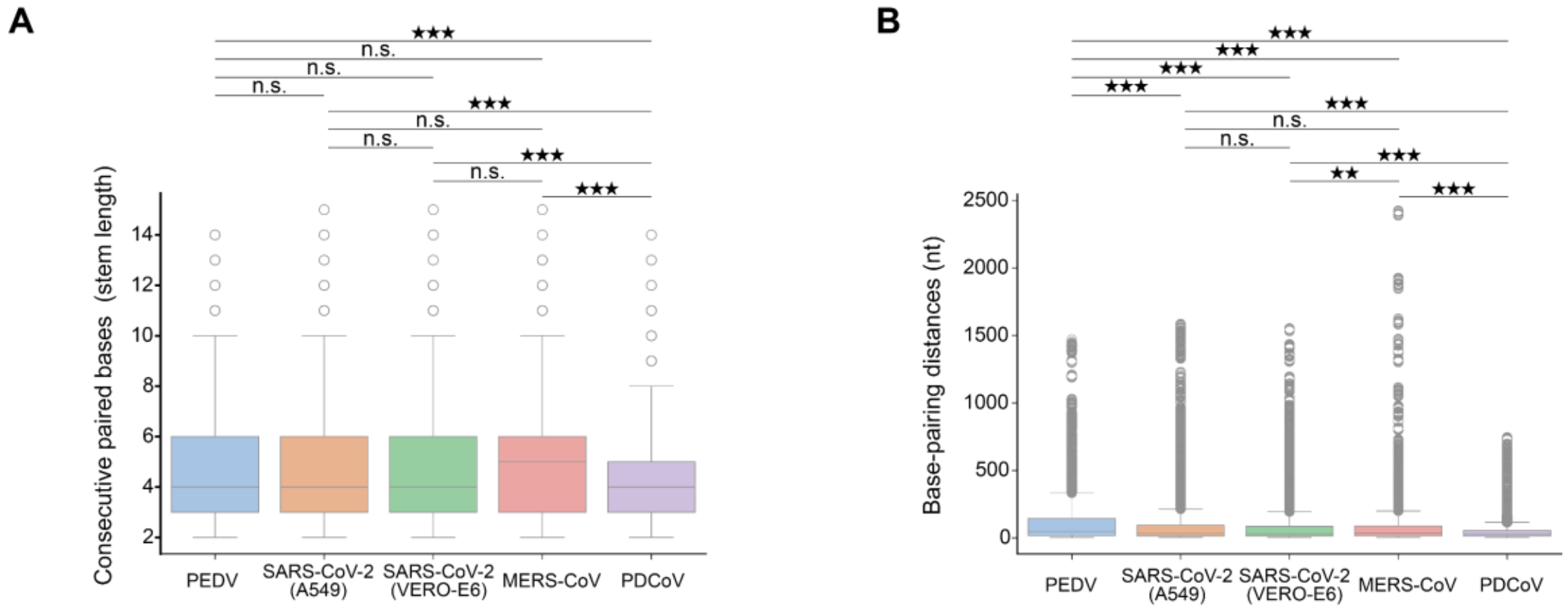

Appendix Figure S13. Comparison of RNA secondary structure properties across four coronaviruses.

A, Boxplots showing the distribution of consecutive paired bases (stem length) in predicted secondary structures for PEDV, SARS-CoV-2, MERS-CoV, and PDCoV. **B,** Boxplots showing the distribution of base-pairing distances (in nucleotides) for the same viruses. Statistical significance was assessed using a two-tailed Wilcoxon rank-sum test. ** for $p < 0.01$; *** for $p < 0.001$.

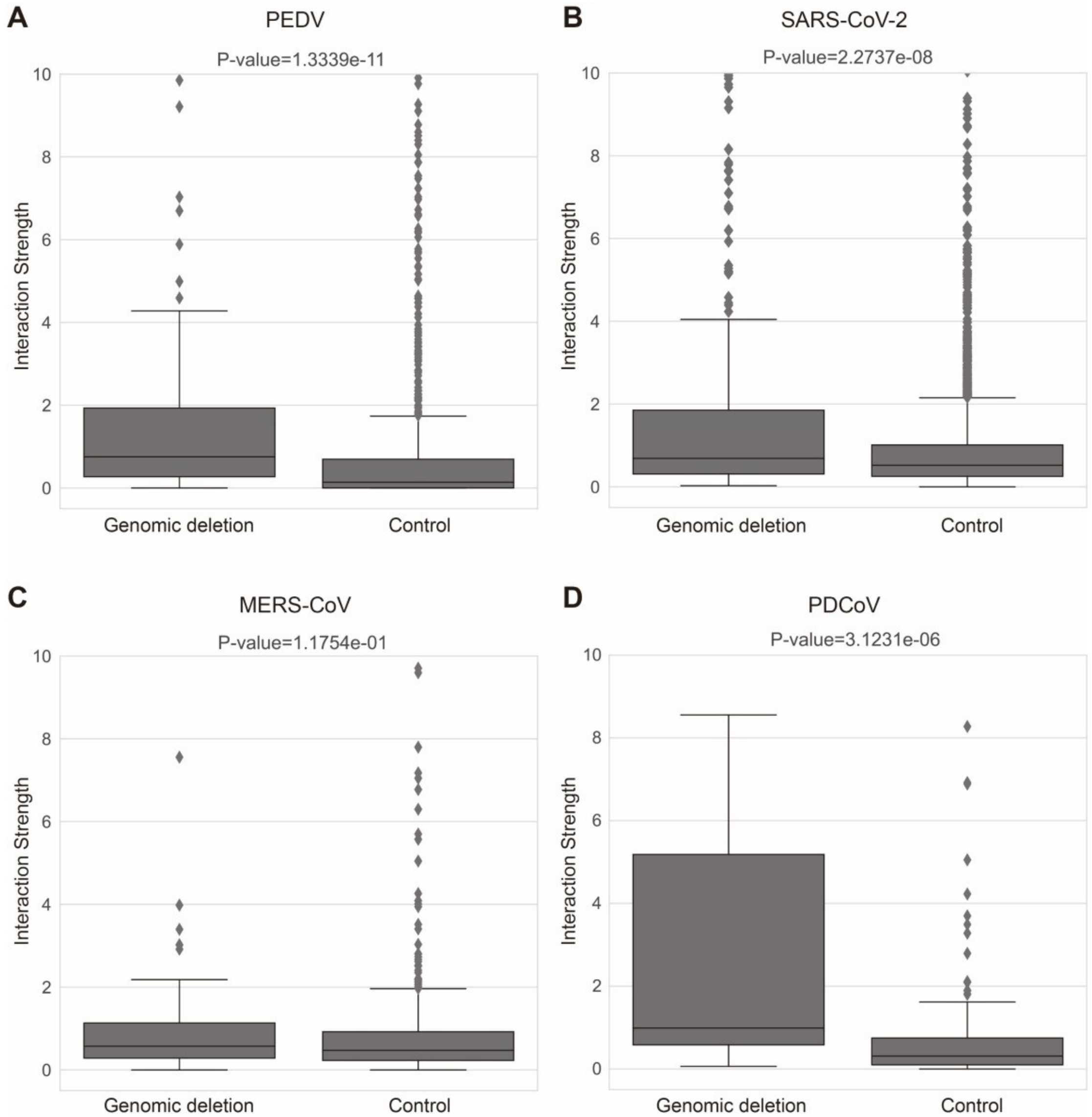

Appendix Figure S14. Interaction strength comparison for genomic deletions in different coronaviruses.

A-D, Boxplots depicting the RNA-RNA interaction strengths between ends of deletion regions for PEDV (A), SARS-CoV-2 (B), MERS-CoV (C) and PDCoV (D), compared with randomly paired genomic sites. Interaction strengths were determined by selecting the regions delineated between the two blue lines in Fig.5 A-D. The p-value of two-tailed Wilcoxon rank-sum test was calculated for each group.

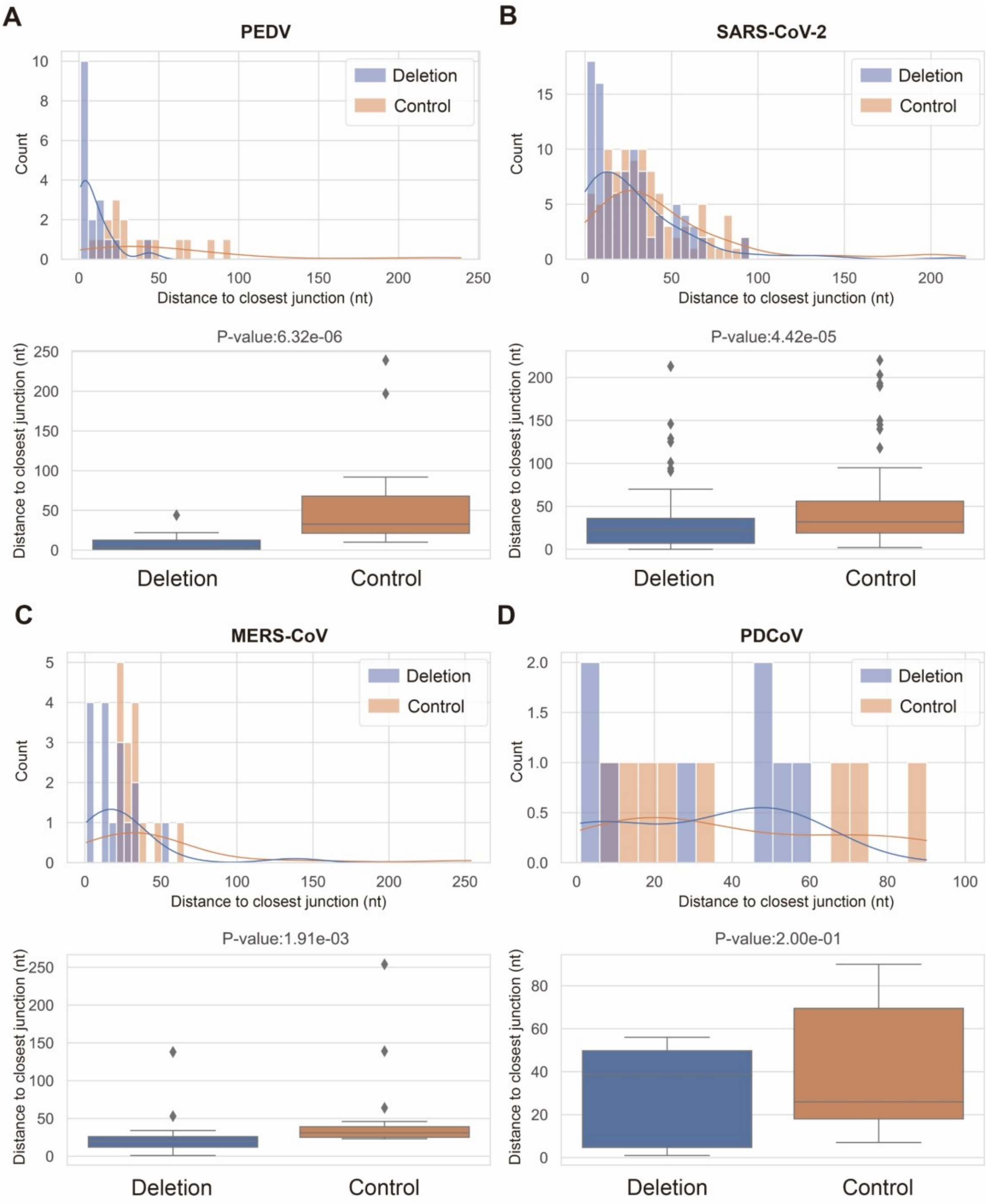

Appendix Figure S15. Distance between genomic deletion sites and non-canonical junction sites.

A, Histogram and boxplot of the distance distribution between deletion sites and short-range junction sites for PEDV (A), SARS-CoV-2 (B), MERS-CoV (C) and PDCoV (D). The P-value of two-tailed Wilcoxon rank-sum test was calculated for each group.

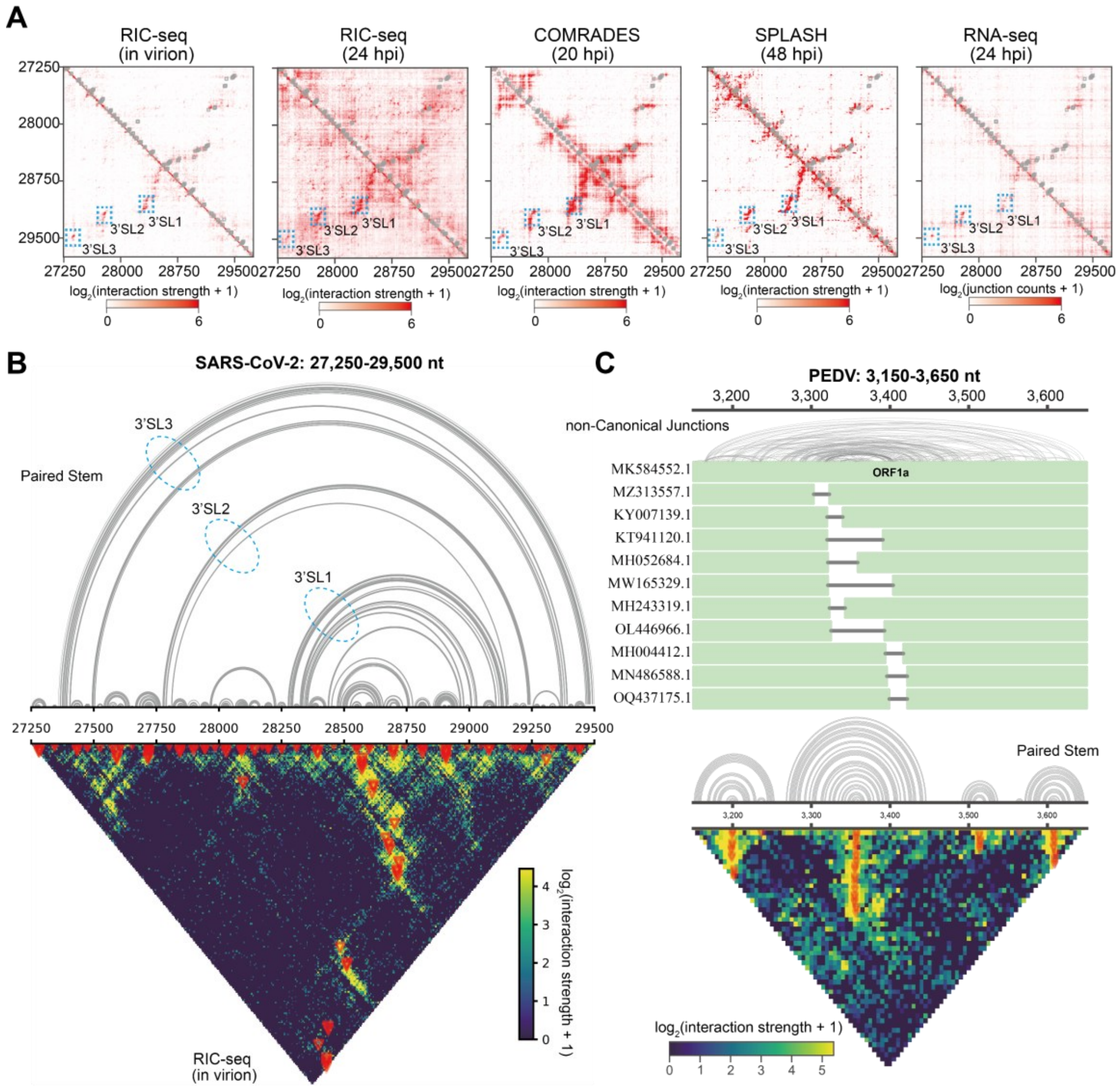

Appendix Figure S16. RNA secondary structures and non-canonical junctions of SARS-CoV-2 and PEDV.

A, RNA-RNA interaction heatmaps and short-range non-canonical junctions within the 3′SL1-3 region of SARS-CoV-2. Five subpanels are shown. The first four display interaction heatmaps from RIC-seq (Vero, in virion), RIC-seq (Vero, 24 hpi), COMRADES (Vero, 20 hpi), and SPLASH (Vero E6, 48 hpi). The fifth subpanel shows RNA-seq data (Vero, 24 hpi), where grey boxes denote short-range non-canonical junctions. Grey boxes outline the three stem-loops, which correspond to the predicted base-pairing interactions shown as grey dots in Fig. 5G–H. **B,** Base pairs within 3′SL1-3 predicted from RNA-RNA interactions captured by RIC-seq (Vero, in virion). **C**, A stem-loop region in the PEDV genome (MK584552.1: 3,150-3,650 nt) that contains 10 genomic deletions and abundant non-canonical junctions. The upper part shows genome structures with arcs above the genome indicating non-canonical junctions detected by RNA-seq. The lower part displays an RNA-RNA interaction heatmap with arcs indicating base pairs predicted from the interactions. Above the genome structures, arcs show predicted base pairs derived from the RNA secondary structure of this region.

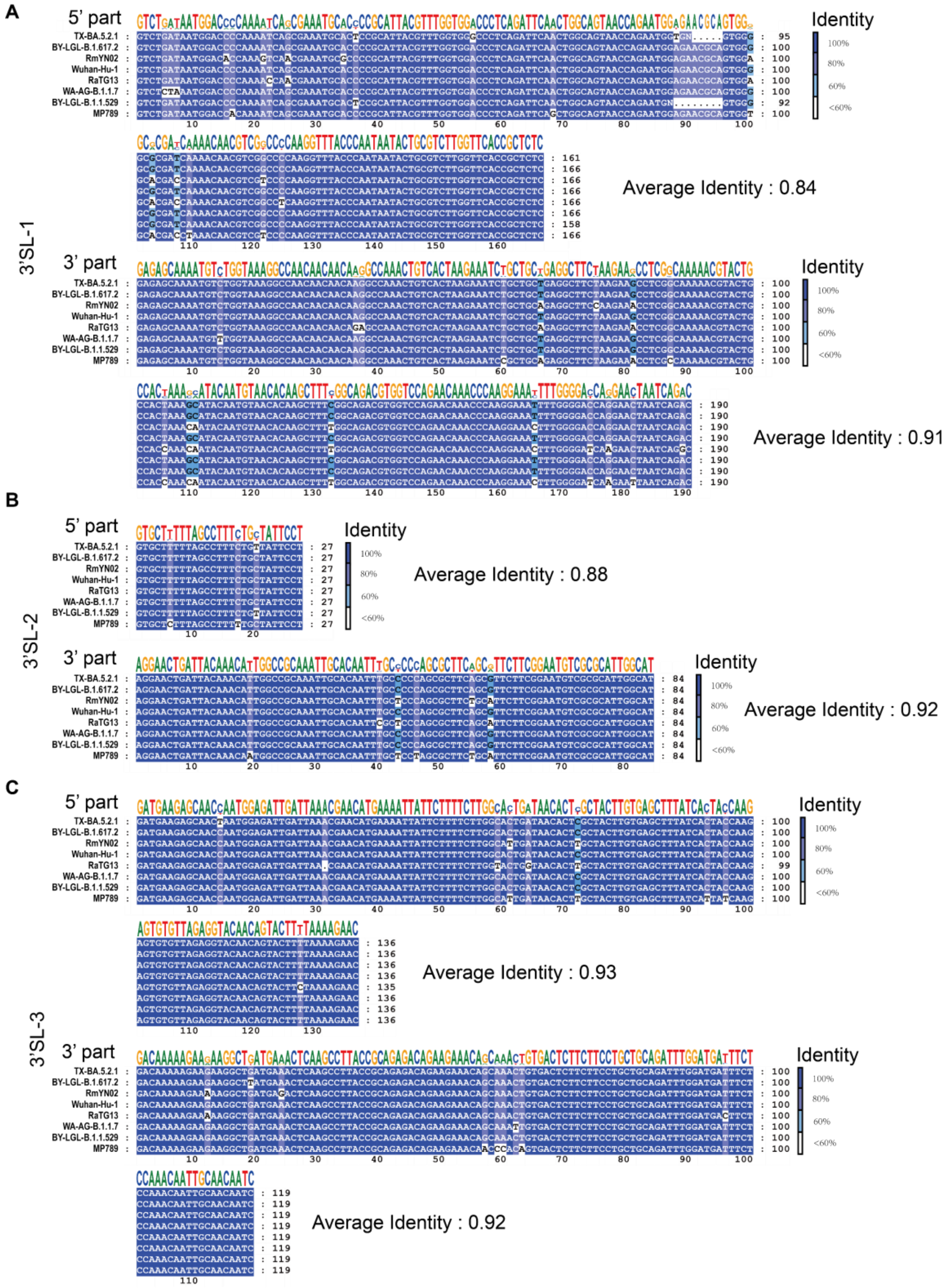

Appendix Figure S 17. Sequence identity in the 3′ and 5′ parts of 3′SL1 (A), 3′SL2 (B), and 3′SL3 (C) across SARS-CoV-2 related strain genomes.

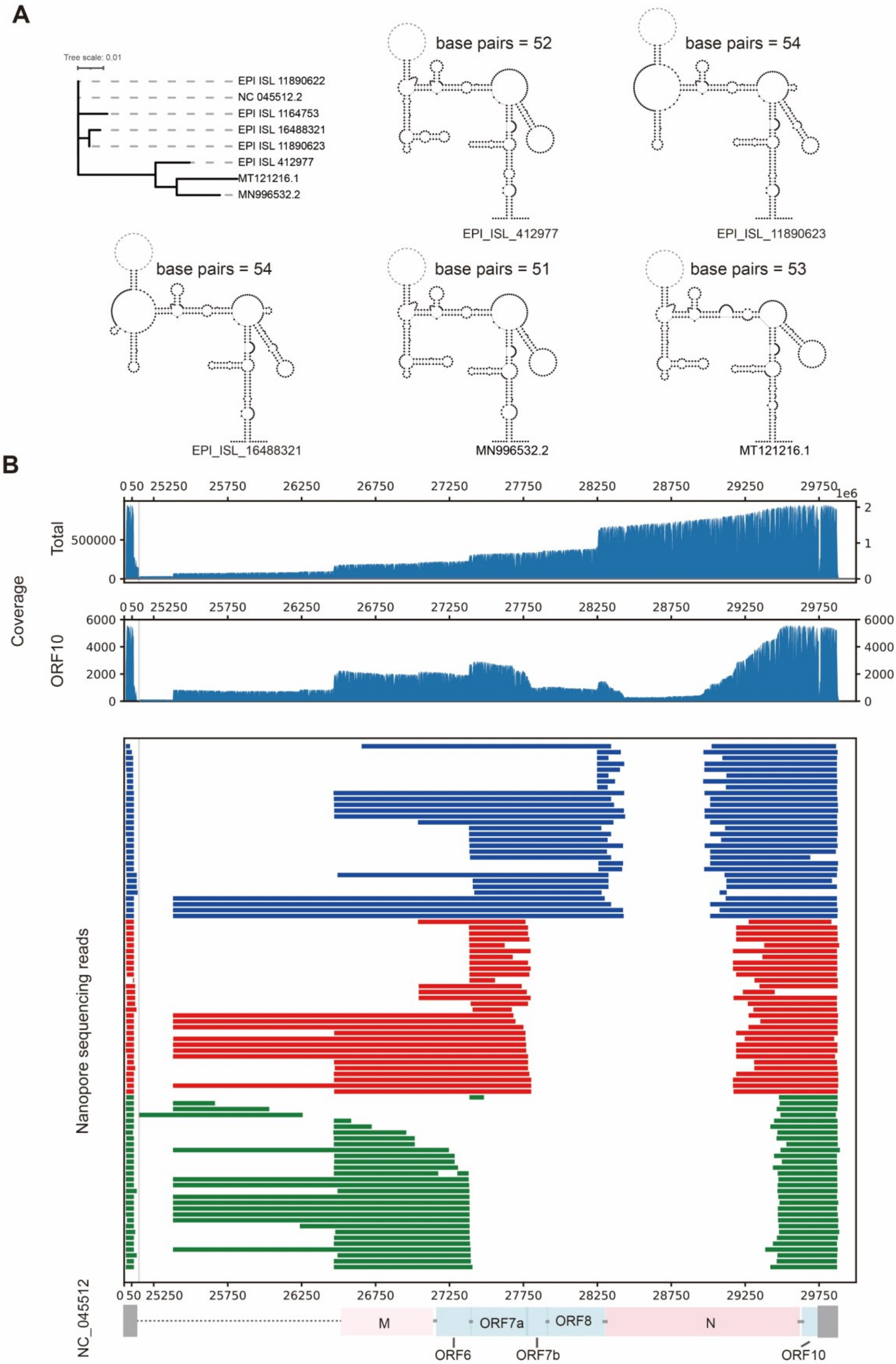

Appendix Figure S 18. Stable 3′SL1 and non-canonical ORF10 sgRNAs.

A, Phylogenetic tree of SARS-CoV-2 related strain genomes (upper left). Secondary structure of 3′SL1 predicted in the genome of SARS-CoV-2 related strains (lower right). **B**, Nanopore sequencing reads of ORF10 sgRNAs with double junctions.

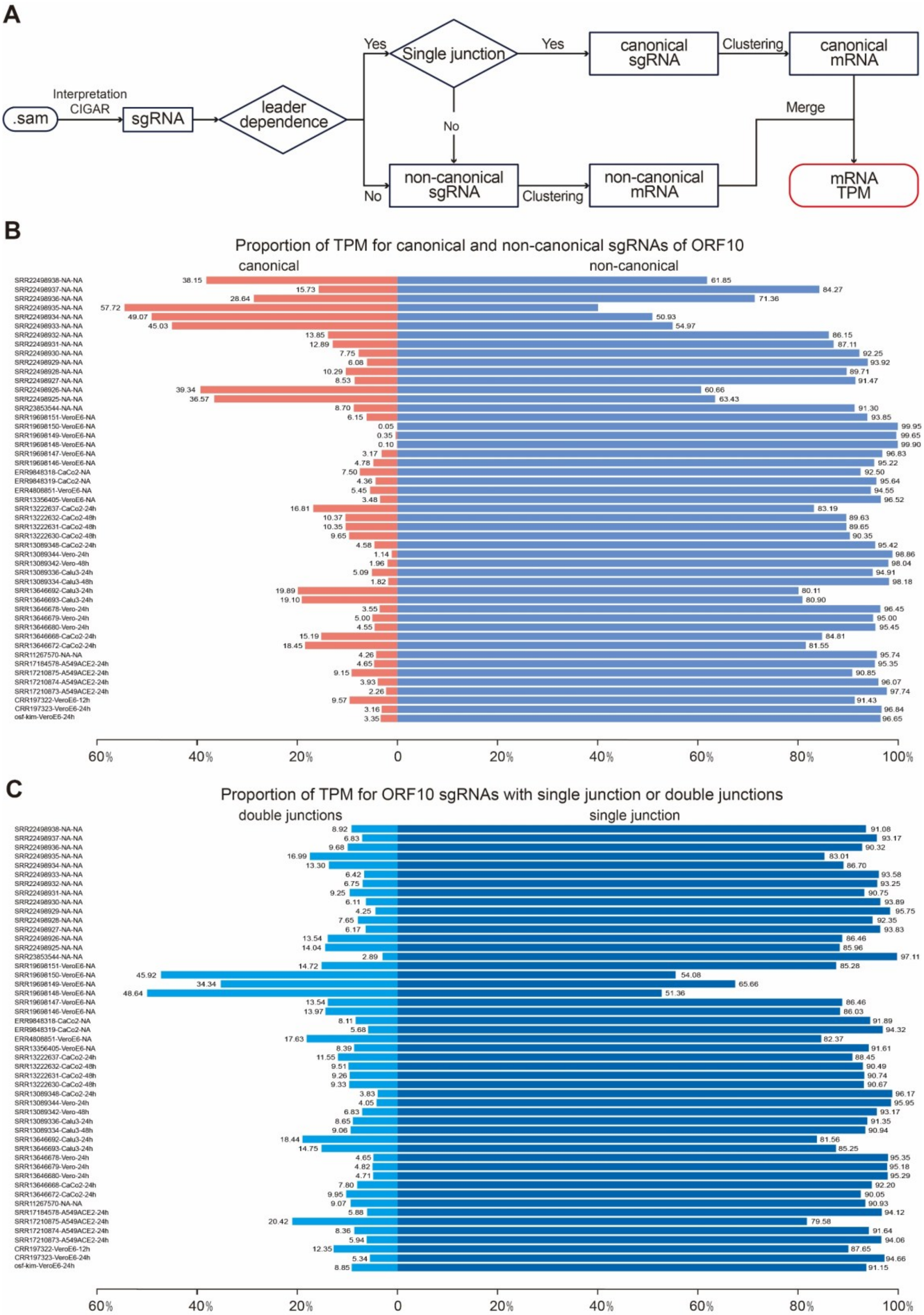

Appendix Figure S 19. Transcript levels of ORF10 sgRNAs calculated using Corona-mRNA. **A**, Workflow of Corona-mRNA. **B**, Proportion of transcripts per million (TPM) for canonical and non-canonical sgRNAs of ORF10 in each nanopore sample. **C**, Proportion of TPM for ORF10 sgRNAs with single or double junctions in each nanopore sample.

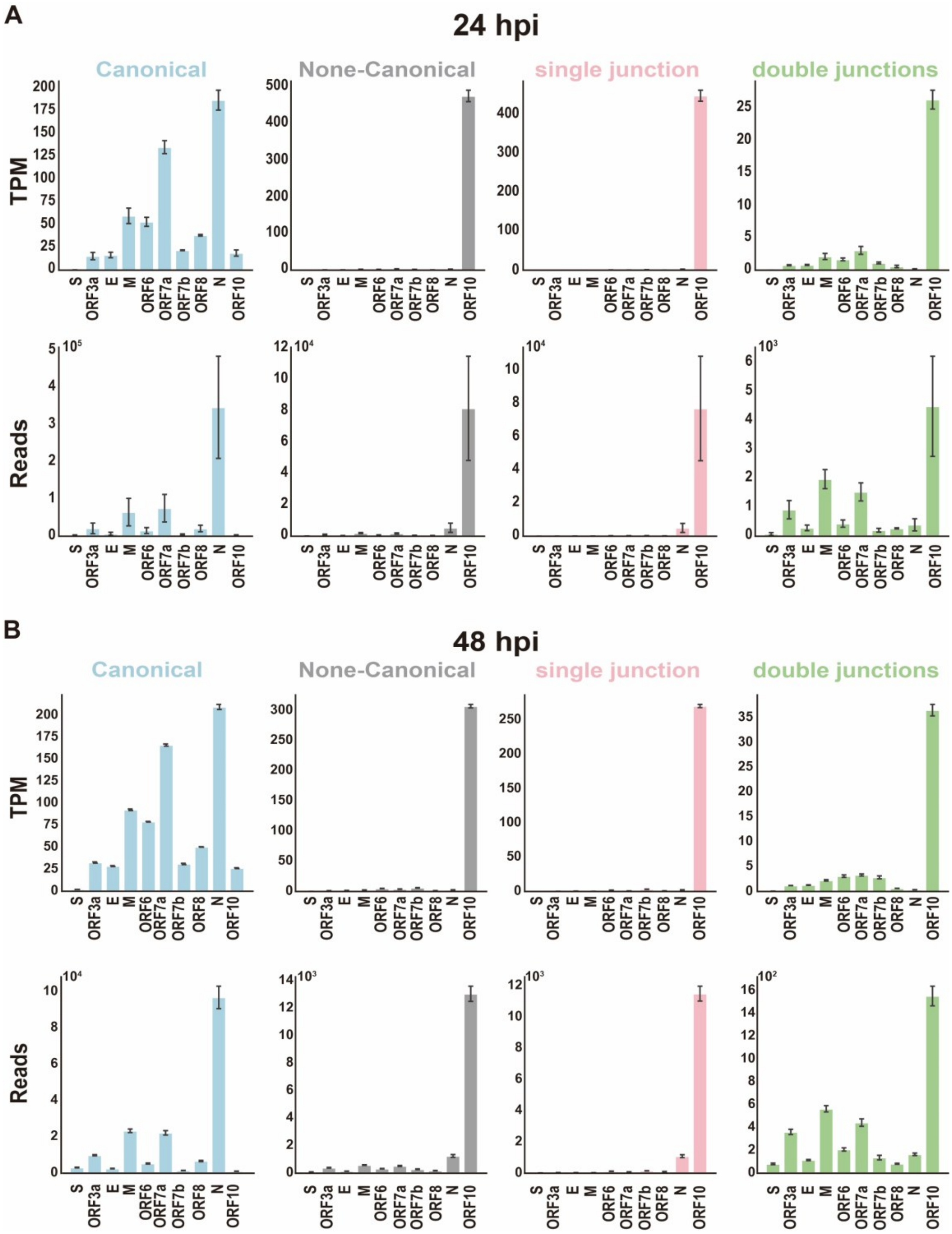

Appendix Figure S20. Transcription levels of individual genes in Vero cells at different hpi with SARS-CoV-2.

A-B, TPM and counts of reads for each gene which is transcribed through canonical and non-canonical junction at 24 hpi (A) and 48 hpi (B). Bars represent the mean proportion across all samples within each ORF; error bars indicate standard deviation.

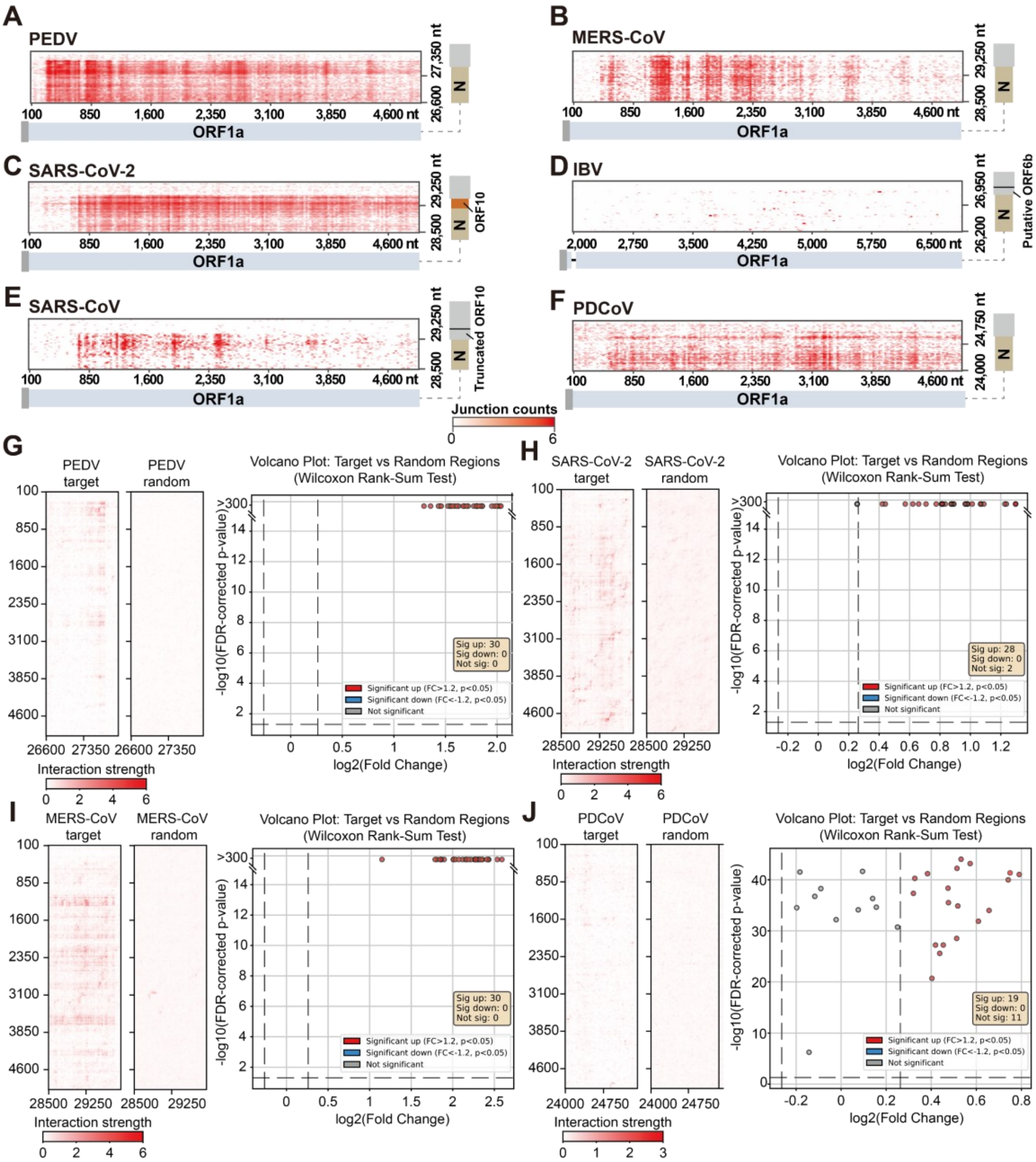

Appendix Figure S21. Non-canonical ORF1a-N junctions and RNA-RNA interactions across coronaviruses.

A-F, Heatmap of junction counts between ORF1a and N based on RNA-seq data and a schematic representation of sgRNA$_{N\text{-}ORF1a}$ are shown for PEDV (A), MERS-CoV (B), SARS-CoV-2 (C), and IBV (D), SARS-COV (E), PDCoV (F). **G-J**, Heatmap of RNA-RNA interactions between ORF1a and N based on RNA structurome data for PEDV (G), SARS-CoV (H), MERS-CoV (I), and PDCoV (J). In each panel, the left heatmap shows observed interaction frequencies, the middle heatmap shows the average of 100 random control regions matched in length and distance distribution, and the right volcano plot displays log2(fold change) versus –log10(FDR-corrected p-value). For PDCoV, due to its smaller genome, only 30 non-overlapping control regions could be selected.

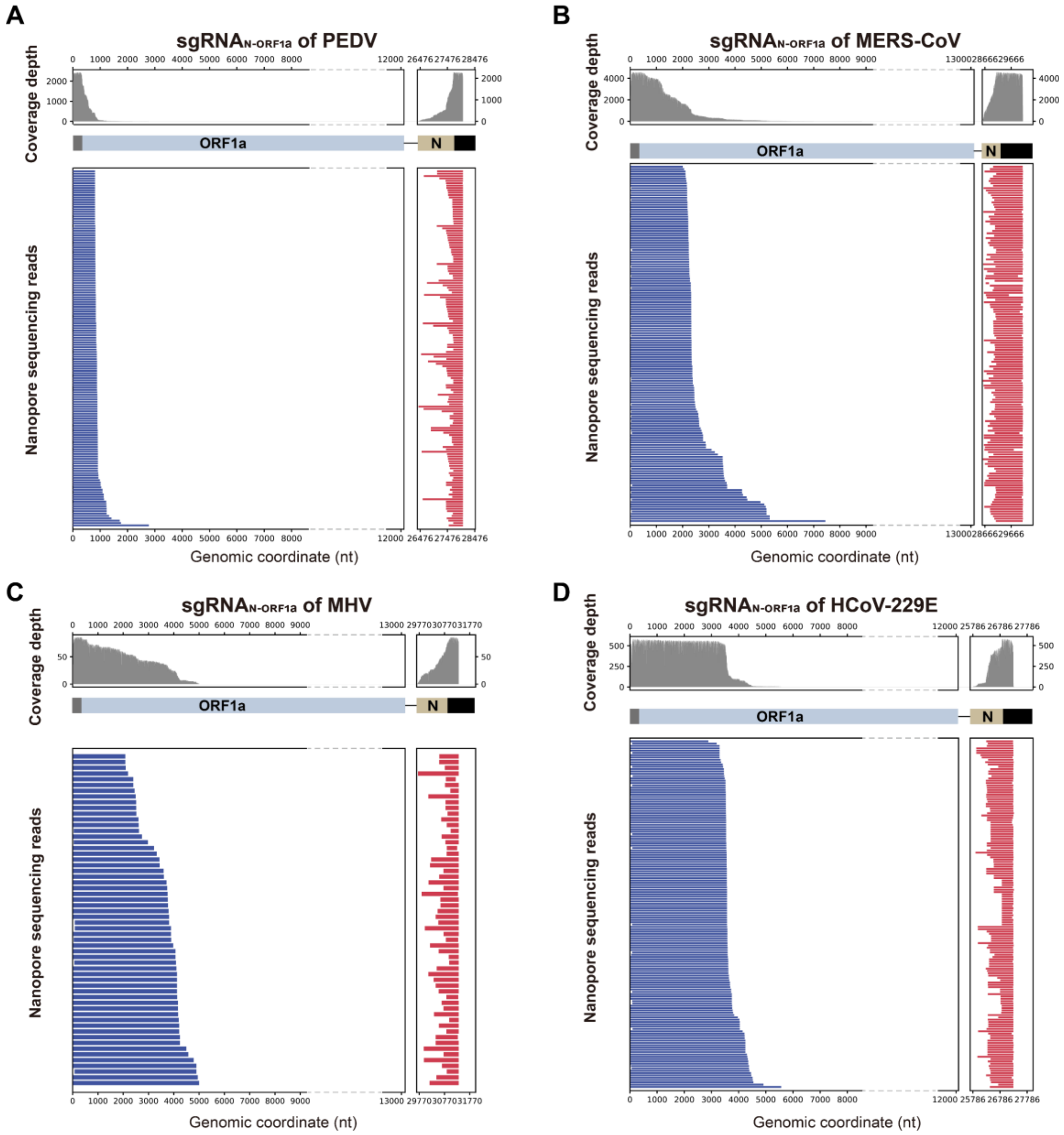

Appendix Figure S22. Nanopore sequencing validation of $sgRNA_{N\text{-}ORF1a}$ in multiple coronaviruses.

A-D, Detection of $sgRNA_{N\text{-}ORF1a}$ in PEDV (A), MERS-CoV (B), MHV (C), and HCoV-229E (D) by nanopore sequencing. For each virus, the figure is divided into left and right panels, corresponding to the 5′ UTR-ORF1a region (left) and the N-3′ UTR region (right). Each panel contains three tracks from top to bottom: genomic coverage (grey line), genome structure annotation (5′ UTR in grey, 3′ UTR in black; coding regions labeled), and individual nanopore reads. Each full-length read spans the junction between ORF1a and N; the portion aligning to the 5′ UTR-ORF1a region is shown in blue (left panel), and the portion aligning to the N-3′ UTR region is shown in red (right panel). The two colors together represent a single contiguous sgRNA molecule.

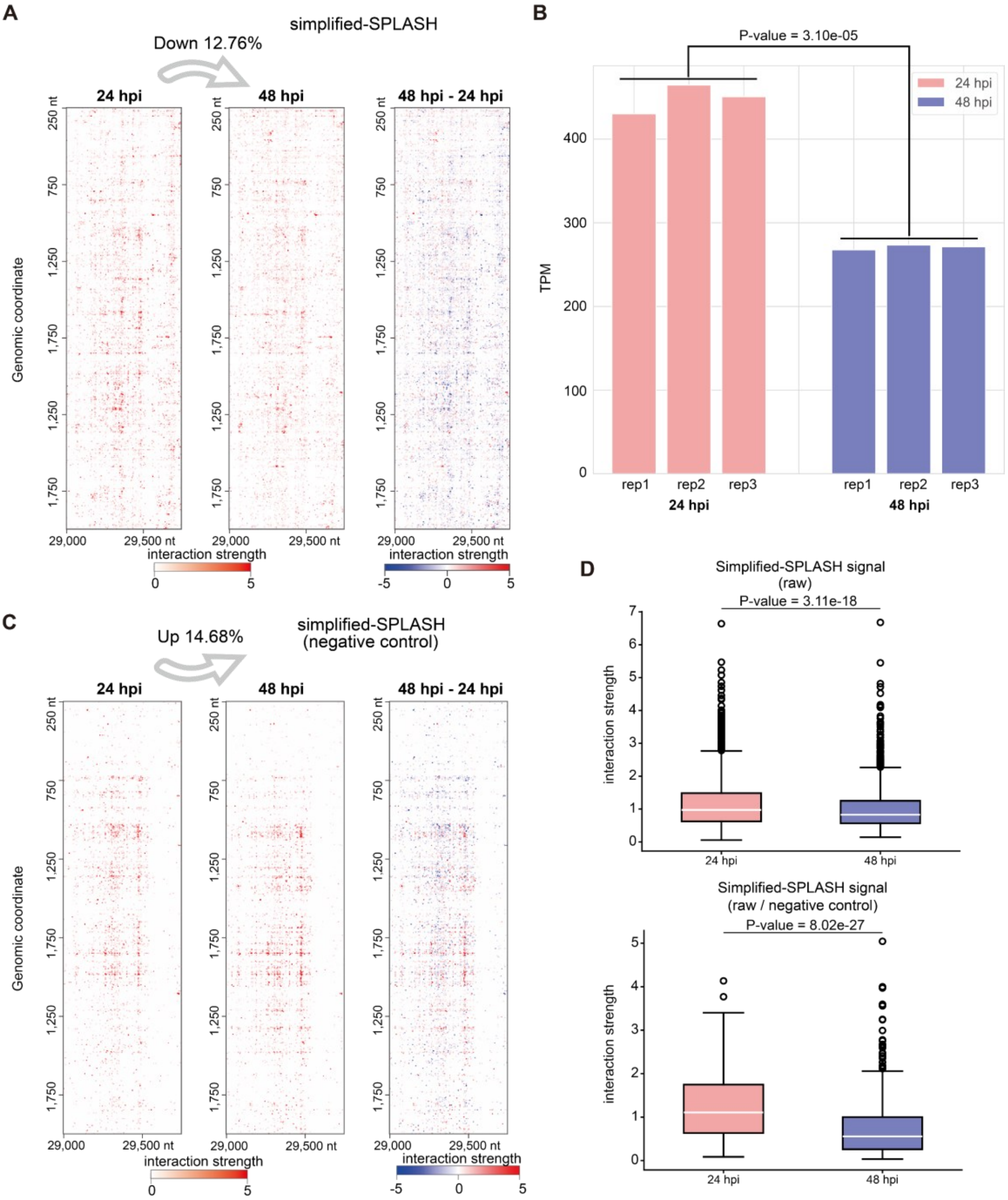

Appendix Figure S23. Correlation of junction counts with the RNA-RNA interaction strength.

A, Alterations in RNA-RNA interactions related to ORF10 $sgRNA_{N\text{-}ORF1a}$ at 24 hpi and at 48 hpi (simplified-SPLASH). **B**, Alterations in TPM of ORF10 $sgRNA_{N\text{-}ORF1a}$ at 24 hpi and at 48 hpi. The p-value of one-tailed t-test was calculated. **C**, Alterations in RNA-RNA interactions related to ORF10 $sgRNA_{N\text{-}ORF1a}$ at 24 hpi and at 48 hpi (Negative control of simplified-SPLASH). **D**, Boxplots comparing observed interaction counts from simplified-SPLASH data (with RNA ligase) at 24 hpi and 48 hpi (upper panel), and the ratio of these counts to the negative control (without RNA ligase) at the two time points (lower panel). P values were calculated using two-tailed Wilcoxon rank-sum test.

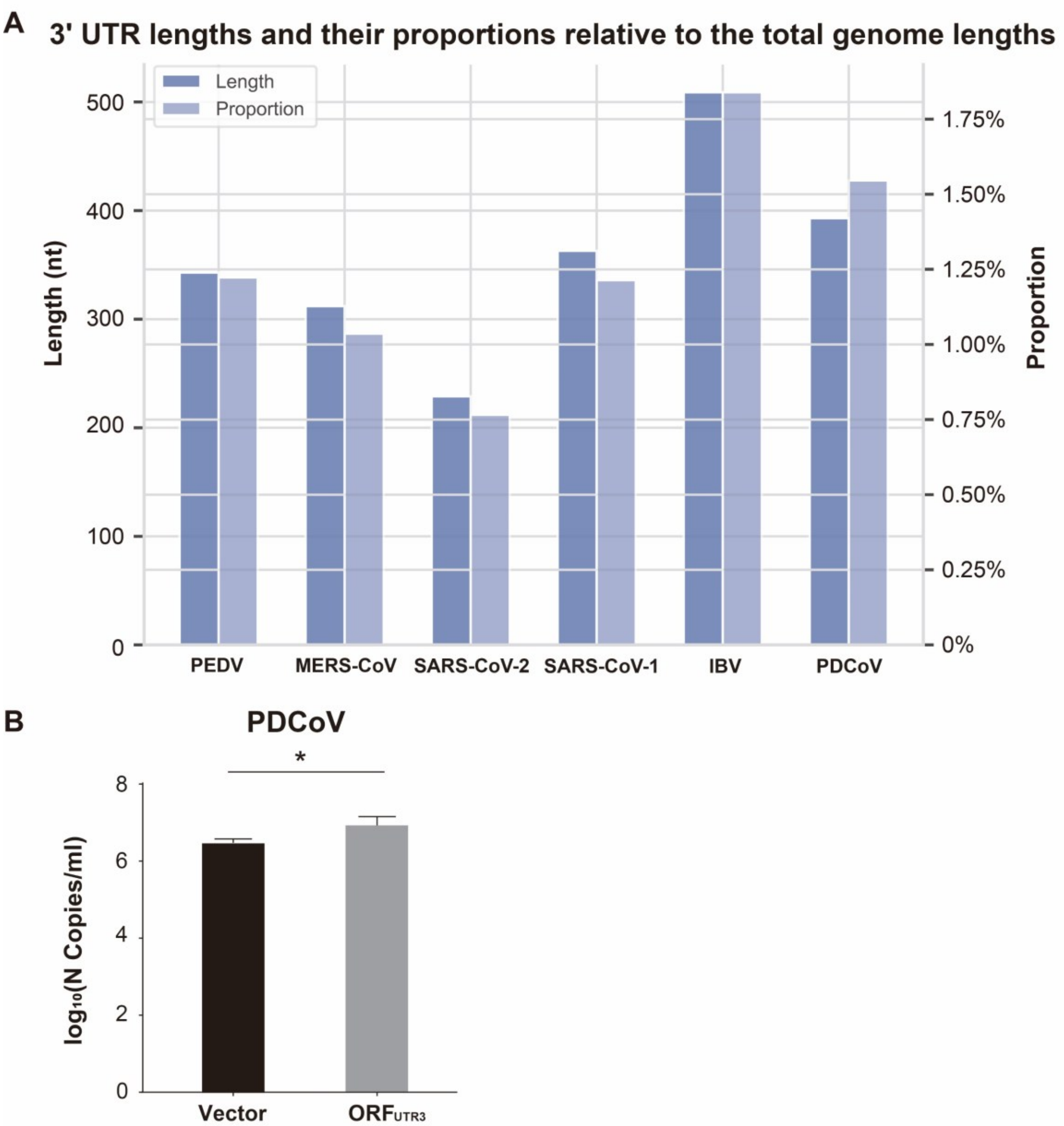

Appendix Figure S24. ORF$_{UTR3}$ in coronaviruses.

A, 3′ UTR lengths and their proportions relative to the total genome lengths among different coronaviruses. **B**, LLC-PK1 cells were transfected with the ORF$_{UTR3}$ plasmid or empty vector and were then infected with PDCoV. The transcription levels N gene of PDCoV were monitored by qRT–PCR. Data are presented as mean ± standard deviation from three independent biological replicates (n=3). Statistical significance was assessed using two-tailed t-test comparing ORFUTR3-expressing cells to empty vector controls (P-value = 0.040).

Appendix TABLE S1. Proportion of non-canonical junctions at different genomic distances

Virus	Proportion of short-range	Proportion of mid-range	Proportion of long-range	Division distance
SARS-CoV-2	22.31%	5.48%	72.21%	2,500 nt; 20,000 nt
MERS-CoV	4.43%	4.26%	91.31%	1,200 nt; 25,000 nt
PEDV	5.39%	2.31%	92.30%	1,700 nt; 22,000 nt
PDCoV	48.64%	7.49%	43.86%	2,100 nt; 18,750 nt

Appendix TABLE S2. Deletions obtained based on multiple sequence alignment

Virus	Number of genomes	Number of deletions	Number of deletions close to stem-loops	Proportion
SARS-CoV-2	1,110	105	31	29.52%
PEDV	902	31	14	45.16%
MERS-CoV	657	17	4	23.53%
PDCoV	549	8	5	62.50%

Appendix TABLE S3. Locations of enriched genomic deletions in SARS-CoV-2 genome （NC_045512.2）

Reference	Location	Length	Region	Collection date	Area
EPI_ISL_414378	27,848-28,229	382	ORF7b-ORF8	2020-02-17	Singapore
EPI_ISL_450343	27,905-28,250	346	ORF8	2020-05-09	Bangladesh
EPI_ISL_832092	28,003-28,190	188	ORF8	2020-04	USA
EPI_ISL_426967	27,826-27,967	142	ORF7b-ORF8	2020-03-27	Australia
EPI_ISL_2138877	27,827-27,959	133	ORF7b-ORF8	2021-05-03	USA
EPI_ISL_2186398	27,808-27,897	90	ORF7b-ORF8	2021-04-19	USA
EPI_ISL_452497	27,904-27,965	62	ORF8	2020-03-19	Spain
EPI_ISL_445213	27,438-27,657	220	ORF7a	2020-04-28	Bangladesh
EPI_ISL_1866778	27,504-27,689	186	ORF7a	2021-04-05	Denmark
EPI_ISL_1443278	27,519-27,689	171	ORF7a	2021-03-18	USA
EPI_ISL_2470817	27,519-27,725	207	ORF7a	2021-05-26	Germany
EPI_ISL_806843	27,531-27,684	154	ORF7a	2020-12-08	USA
EPI_ISL_2174719	27,536-27,651	116	ORF7a	2021-05-03	Denmark
EPI_ISL_1235896	27,555-27,646	92	ORF7a	2021-03-07	USA
EPI_ISL_2284503	27,563-27,640	78	ORF7a	2021-04-29	Netherlands
EPI_ISL_2401741	27,621-27,670	50	ORF7a	2021-05-22	Luxembourg

Appendix TABLE S4 Locations of enriched genomic deletions in PEDV genome (MK584552)

Reference	Location	Length	Region	Collection date	Area
MZ313557.1	3,303-3,323	21	ORF1a	2016-04-01	Poland
KY007139.1	3,320-3,340	21	ORF1a	2015-05-11	China
KT941120.1	3,320-3,391	72	ORF1a	2014-10-15	Viet Nam
MH052684.1	3,321-3,359	39	ORF1a	2017-12	South Korea
MW165329.1	3,321-3,404	84	ORF1a	2013-07-01	Taiwan
MH243319.1	3,323-3,343	21	ORF1a	2018-05	South Korea
OL446966.1	3,325-3,393	69	ORF1a	2020	China
MH004412.1	3,394-3,417	24	ORF1a	2016-07-19	Mexico
MN486588.1	3,396-3,422	27	ORF1a	2016-02	China
OQ437175.1	3,399-3,422	24	ORF1a	2021-09-21	China
