# Supplementary material for: Cross-genus analysis reveals architecturally programmed sgRNA synthesis patterns in coronaviruses
Source: Mol Syst Biol. 2026 Jul 30;22(11):1771–88. doi: 10.1038/s44320-026-00239-0 (PMC13728164; doi:10.1038/s44320-026-00239-0)
Supplement: Supplementary file 2 [file 44320_2026_239_MOESM2_ESM.pdf]

molecular systems biology

Cross-Genus Analysis Reveals Architecturally Programmed sgRNA Synthesis Patterns in Coronaviruses

Zi Wen, Lei Chen, Dehua Luo, Ju Sun, Liangrong Guo, Yingxiang Deng, Zhiyuan Huang, Yuxiang Wang, Ke Pan, Fan Wang, Shaobo Xiao, Li Li, and Dengguo Wei

Corresponding author(s): Dengguo Wei (dgwei@mail.hzau.edu.cn) , Shaobo Xiao (vet@mail.hzau.edu.cn), Fan Wang (fwang@mail.ccnu.edu.cn), Li Li (li.li@mail.hzau.edu.cn)

Review Timeline:

Submission Date:	14th Oct 25
Editorial Decision:	14th Jan 26
Revision Received:	31st May 26
Editorial Decision:	26th Jun 26
Revision Received:	11th Jul 26
Accepted:	14th Jul 26

Editor: Poonam Bheda

Transaction Report:

(Note: With the exception of the correction of typographical or spelling errors that could be a source of ambiguity, letters and reports are not edited. Depending on transfer agreements, referee reports obtained elsewhere may or may not be included in this compilation. Referee reports are anonymous unless the Referee chooses to sign their reports.)

14th Jan 2026

Manuscript Number: MSB-2025-13406-T
Title: Cross-Genus Analysis Reveals Architecturally Programmed sgRNA Synthesis Patterns in Coronaviruses

Dear Prof. Wei,

Thank you for the submission of your manuscript to Molecular Systems Biology. We have now received feedback from the two reviewers who agreed to evaluate your manuscript. As you will see from the reports below, the referees acknowledge the interest of the study and are overall supportive of your work; however they also comment on multiple aspects of the manuscript that should be strengthened in a revision.

I think that the recommendations of the reviewers are rather clear and I therefore do not see the need to repeat the comments listed below. All issues raised would need to be satisfactorily addressed. Please let me know in case you would like to discuss in further detail any of the any of the reviewer comments or your proposed revisions, I would be happy to schedule a call.

When submitting your revised manuscript, please carefully review the instructions that follow below. We perform an initial quality control of all revised manuscripts before re-review; failure to include requested items will delay the evaluation of your revision.

We require:

1) A .docx formatted version of the manuscript text (including legends for main figures, EV figures and tables). Please make sure that the changes are highlighted to be clearly visible. Alternatively you may choose to submit your manuscript as a LaTeX file.

2) Individual production quality figure files as .eps, .tif, .jpg (one file per figure). For guidance, download the 'Figure Guide PDF' (https://media.springernature.com/original/springer-cms/rest/v1/content/27825798/data/v1).

3) At EMBO Press we ask authors to provide source data for the main figures. Our source data coordinator will contact you to discuss which figure panels we would need source data for and will also provide you with helpful tips on how to upload and organize the files.

4) A .docx formatted letter INCLUDING the reviewers' reports and your detailed point-by-point responses to their comments. As part of the EMBO Press transparent editorial process, the point-by-point response is part of the Peer Review File (PRF), which will be published alongside your paper.

5) A complete author checklist, which you can download from our author guidelines (https://link.springer.com/journal/44320/submission-guidelines#cms). Please insert information in the checklist that is also reflected in the manuscript. The completed author checklist will also be part of the PRF.

6) Please note that all corresponding authors are required to supply an ORCID ID for their name upon submission of a revised manuscript.

7) It is mandatory to include a 'Data Availability' section after the Materials and Methods. Before submitting your revision, primary datasets produced in this study need to be deposited in an appropriate public database, and the accession numbers and database listed under 'Data Availability'. Please remember to provide a reviewer password if the datasets are not yet public (see https://link.springer.com/partners/embo-press/editorial-policies#Data%20availability%20statement).

In case you have no data that requires deposition in a public database, please state so in this section as follows: "This study includes no data deposited in external repositories". Note that the Data Availability Section is restricted to new primary data that are part of this study.

8) All Materials and Methods need to be described in the main text using our 'Structured Methods' format, which is required for all research articles. According to this format, the Methods section includes a Reagents and Tools Table (listing key reagents, experimental models, software and relevant equipment and including their sources and relevant identifiers) followed by a Methods and Protocols section describing the methods using a step-by-step protocol format. The aim is to facilitate adoption of the methodologies across labs. Please upload the Reagents and Tools table as a separate document when submitting your revised manuscript. More information on how to adhere to this format as well as a downloadable template (.docx) for the Reagents and Tools Table can be found in our author guidelines: https://link.springer.com/journal/44320/submission-guidelines#cms

9) For data quantification: please specify the name of the statistical test used to generate error bars and p-values, the number

(n) of independent experiments (specify technical or biological replicates) underlying each data point and the test used to calculate p-values in each figure legend. The figure legends should contain a basic description of n, p-values and the test applied. Graphs must include a description of the bars and the error bars (s.d., s.e.m.). Please provide exact p-values (in either the figure or figure legend).

10) Our journal encourages inclusion of *data citations in the reference list* to directly cite datasets that were re-used and obtained from public databases. Data citations in the article text are distinct from normal bibliographical citations and should directly link to the database records from which the data can be accessed. In the main text, data citations are formatted as follows: "Data ref: Smith et al, 2001" or "Data ref: NCBI Sequence Read Archive PRJNA342805, 2017". In the Reference list, data citations must be labeled with "[DATASET]". A data reference must provide the database name, accession number/identifiers and a resolvable link to the landing page from which the data can be accessed at the end of the reference. Further instructions are available at .

11) We replaced Supplementary Information with Expanded View (EV) Figures and Tables that are collapsible/expandable online. EV Figures should be cited as 'Figure EV1, Figure EV2" etc... in the text and their respective legends should be included in the main text after the legends of regular figures.
- For the figures that you do NOT wish to display as Expanded View figures, they should be bundled together with their legends in a single PDF file called *Appendix*, which should start with a short Table of Content. Appendix figures should be referred to in the main text as: "Appendix Figure S1, Appendix Figure S2" etc.
- Additional Tables/Datasets should be labeled and referred to as Table EV1, Dataset EV1, etc. Legends should be provided in a separate tab in case of .xls files. Alternatively, the legend can be supplied as a separate text file (README) and zipped together with the Table/Dataset file.
See detailed instructions here:
https://link.springer.com/journal/44320/submission-guidelines#cms

12) Author contributions: CRediT has replaced the traditional author contributions section because it offers a systematic machine-readable author contributions format that allows for more effective research assessment. Please remove the Authors Contributions from the manuscript and use the free text boxes beneath each contributing author's name in our system to add specific details on the author's contribution. More information is available in our guide to authors.

13) Disclosure statement and competing interests: We updated our journal's competing interests policy in January 2022 and request authors to consider both actual and perceived competing interests. Please review the policy https://link.springer.com/partners/embo-press/editorial-policies#Competing%20interest%20disclosures and update your competing interests if necessary.

14) Every published paper now includes a 'Synopsis' to further enhance discoverability. Synopses are displayed on the journal webpage and are freely accessible to all readers. They include a short stand first (maximum of 300 characters, including space) as well as 2-5 one-sentences bullet points that summarizes the paper. Please write the bullet points to summarize the key NEW findings. They should be designed to be complementary to the abstract - i.e. not repeat the same text. We encourage inclusion of key acronyms and quantitative information (maximum of 30 words / bullet point). Please use the passive voice. Please attach these in a separate file or send them by email, we will incorporate them accordingly.

Please also suggest a striking image or visual abstract to illustrate your article as a PNG file 550 px wide x 300-600 px high.

Please note that these would be the final versions and changes during proofing are usually not allowed.

15) As part of the EMBO Publications transparent editorial process initiative (see our policy here: https://link.springer.com/partners/embo-press/editorial-policies#Peer%20review), Molecular Systems Biology will publish online a Peer Review File (PRF) to accompany accepted manuscripts.
In the event of acceptance, this file will be published in conjunction with your paper and will include the anonymous referee reports, your point-by-point response and all pertinent correspondence relating to the manuscript. Let us know whether you agree with the publication of the PRF and as here, if you want to remove or not any figures from it prior to publication.
Please note that the Author checklist will be published at the end of the PRF.

Molecular Systems Biology has a "scooping protection" policy, whereby similar findings that are published by others during review or revision are not a criterion for rejection. Should you decide to submit a revised version, I do ask that you get in touch after three months if you have not completed it, to update us on the status.

Yours sincerely,

Poonam Bheda

Poonam Bheda, PhD
Senior Scientific Editor
Molecular Systems Biology

Reviewer #1:

Summary:
The manuscript by Wen et al. presents an extensive analysis of coronavirus RNA structural and expression data, integrating RNA-seq and RNA-RNA interactome data from four coronavirus genera, including curated and newly generated data. The study also includes cross-genus genome comparisons and examines the effect of a putative Porcine Deltacoronavirus ORF on immune response gene expression. The authors conclude that structural elements in the coronavirus genome facilitate deletions and the formation of both canonical and non-canonical sgRNA, and suggest this is an adaptive mechanism.

General remarks:
This main novelty of this work lies in the comparative analysis across different genera, highlighting conserved features. This and other results in the manuscript will be valuable for RNA virus research. However, some speculative claims regarding causality between RNA structure and sgRNA junction formation are inadequately supported. Additionally, the central analysis pipeline, key terms, and certain additional analyses are poorly described, and some visualizations and analyses lack sufficient statistical rigor and clarity. Overall, the manuscript presents interesting findings but requires a more critical approach to its claims and methods. Addressing these points will significantly enhance its clarity and impact.

Major points:
- The claim of increased interaction as a result of parallel base-pairing is surprising, given its rarity (PMID: 28771935). The authors should either strengthen their evidence for this claim or consider more plausible mechanisms, such as RBP or negative strand RNA-mediated interactions (figures 3C, S6-9). The correlation observed in figure S9B should also be examined for clarity, as it appears to be largely driven by a single sgRNA (ORF N).
- The potential for detection of indirect RNA-RNA interactions should be addressed, as processes like discontinuous transcription can cause the observed proximity.
- Speculative Claims: Overreaching statements regarding non-canonical sgRNAs as adaptive elements driving genome plasticity (in the abstract, introduction line 103, discussion line 451) lack robust supporting data. The evidence for any evolutionary roles remains unclear, especially given the suggested low expression levels during infection (as opposed to the high levels in the overexpression experiment).
- The manuscript's analysis pipelines lacks a thorough description, both in the text and in the GitHub code, making it challenging to follow how specific analyses were conducted. Clear documentation would enhance reproducibility, and the current state does not allow reproducing the data.
- There is no discussion of the low relative abundance of non-canonical versus canonical junctions, which is crucial for interpreting the results, particularly regarding figure S21 where TPM calculated separately for each category is shown side by side.
- Figure 2B shows non-canonical junction relative abundance decreases with infection time and increases with virus passage, in the PEDV data created by the authors. Since this is a surprising and differs from previous results (e.g. PMID: 32906143, PMID: 33961822) and since this manuscript includes analysis of many RNA-seq data sets, including several timecourses, it would be helpful to perform a similar analysis on all available data to more carefully test this hypothesis. The data sets also include different cell types and drug treatments, which could also be incorporated into a more complete answer to the question of non-canonical sgRNA dynamics.
- Several key terms are inadequately defined:
o "non-canonical junctions", as used in this manuscript, is not clearly and consistently defined. Please clearly describe what consists as canonical or non-canonical junction, and what is excluded from these definitions. Is a junction between TRS-L and a non-TRS-B position considered canonical or non-canonical? Are these definitions consistent between the RNA-seq and nanopore pipeline analyses?
o The terms "divergent sequence pairs/orientations" and "directional RNA-RNA interactions".
o Line 167: do the numbers represent unique canonical junctions, or unique reads spanning canonical junctions?
o In figure 6 and S18-21 it is unclear how ORF10 sgRNA is identified in sequence data. What are canonical ORF10 sgRNA? This needs clarification especially since fig S21 shows an overwhelming majority of non-canonical sgRNAs are defined as ORF10 sgRNAs.
- Figure annotations and legends require more detail regarding experimental conditions, statistical tests, and subset clarifications.
- There is a lack of rigorous statistical analysis across the data sets, particularly concerning the robustness of results against a null hypothesis. Detailed randomization approaches should be reported to strengthen claims.
- Quality control for sequencing libraries is largely missing, both for newly generated libraries (other than replicate correlation)

and for the analysis itself, please provide QC analyses such as:
o % human vs virus reads
o % canonical vs non-canonical junctions
o Detection of virus-host RNA interactions
o Recapitulation of known structures (e.g. 5` and 3` UTR stem loops, TRS-L stem loops and others predicted from sequence and previous works) and junctions.

Minor points:
- The manuscript could benefit from streamlining the data presented in the supplements and enhancing the focus on the main findings.
- Figure legends need additional annotations:
o Include descriptions of infection/transfection time, MOI, statistical significance tests, and number of replicates in all relevant legends.
o Clarify whether all junctions or a subset is shown, and the criteria for any subsetting.
o Define arrows and dashed lines in S2A.
o Add annotations for highlighted elements in Fig 2C (red dots, starred area) and specify whether all or only non-canonical junctions are shown.
o Provide additional genome annotations for APA figures, including directionality with arrows and clarifications regarding the meaning of + signs in Fig 2A, especially the meaning of the double + signs between the 3rd and the 4th columns (M and N).
o Expand descriptions of sashimi plots in legends of figures 5E and S15-16.
o Explain the gray point labels in fig 5G-H and S15A, and highlight stem loops in S15A.
o Describe the different lines in fig 6B for improved clarity.
- TRS-like sequences: confirm if the entire genome was searched for TRS-like pairs and how regions I, II, and III were selected in Fig 2C. Include quantitative measures for TRS-like sequences and their associations with RNA-RNA interactions.
- Line 66: Reference 10 does not support the claim that non-canonical sgRNAs encode functional polypeptides, please adjust the sentence to more appropriately reflect the paper's results.
- Describe how the data sets were collected and clarify the reasons for excluding certain datasets. Include information on which data sets went into each analysis. This should be part of the main text and possibly figure 1.
- Clarify how Fig 2D and S2A account for size differences between viruses.
- Explain the inclusion of Fig S2B, as it appears to present the same data as S2C and 2A.
- The deposited PEDV RNA-seq includes two replicates, but not the data from the experiment in fig 2B.
- Provide a complete description of the APA method, clarifying how it was applied and how data deviates from null/chance results, and discuss the meaning of the cross-shaped patterns in APA data (e.g. in fig 4C).
- For all RNA structures shown, please show the predicted G from mFold for each selected structure as well as the lowest (most negative) G outputted of all of the potential structures predicted, to give a sense of the thermodynamic favorability of the parallel strand formation versus other structural ensembles.
- Please address the deviation of PDCoV from the results more directly. Is it a true difference from other viruses, a non-significant difference, or a result of the lower reproducibility of PDCoV data?
- Include a table with detected genomic deletions and their sizes. Could deletion size differences between viruses explain the different results in fig 5A-D?
- Line 334, please refer to fig S15A.
- Confirm if putative 3' UTR ORFs sgRNAs in other viruses are supported by the analyzed data.
- Include exact time of infection in library preparation methods, along with the number of cells and MOI.
- A reverse transcription step is missing in the plasmid construct method description.
- Clearly define intra- and inter-RNA interactions, both conceptually and in data analysis (methods line 569).
- Lines 585-588 should clarify whether sgRNA junctions were actually removed from chimeric reads and how this was handled.
- The identification of genomic deletions needs a clearer description in results, specifying that reference genomes were compared across genera.
- Define "reasonable" in line 690 for clarity.
- Address variations in RNA interactions between viruses and whether they could be due to different RNA-interaction methods.
- Provide details on detecting the negative RNA strand and its implications within the conclusions.
- Figure S20 shows what seems to be generally evenly distributed weak interactions, with the maximum signal of 6 reads, and it is unclear of it supports the conclusions of reproducible interactions. Similarly, a small difference is shown in fig S22. Please perform statistical tests.
- Fig 6E shows differences in host gene expression in cells expressing a putative PDCoV 3`UTR ORF. It is curious to know what the effect of this overexpression is on viral replication, and it may be easily tested by performing qPCR on these same samples with primers targeting viral RNA.

Reviewer #2:

The manuscript presents a comprehensive investigation into canonical and noncanonical sgRNA synthesis in coronavirus transcription and alludes to a role of non-canonical junctions in viral evolution. Through their combined analysis of novel RNA-seq and RIC-seq data, supported by publicly available datasets, the authors identify that canonical sgRNAs are associated with

same-direction RNA-RNA interactions whereas short non-canonical sgRNAs are associated with reverse-complementary RNA-RNA interaction, and then present an argument that viral genomic deletions correlate with short-range junctions. The authors conclude by investigating the role of non-canonical junctions as a functional genes.

As detailed by the authors, the current model for coronavirus transcript does not account for non-canonical sgRNA synthesis. Understanding the mechanism of coronavirus transcription has long been the aim of the coronavirus field and I believe this study will advance that aim. The conclusions are mostly supported by the data presented however I believe the manuscript could be further improved by the following.

1 - While the observation of condition-dependent shifts in canonical versus non-canonical junction frequency is interesting, it is limited to a minor subsection of the author's analysis of public datasets relating to MERS-CoV and some supporting PEDV infections. A conclusion that the ratio of canonical to non-canonical junctions is influenced by context-dependent selective pressures would not seem fully justified. The authors should consider further infections with varied experimental conditions (e.g. cell type, MOI, virus passage number, etc.) to fully support that conclusion.

2 - When discussing medium and long-range RNA:RNA interactions, and short-range non-canonical junctions' correlation to genomic deletions, the authors refer to a control dataset or analysis. One of the key findings of the manuscript is determinant on the interaction strength being higher than the controls. It is not sufficiently established as to what these controls are nor why these controls are necessary or appropriate. A short statement introducing these controls in either the results or methods section would be highly beneficial.

3 - The authors focus on SL1/2/3 as a driver of sgRNA synthesis in SARS-CoV-2 and describe the location of SL1/2/3 as being proximal to 3' end of the genome. By convention, SL1 through SL5 is used to describe stem-loops occurring in the 5' end of the genome. It is unclear what nomenclature the authors are using here and could lead to confusion. Furthermore, this section could be further improved by broadening the scope to either include analysis from other coronaviruses or by commenting or other coronaviruses.

4 - Discussion. The manuscript would benefit if the language, specifically in the first paragraph of the discussion, was toned downward. While I agree with the authors in their conclusion that sgRNA junctions facilitate genomic deletions, they have not demonstrated that sgRNAs themselves facilitate the deletion (Lines 446 - 448). Furthermore, while the authors can suggest that a balance between canonical and non-canonical sgRNA production is important for viral evolution, it has not been conclusively demonstrated here (Lines 450 - 453).

Minor issues:

5 - The authors provide some details in the methods which are seemingly extraneous. Line 592/593 for example does not add value to the manuscript. The authors might consider removing these sections and rewording some sentences to lower the word count without affecting the integrity of the manuscript.

Check the spelling of proportion across multiple figures.

Line 523/524: Cells were supplemented with 7.5 ug/mL trypsin

Line 526/533: 'In the late stage of replication' - what does this refer to?

Line 529: Provide units as RCF not as RPM.

Lines 555 - 588: it is currently unclear as to what the authors have done versus what the recommended pipeline is e.g. two different alignment programs are listed. Consider separating the processing of RNA-RNA interactome and RNA-seq data into different sections.

Line 561: 'We refer to adopt the pipeline' - poor phrasing.

Line 562: '...divided into the following 4 steps' - there are 5 steps listed.

Line 637: Please confirm that the order nucleotides on the x- and y-axis.

Line 666: 'we reverse sequences' - poor phrasing.

Line 669: The constraints placed here need to be explained to an audience not familiar with RNAfold.

Line 670-673: What were the selected parameters for the screen?

Dear Dr. Bheda,

Thank you for giving us the opportunity to revise our manuscript entitled "Cross-Genus Analysis Reveals Architecturally Programmed sgRNA Synthesis Patterns in Coronaviruses" (MSB-2025-13406-T). We are grateful to the reviewers for their insightful and constructive comments, which have helped us substantially improve the work. We have carefully addressed all the points raised, and we provide a point-by-point response below. Changes in the manuscript are highlighted in the revised text.

We believe that the revised manuscript is now stronger and suitable for publication in Molecular Systems Biology. Should you require any further information, please do not hesitate to contact us.

ORCID information for corresponding authors:

Due to a technical issue with the submission system, the ORCID for two corresponding authors could not be added. We kindly ask the editorial office to associate them with our manuscript:

- Fan Wang: https://orcid.org/0000-0003-2664-9214
- Li Li: https://orcid.org/0000-0002-9594-8341

Yours sincerely,

Dengguo Wei

National Key Laboratory of Agricultural Microbiology, College of Veterinary Medicine, Huazhong Agricultural University

No.1, Shizishan Street, Hongshan District, Wuhan, Hubei Province 430070, P.R.China

Tel.: +86 27 87282608

Fax: +86 27 87282608

Reviewer #1

Major points:

1.1 - The claim of increased interaction as a result of parallel base-pairing is surprising, given its rarity (PMID: 28771935). The authors should either strengthen their evidence for this claim or consider more plausible mechanisms, such as RBP or negative strand RNA-mediated interactions (figures 3C, S6-9). The correlation observed in figure S9B should also be examined for clarity, as it appears to be largely driven by a single sgRNA (ORF N).

We thank the reviewer for this insightful observation. The correlation in Figure S9B was re-examined. When all sgRNAs were included, a positive correlation was observed for most viruses (Pearson correlation coefficient range 0.371–0.701, except for PEDV) (Figure R1A). After removal of the N gene sgRNA, the correlation became weak positive or negative (PCC range –0.650 to 0.348) (Figure R1B), confirming that the original correlation was driven primarily by the N gene sgRNA. In light of this finding and recognizing that the correlation was not robust, this analysis has been removed entirely from the manuscript. The speculative parallel base-pairing predictions (Figure 3C and related supplementary figures) have also been deleted.

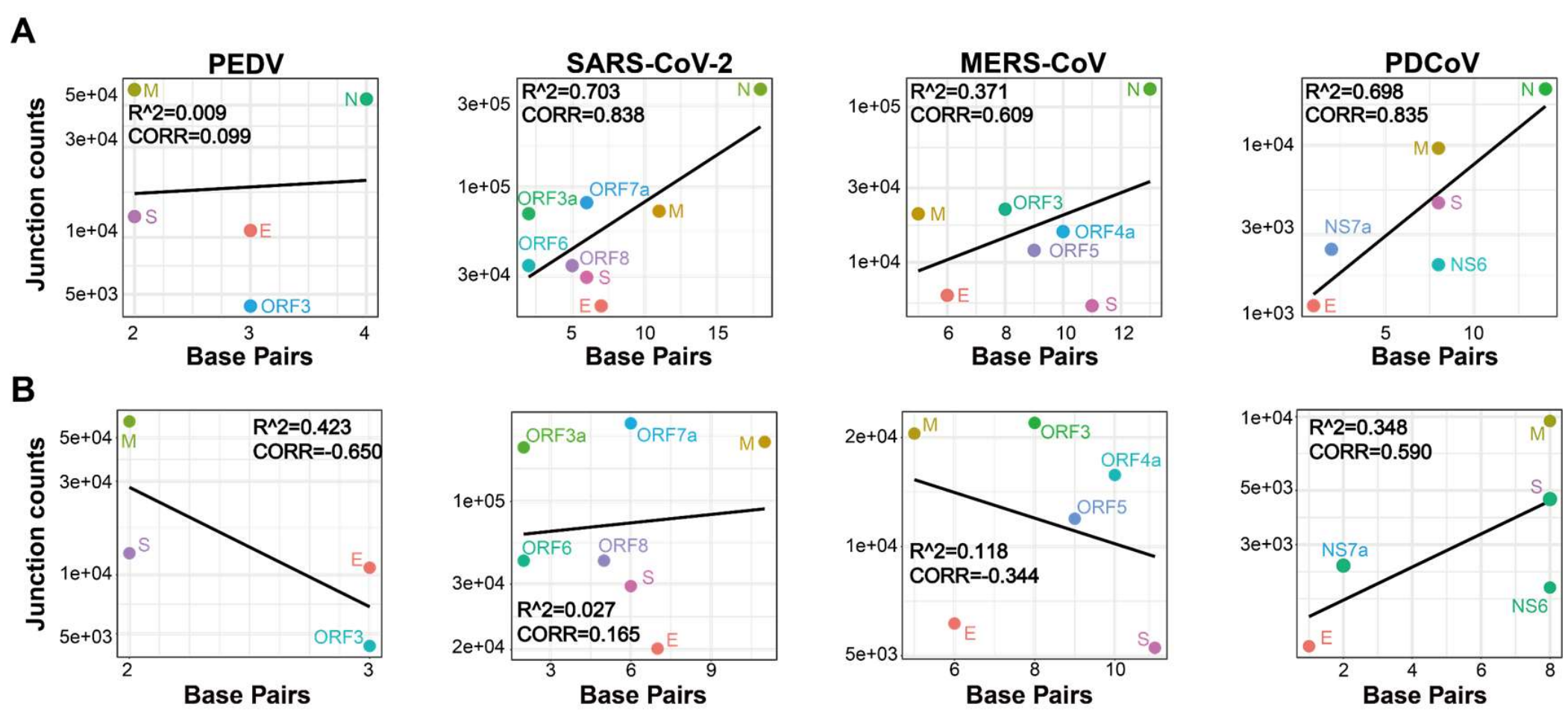

Figure R1. Correlation between canonical junction reads and interaction-associated base pairs in TRS-L/TRS-B flanking regions.

(A) All sgRNAs included. (B) N gene sgRNA excluded. Each panel represents one coronavirus (PEDV, SARS-CoV-2, MERS-CoV, PDCoV). Each dot corresponds to an individual sgRNA. The x-axis shows the number of base pairs in the predicted secondary structure that are associated with RNA-RNA interactions (highlighted in red in original figures). The y-axis shows the number of canonical junction reads.

As suggested by the reviewer, we now consider more plausible mechanisms for the observed diagonal enrichment in canonical junctions. Given the rarity of parallel

RNA-RNA base pairing, the text has been revised to focus on the robust observation of directional spatial proximity without overinterpreting the mechanism. In the Discussion (lines 546–548), we propose that the same-direction interactions likely reflect the requirement for template switching during negative-strand RNA synthesis, where the replication-transcription complex aligns TRS-L and TRS-B via the nascent negative strand. This interpretation does not rely on direct parallel base pairing between positive-strand RNA regions.

1.2 - The potential for detection of indirect RNA-RNA interactions should be addressed, as processes like discontinuous transcription can cause the observed proximity.

We thank the reviewer for raising this important concern. Proximity-ligation methods such as RIC-seq and COMRADES capture spatial contacts without distinguishing genuine inter-RNA interactions (direct base pairing or protein-mediated) from potential signals arising from discontinuous transcription intermediates. To specifically address the risk of false-positive signals from such intermediates, all chimeric reads corresponding to known sgRNA junctions were explicitly removed prior to constructing RNA-RNA interaction maps (see Methods, lines 678-682). Furthermore, the key interaction patterns (e.g., U-shaped distance profile, directional enrichments) were consistently observed across multiple independent methods (RIC-seq, COMRADES, SPLASH) and viruses, confirming that they are not artifacts of transient transcription intermediates. These measures are now described in the Discussion (lines 522–527).

1.3 - Speculative Claims: Overreaching statements regarding non-canonical sgRNAs as adaptive elements driving genome plasticity (in the abstract, introduction line 103, discussion line 451) lack robust supporting data. The evidence for any evolutionary roles remains unclear, especially given the suggested low expression levels during infection (as opposed to the high levels in the overexpression experiment).

We agree with the reviewer that our original wording overreached the evidence. In the revised manuscript, the overreaching statements have been removed or substantially toned down, as detailed below.

Abstract (lines 43–45): The claim “serve as adaptive elements to facilitate coronavirus adaptation” has been replaced with a more cautious statement that highlights a potential link.

Introduction (lines 95-99): The sentence “elucidated the roles... in viral evolution and host adaptation” has been revised to “offers insights into the potential involvement...”.

Discussion (lines 561–567): The phrase “critical determinant of viral evolvability” has been removed. The revised text now uses expressions such as “are associated with”, “may constitute”, and “may influence”, explicitly acknowledging the correlative nature of the data and the low abundance of non-canonical sgRNAs.

These revisions align our conclusions with the strength of the evidence. We thank the reviewer again for helping us improve the precision and rigor of the manuscript.

1.4 - The manuscript's analysis pipelines lacks a thorough description, both in the text and in the GitHub code, making it challenging to follow how specific analyses were conducted. Clear documentation would enhance reproducibility, and the current state does not allow reproducing the data.

We thank the reviewer for this important suggestion. To improve reproducibility, the following improvements have been made:

1) Methods section has been substantially expanded to include detailed descriptions of all analysis steps, software versions, parameters, thresholds and statistical tests. A Reagents and Tools Table has been added, listing key software with version numbers.

2) GitHub repository (https://github.com/WenZi0809/RNA-architecture-underlies-discontinuous-transcription-and-evolution-of-coronavirus) has been updated as follows:

   - The main analysis and plotting functions have been packaged and released on PyPI as corona-rna (version 0.1.0). Installation can be done via pip install corona-rna==0.1.0.

   - Jupyter Notebook files for reproducing all main figures have been provided.

   - A Nextflow pipeline for Corona mRNA has been added, together with usage instructions and example data.

These additions ensure that readers can fully understand and reproduce the analyses presented in the manuscript.

1.5 - There is no discussion of the low relative abundance of non-canonical versus canonical junctions, which is crucial for interpreting the results, particularly regarding

figure S21 where TPM calculated separately for each category is shown side by side.

We thank the reviewer for raising this essential point. In the revised manuscript, the low relative abundance of non-canonical versus canonical junctions has been explicitly discussed.

In the Results section (lines 423–426), a direct comparison has been added, showing that ORF10 sgRNAs are predominantly non-canonical (Appendix Figure S19B), whereas for all other SARS-CoV-2 genes, canonical sgRNAs vastly outnumber their non-canonical counterparts (Appendix Figure S21).

In the Discussion section (lines 592–598), a paragraph has been added that acknowledges the low overall abundance of non-canonical sgRNAs while highlighting the functional exception of ORF10 and other 3' terminal ORFs. The paragraph states that despite their low abundance, non-canonical transcription can play an essential role in expressing specific immune-modulatory genes.

1.6 - Figure 2B shows non-canonical junction relative abundance decreases with infection time and increases with virus passage. Since this is surprising and differs from previous results (e.g. PMID: 32906143, PMID: 33961822) and since this manuscript includes analysis of many RNA-seq data sets, including several timecourses, it would be helpful to perform a similar analysis on all available data to more carefully test this hypothesis. The data sets also include different cell types and drug treatments, which could also be incorporated into a more complete answer to the question of non-canonical sgRNA dynamics.

We thank the reviewer for this constructive suggestion. The analysis has been extended to all available RNA-seq datasets that include time-course, serial passage or other experimental perturbations. The results are summarized in a new supplementary figure (Appendix Figures S5-S6) and described in the revised Results (lines 161–178).

Key findings:

1) Passage effect: In five out of six independent serial passage datasets, the proportion of canonical junctions decreased with increasing passage number; one dataset showed no change. This indicates that long-term passaging generally reduces canonical transcription, leading to accumulation of non-canonical transcripts.

2) Infection time effect: The effect varied across viruses. For MERS-CoV and MHV, canonical junctions decreased over time; for SARS-CoV and IBV, they increased. For SARS-CoV-2, canonical junctions generally decreased over time, except for

transient increases in Vero E6 cells (24 to 48 hpi) and A549 cells (18 to 24 hpi). The observed overall downward trend in canonical junctions (i.e., increase in non-canonical junctions) in SARS-CoV-2 aligns with previous reports that non-canonical sgRNAs increase over time (PMID: 32906143, 33961822).

3) Other perturbations: Luteolin treatment markedly reduced canonical junctions, whereas NHC (EIDD-1931) treatment had minimal effect. Host gene perturbations (IRE1 knockout or ZBTB7A overexpression) did not significantly alter the ratio. A PEDV mutant strain showed a modest increase in canonical junctions compared to the wild-type.

The Results have been updated to reflect these broader, context-dependent dynamics, and the Discussion (lines 557–560) now emphasizes the multifactorial regulation of non-canonical transcription.

1.7 - Several key terms are inadequately defined:

o "non-canonical junctions", as used in this manuscript, is not clearly and consistently defined. Please clearly describe what consists as canonical or non-canonical junction, and what is excluded from these definitions. Is a junction between TRS-L and a non-TRS-B position considered canonical or non-canonical? Are these definitions consistent between the RNA-seq and nanopore pipeline analyses?

o The terms "divergent sequence pairs/orientations" and "directional RNA-RNA interactions".

o Line 167: do the numbers represent unique canonical junctions, or unique reads spanning canonical junctions?

o In figure 6 and S18-21 it is unclear how ORF10 sgRNA is identified in sequence data. What are canonical ORF10 sgRNA? This needs clarification especially since fig S21 shows an overwhelming majority of non-canonical sgRNAs are defined as ORF10 sgRNAs.

We have addressed the reviewer's requests as follows.

1) Definitions of canonical and non-canonical junctions

Clear definitions have been added in the Introduction (lines 57–59). Canonical junctions are defined as any discontinuous transcription event that involves the leader TRS (TRS-L), including junctions with known TRS-B sites as well as

junctions with other TRS-like sequences. Therefore, a junction between TRS-L and a non-TRS-B position is considered canonical. Non-canonical junctions are defined as events that do not involve TRS-L. These definitions are applied consistently in both RNA-seq and nanopore analyses.

2) Divergent sequence pairs and directional RNA-RNA interactions

In the Results (lines 187–191 and lines 237–241), divergent sequence pairs are now defined as pairs of genomic regions that participate in non-canonical junctions and exhibit high intra-pair sequence similarity (TRS-like). Directional RNA-RNA interactions are inferred from interaction heatmap patterns. Diagonal enrichment indicates same-direction interactions (parallel orientation), whereas anti-diagonal enrichment indicates convergent-direction (antiparallel) interactions.

3) Clarification of line 167 -- the number of canonical or non-canonical junctions

The numbers reported in line 167 (144,832 canonical junctions and 159,290 non-canonical junctions) represent unique read counts spanning canonical or non-canonical junctions, respectively, rather than the number of unique junction sites. The text has been corrected to: "we identified 144,832 reads spanning canonical junctions and 159,290 reads spanning non-canonical junctions."

4) Identification and classification of ORF10 sgRNAs

ORF10 sgRNAs were identified using the Corona-mRNA pipeline, which reconstructs full-length sgRNAs from nanopore reads (see Methods). The pipeline retains sgRNAs that contain the leader sequence, a complete gene reading frame, and the 3′ UTR; those formed by a single TRS-L to TRS junction are classified as canonical, all others as non-canonical. Specifically, a canonical ORF10 sgRNA arises from direct joining of the TRS-L (ACGAAC) to a sequence within 150 nt upstream of ORF10 that differs from the TRS motif by two mismatches (e.g., CCAAAC, AGAAAC, GCAAAC). Such junctions are classified as canonical because they involve TRS-L, even though the acceptor site is not a canonical TRS-B. Thus, the absence of a canonical TRS-B explains why canonical ORF10 sgRNAs are rare. The majority of ORF10 sgRNAs are non-canonical, arising from single N-ORF1a junctions or from double junctions that include a non-canonical component; under the pipeline's rules, any sgRNA containing a non-canonical junction is classified as non-canonical. This explains why ORF10 sgRNAs appear predominantly in the non-canonical category in original Figure S21 (now Appendix Figure S20).

We thank the reviewer for helping us improve the clarity and precision of the manuscript.

1.8 - Figure annotations and legends require more detail regarding experimental conditions, statistical tests, and subset clarifications.

We thank the reviewer for these important suggestions to improve the clarity and statistical rigor of our manuscript. All figure legends have been revised to include detailed information on experimental conditions, statistical tests, and subset clarifications. Specifically, each figure now specifies infection parameters (time, MOI, cell type, number of replicates), the statistical tests used with exact P values, and definitions of symbols, arrows, and highlighted regions. Representative examples are shown in the legends of Figures 2B, 3A-B and 6E.

1.9 - There is a lack of rigorous statistical analysis across the data sets, particularly concerning the robustness of results against a null hypothesis. Detailed randomization approaches should be reported to strengthen claims.

We thank the reviewer for highlighting the need for rigorous statistical testing against null hypotheses. The statistical analysis has been substantially strengthened throughout the manuscript. In the Methods section (lines 755–764), we now provide a detailed description of the randomization procedure, including the generation of control sets matched for number and distance distribution, the one-tailed t-test applied to APA center values, and the significance threshold ($p < 0.05$).

In addition, we report exact P values for all statistical comparisons (e.g., Figure 6E and Appendix Figures S9-11) and have added brief descriptions of the statistical tests in the corresponding figure legends. These revisions ensure that our claims of enrichment or significance are properly supported by rigorous null-hypothesis testing.

1.10 - Quality control for sequencing libraries is largely missing, both for newly generated libraries (other than replicate correlation) and for the analysis itself, please provide QC analyses such as:

o % human vs virus reads

o % canonical vs non-canonical junctions

o Detection of virus-host RNA interactions

o Recapitulation of known structures (e.g. 5` and 3` UTR stem loops, TRS-L stem loops and others predicted from sequence and previous works) and junctions.

Comprehensive quality control (QC) analyses requested by the reviewer have been performed for all newly generated RIC-seq and RNA-seq libraries. The QC metrics, including the percentage of human versus viral reads, the proportion of canonical versus non-canonical junctions, detection of virus-host RNA interactions, recapitulation of the 5′ and 3′ UTR stem-loops, and pCp ligation efficiency, are summarized in Appendix Figures S1–S3. In addition, replicate reproducibility was high: Pearson correlation coefficients for chimeric read coverage ranged from 0.8480 to 0.9974, and for interaction strength from 0.8926 to 0.9987 (Appendix Figures S1–S2). These results confirm the quality of our sequencing data and the validity of the subsequent analyses.

Minor points:

2.1 - The manuscript could benefit from streamlining the data presented in the supplements and enhancing the focus on the main findings.

In the revised manuscript, redundant figures have been removed (e.g., the distribution of non-canonical junction proportions in Figure S2B and all parallel-pairing predictions), and related panels have been combined across viruses (e.g., Appendix Figure S16). New supplementary figures have been added to directly support the main findings, for example, quality control analyses (Appendix Figures S1–S3) and the extended dynamics of non-canonical transcription across time courses, serial passages, and other perturbations (Appendix Figure S4).

2.2 - Figure legends need additional annotations:

o Include descriptions of infection/transfection time, MOI, statistical significance tests, and number of replicates in all relevant legends.

o Clarify whether all junctions or a subset is shown, and the criteria for any subsetting.

o Define arrows and dashed lines in S2A.

o Add annotations for highlighted elements in Fig 2C (red dots, starred area) and specify whether all or only non-canonical junctions are shown.

o Provide additional genome annotations for APA figures, including directionality with arrows and clarifications regarding the meaning of + signs in Fig 2A, especially the meaning of the double + signs between the 3rd and the 4th columns (M and N).

o Expand descriptions of sashimi plots in legends of figures 5E and S15-16.

o Explain the gray point labels in fig 5G-H and S15A, and highlight stem loops in S15A.

o Describe the different lines in fig 6B for improved clarity.

All figure legends have been revised to include the requested information (infection parameters, statistical tests, definitions of symbols, explanations of highlighted elements, and clarifications of junction types and read alignments). The updated legends are present in the main text and the Appendix.

2.3 - TRS-like sequences: confirm if the entire genome was searched for TRS-like pairs and how regions I, II, and III were selected in Fig 2C. Include quantitative measures for TRS-like sequences and their associations with RNA-RNA interactions.

The identification of TRS-like sequences and their association with RNA-RNA interactions have been clarified in the revised manuscript as follows.

1) Genome-wide search for TRS-like pairs

   The entire genome of each coronavirus was scanned to identify TRS-like sequence pairs. For every non-canonical junction detected by RNA-seq, the 6-nucleotide sequences immediately flanking the breakpoints were extracted. Each pair was evaluated for sequence similarity using a custom alignment that accounts for wobble pairing (G-U) in RNA (see Methods, lines 724–743). Pairs with a local alignment score ≥4 were designated as TRS-like. This process was applied to all non-canonical junctions across the entire genome, ensuring no positional bias.

2) Selection of regions I, II, and III in Figure 2C

   Regions I, II, and III in Figure 2C were chosen as representative examples of short-range (<1.7 kb), mid-range (1.7–22 kb), and long-range (>22 kb) non-canonical junctions, respectively. These specific pairs were selected because they each exhibit high intra-pair sequence consistency (score 6.0 for each) and are located in distinct genomic contexts (structural protein-coding regions for short-range ORF1a–N for long-range). They illustrate the diversity of genomic distances at which TRS-like sequences can mediate non-canonical transcription.

3) Association of TRS-like sequences with RNA-RNA interactions

   To quantitatively assess whether TRS-like sequences are associated with spatial proximity, aggregate peak analysis (APA) was performed as described in the

Methods (lines 745–764). Briefly, for each set of TRS-like pairs and matched random controls, 100 nt × 100 nt submatrices centered on each pair were extracted from the interaction matrix (5 nt resolution) and summed to generate APA heatmaps. Enrichment at the center indicates spatial proximity. As shown in Figures 4A–C, TRS-like pairs exhibited significant central enrichment compared to random controls (one-tailed t-test, $P < 0.05$ for all comparisons). These results confirm that TRS-like sequences are associated with genuine RNA-RNA interactions that bring the paired regions into spatial proximity.

2.4 - Line 66: Reference 10 does not support the claim that non-canonical sgRNAs encode functional polypeptides, please adjust the sentence to more appropriately reflect the paper's results.

Upon re-examination of reference 10 (PMID: 36075564), we agree that the original phrasing "encode functional polypeptides" overstates the paper's conclusions. Wu et al. identified many non-canonical sgRNA-derived peptides by mass spectrometry and showed that these peptides increase after infection and may have RNA-binding and immunogenic potential, but direct functional characterization was not performed. The sentence (line 66) has therefore been revised to: "non-canonical sgRNAs could be translated into polypeptides" This correction accurately reflects the evidence presented in ref 10.

2.5 - Describe how the data sets were collected and clarify the reasons for excluding certain datasets. Include information on which data sets went into each analysis. This should be part of the main text and possibly figure 1.

We thank the reviewer for this suggestion. The description of public data collection has been added to the Methods section (new subsection "Public datasets", lines 802–808) and states that all public coronavirus transcriptome (RNA-seq and nanopore) and RNA-RNA interactome datasets were collected from NCBI, EBI, BIG, and OSF. The filtering criteria for RNA-seq data (viral genome mapping efficiency >0.5% and >5,000 junctions per sample) are described in the Results (lines 105–107), where it is stated that 103 of 165 RNA-seq samples were retained. All public datasets are listed in a supplementary file (Dataset EV6_data resources.xlsx) with accession numbers, experimental conditions, and the analyses they contributed to.

2.6 - Clarify how Fig 2D and S2A account for size differences between viruses.

We thank the reviewer for raising this important point. In the original figures, absolute genomic distances (kb or nt) were used. To verify that the observed U-shaped distribution is not an artifact of genome size variation, distances were also normalized by total genome length (fraction of genome; genomes were divided into 100 equal bins to obtain relative distances) and plotted in Appendix Figures S4B and S7F, which show the same U-shaped pattern. This confirms that the conclusion holds regardless of genome length.

2.7 - Explain the inclusion of Fig S2B, as it appears to present the same data as S2C and 2A.

We thank the reviewer for pointing out the redundancy. Figure S2B was originally included to provide an overview of the distribution of non-canonical junction proportions across all 103 RNA-seq samples. However, we agree that this information is already captured in Figure 2A (where the color scale in the clustering heatmap indicates the proportion) and Figure S2C (which shows detailed proportions for SARS-CoV-2 and MERS-CoV). Therefore, we have removed Figure S2B from the revised manuscript to avoid duplication.

2.8 - The deposited PEDV RNA-seq includes two replicates, but not the data from the experiment in fig 2B.

We acknowledge that the original Data Availability statement lacked clarity. The RNA-seq data deposited under BioProject PRJCA026550 (two replicates) were generated alongside the PEDV RIC-seq libraries and are used to analyze the relationship between RNA-RNA interactions and junction patterns (see QC in Appendix Figures S1–S3). These replicates are not the data shown in Figure 2B.

Instead, the PEDV time-course experiment in Figure 2B was obtained from a public dataset (NCBI BioProject PRJNA801927). This dataset was re-analyzed to independently illustrate how non-canonical junction proportions change under different experimental conditions (passage number, infection time), serving as a preliminary example before the expanded analysis across all viruses. To clearly distinguish the two data sources, the Data Availability section has been revised to list only the newly deposited data, and the source of the public dataset is now indicated in the legend of Figure 2B.

2.9 - Provide a complete description of the APA method, clarifying how it was applied

and how data deviates from null/chance results, and discuss the meaning of the cross-shaped patterns in APA data (e.g. in fig 4C).

We have revised the APA method description to clarify that the APA heatmap aggregates hundreds of junction sites into a single distribution, and that a single matched random control set (same number of pairs, same distance distribution) was used to generate a control APA heatmap (lines 755–764). A one-tailed t-test comparing the center values of the observed and control APA heatmaps was used to assess statistical significance ($p < 0.05$). This approach directly quantifies how the observed spatial proximity deviates from null expectations.

In the Results section (lines 331–333), we have added a brief description that the APA heatmap for long-range junctions displays a weak cross-shaped pattern consisting of horizontal and vertical stripes intersecting at the center, whereas mid-range junctions show only central enrichment. This cross-shaped pattern is reminiscent of the topological features of protein-mediated chromatin loops in eukaryotic Hi-C maps (PMID: 25497547), where cohesin and other factors generate characteristic corner stripes. In the Discussion (lines 551–552), we therefore propose that long-range non-canonical junctions are brought into proximity by RNA-binding proteins that tether multiple nearby sites, producing the observed stripes.

2.10 - For all RNA structures shown, please show the predicted ΔG from mFold for each selected structure as well as the lowest (most negative) ΔG outputted of all of the potential structures predicted, to give a sense of the thermodynamic favorability of the parallel strand formation versus other structural ensembles.

As detailed in our response to Comment 1.1, all parallel-pairing RNA structure predictions (original Figure 3C and Figures S6–S9) have been removed from the manuscript. Consequently, the requested comparison of ΔG for parallel strand formation versus other structural ensembles is no longer applicable.

For the remaining conventional stem-loop structures (e.g., 3'SL1-3 in SARS-CoV-2, and the predicted UTR structures for PEDV and PDCoV in the QC analysis), we have added the predicted ΔG of the depicted folds (calculated by RNAfold) to the corresponding figure legends (e.g., Figure 5F and Appendix Figure S3). We did not calculate the global minimum free energy for all possible folds because our structures are constrained by experimentally supported RNA-RNA interactions and represent the biologically relevant conformations, not necessarily the thermodynamic minima.

2.11 - Please address the deviation of PDCoV from the results more directly. Is it a true difference from other viruses, a non-significant difference, or a result of the lower reproducibility of PDCoV data?

We performed additional analyses and confirmed that the weaker APA signals observed for PDCoV (fainter anti-diagonal pattern in Figure 4C and less pronounced diagonal in Figure 3B) represent a true biological difference, not a non-significant result or an artifact of lower data reproducibility.

RNA secondary structure properties were compared across the four coronaviruses. PDCoV exhibits the shortest average base-pairing distance and the smallest number of consecutive paired bases (Appendix Figure S13). This indicates that PDCoV tends to form smaller and less extensive local secondary structures, which naturally yields weaker APA enrichment signals.

Although one PDCoV RIC-seq replicate (rep3) showed lower correlation (PCC 0.848 and 0.882) with the other two replicates, APA heatmaps generated from each replicate individually were examined. All three individual replicates exhibited essentially identical patterns, including the weaker anti-diagonal enrichment (Figure R2). Thus, the deviation is robust and not driven by replicate variability.

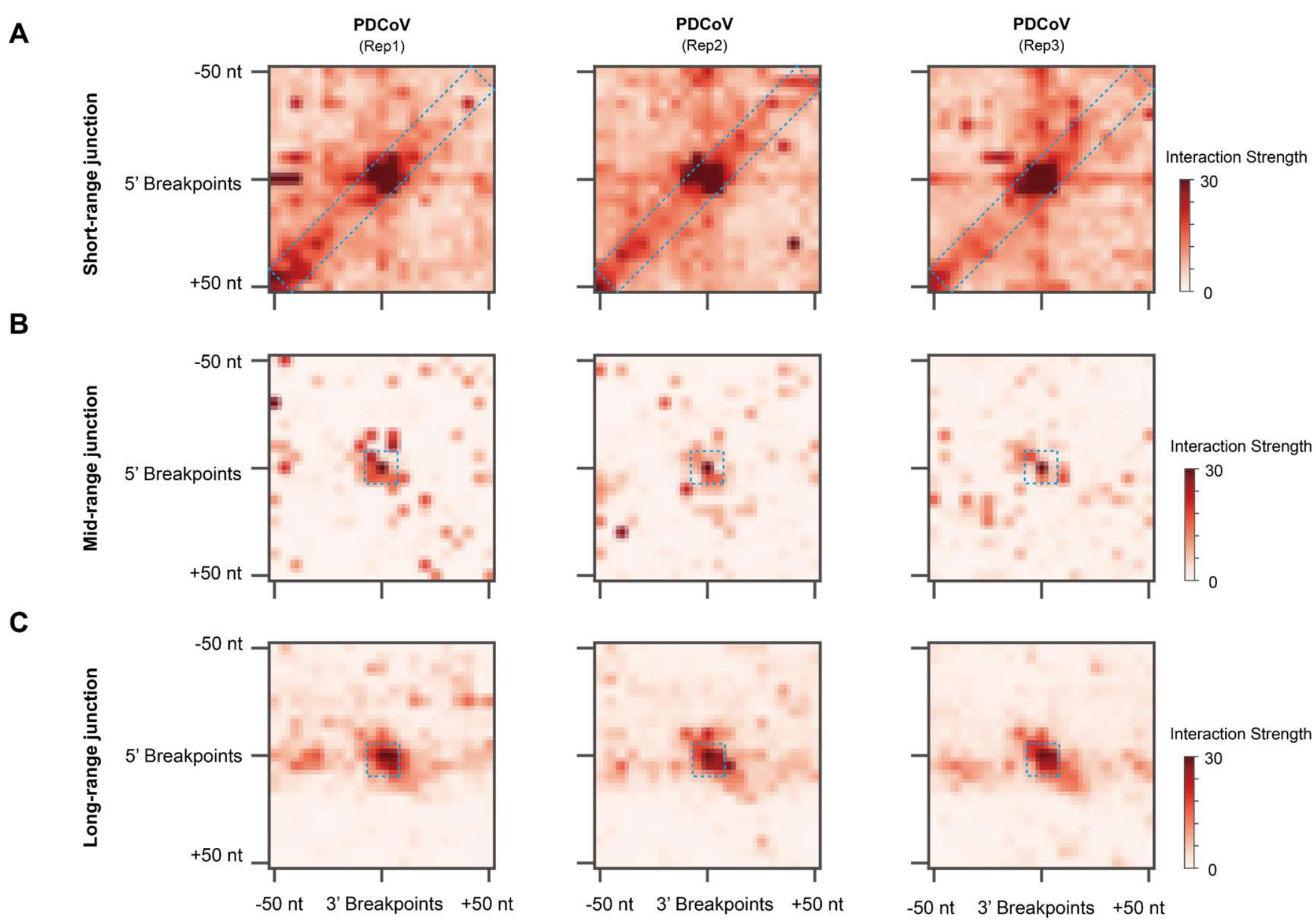

Figure R2. APA heatmaps of 3 PDCoV replicates.

(A-C) APA heatmaps showing aggregated RNA-RNA interactions from short-range (A), mid-range (B) and long-range (C) non-canonical junction site-flanking regions of 3 PDCoV replicates. Each heatmap sums 100 nt × 100 nt submatrices centered on each junction site, where the axes represent

genomic distance (nt) relative to the junction site center. Color intensity (red) reflects interaction frequency, with darker red indicating stronger interactions.

These analyses have been added to the manuscript: the structural boxplots are in Appendix Figure S13, and a concise explanation is included in the Discussion (lines 529–534).

2.12 - Include a table with detected genomic deletions and their sizes. Could deletion size differences between viruses explain the different results in fig 5A-D?

We thank the reviewer for this suggestion. A detailed list of all genomic deletions (>20 nt) for each virus has been compiled, including coordinates, sizes, overlap with predicted stem-loops and affected genes. This list is provided as Dataset EV5 (Excel file, one sheet per virus). To assess whether deletion size differences could explain the varying APA patterns in Figures 5A–D, we compared mean deletion sizes across viruses. The summary statistics are as follows (Table R1):

Table R1. Deletions obtained based on multiple sequence alignment

Virus	Mean deletion size (nt)	Median deletion size (nt)	Standard deviation
SARS-CoV-2	98.22	64	84.94
PEDV	150.00	46	203.97
MERS-CoV	83.65	33	139.64
PDCoV	127.00	79	120.42

Notably, PDCoV (pronounced anti-diagonal pattern in Figures 5A–D) and SARS-CoV-2 (weak anti-diagonal pattern) have very similar deletion size statistics (mean 127 vs. 98 nt; median 79 vs. 64 nt; standard deviation 120 vs. 85). Despite these similarities, their APA patterns are markedly different. This indicates that deletion size does not explain the observed differences.

In contrast, the proportion of deletions overlapping stem-loops (PDCoV 62.5%, PEDV 45.2%, SARS-CoV-2 29.5%, MERS-CoV 23.5%) correlates well with the anti-diagonal signals. This suggests that stem-loop association, rather than deletion size, is the primary driver of the observed APA patterns.

2.13 - Line 334, please refer to fig S15A.

We have added the reference to Figure S15A (now Appendix Figure S16A) at the indicated line in the revised manuscript (line 366).

2.14 - Confirm if putative 3' UTR ORFs sgRNAs in other viruses are supported by the analyzed data.

We thank the reviewer for this question. Our original RNA-seq analysis detected recurrent N-ORF1a junctions in six coronaviruses (SARS-CoV-2, SARS-CoV, MERS-CoV, PEDV, IBV and PDCoV), representing four genera.

To further confirm that these junctions are part of full-length sgRNAs, we examined publicly available nanopore sequencing data. In addition to SARS-CoV-2, we identified nanopore reads spanning the ORF1a-N junction and containing the downstream 3′ UTR region for PEDV, MERS-CoV, MHV and HCoV-229E, thus directly confirming the existence of $sgRNA_{N\text{-}ORF1a}$ transcripts in these viruses. These reads are presented in a new supplementary figure (Appendix Figure S22) using a visualization format similar to Figure 6B.

For IBV, the available nanopore reads (537 reads; see Dataset EV2) were too few to allow reliable validation of full-length sgRNAs; consequently, IBV was excluded from the nanopore analysis. For SARS-CoV and PDCoV, no public nanopore data were available. Nevertheless, the RNA-seq junction evidence (Appendix Figure S21A) supports the presence of similar transcripts in these viruses.

We have added a description of these nanopore validation results in the Results section (lines 442–444) and included the new figure in the Appendix.

2.15 - Include exact time of infection in library preparation methods, along with the number of cells and MOI.

We thank the reviewer for this suggestion. The Methods section has been revised to include the requested details:

1) In Cell culture and virus infection, we now state: "Vero E6 cells (1×10^7) were infected with PEDV AJ1102 at an MOI of 0.1 for 12 hours. LLC-PK1 cells (1×10^7) were infected with PDCoV CHN-HN-2014 at an MOI of 0.1 for 10 hours."

2) In RIC-seq library preparation and RNA-seq library preparation, we have replaced the vague phrase "In the late stage of replication" with the exact time points (12 hpi for PEDV, 10 hpi for PDCoV) and also included the cell number and MOI for clarity.

These changes are located in lines 616–623 of the revised manuscript.

2.16 - A reverse transcription step is missing in the plasmid construct method description.

We thank the reviewer for catching this omission. In the revised manuscript, the reverse transcription step has been added to the Plasmid constructs section. Specifically, after RNA extraction, first-strand cDNA was synthesized using a reverse transcription kit (HiScript II Q RT SuperMix, Vazyme). The cDNA was then used as template for mutagenesis and cloning into the pCAGGS-Flag vector. The revised text is located in the Methods section (lines 636–638).

2.17 - Clearly define intra- and inter-RNA interactions, both conceptually and in data analysis (methods line 569).

We thank the reviewer for this suggestion. The Methods section (lines 656–662) has been revised to provide clear definitions of intra- and inter-RNA interactions, both conceptually and in data analysis, based on the RIC-seq protocol (PMID: 34021296). Briefly, intra-RNA interactions are defined as read pairs from random self-ligation within the same RNA molecule (experimental background), whereas inter-RNA interactions are defined as pCp-labeled read pairs from proximity ligation between two separate RNA molecules (e.g., RNA-binding proteins or base-pairing). In step 3 of the pipeline, paired reads are accordingly categorized as normal (intra-RNA) and chimeric (inter-RNA).

2.18 - Lines 585-588 should clarify whether sgRNA junctions were actually removed from chimeric reads and how this was handled.

We thank the reviewer for this clarification. In the revised manuscript (lines 678–681), it has been explicitly stated that sgRNA junction-derived chimeric reads were removed from the RNA-RNA interactome analysis. A list of junction coordinates was compiled from matched RNA-seq samples, and any chimeric read whose breakpoints matched these coordinates was filtered out from the RIC-seq, COMRADES and SPLASH data. This step ensures that the interaction maps reflect genuine RNA-RNA contacts rather than transcription intermediates.

2.19 - The identification of genomic deletions needs a clearer description in results, specifying that reference genomes were compared across genera.

We thank the reviewer for this clarification. In the revised Results section (lines 343–346), it has been explicitly stated that genomic deletions were identified independently for each coronavirus species using a species-specific reference genome (PEDV: MK584552; SARS-CoV-2: NC_004718; MERS-CoV: NC_019843; PDCoV: KT336560). No cross-genus alignment was performed. This ensures readers understand that the deletion numbers and locations are species-specific and not directly comparable across genera in absolute terms.

2.20 - Define "reasonable" in line 690 for clarity.

We thank the reviewer for pointing out the ambiguous term. In the revised manuscript (lines 798–800), the phrase “manual examination” and “reasonable” have been removed, and it is now explicitly stated that ORFs meeting the three predefined criteria (≥10 amino acids, initiation codon AUG/GUG/UUG, and located within a long-range non-canonical sgRNA) were considered candidate ORFs. This eliminates any subjectivity and aligns with the objective nature of the screening.

2.21 - Address variations in RNA interactions between viruses and whether they could be due to different RNA-interaction methods.

We thank the reviewer for raising this important point. The cross-virus comparisons of APA enrichments (e.g., Figures 3B, 4A-C) were based primarily on data obtained by the same method (RIC-seq) for PEDV, PDCoV, and SARS-CoV-2; only MERS-CoV data were generated by COMRADES. Therefore, the weaker anti-diagonal signal in PDCoV (Figure 4C) cannot be attributed to methodological differences; it likely reflects genuine biological differences, such as the distinct RNA folding architecture of δ-coronaviruses.

To further confirm that different methods are comparable, key architectural features, including the U-shaped distance-dependent interaction profile (Figure S2A), same-directional enrichment for canonical junctions (Figure 3B), and anti-diagonal patterns for short-range non-canonical junctions (Figure 4A), are consistently observed across all methods and viruses. In addition, for SARS-CoV-2, three independent methods (RIC-seq, SPLASH, COMRADES) produced highly consistent APA patterns (Appendix Figure S10). Thus, methodological biases do not distort the core conclusions. This interpretation has been added to the Discussion (lines 521–529).

2.22 - Provide details on detecting the negative RNA strand and its implications within

the conclusions.

We thank the reviewer for this important question. All sequencing datasets used in this study were derived from strand-specific protocols. For each dataset, reads were aligned separately to the positive and negative strands. The proportion of reads mapping to the negative strand was below 5% in the majority of samples. For example, in PDCoV RIC-seq samples, negative strand reads accounted for only 0.05% to 0.2% of viral reads (Table R2). All analyses presented in this study, including the identification of sgRNA junctions and RNA-RNA interactions, were performed exclusively on positive strand-derived reads. Therefore, the conclusions are not confounded by negative strand RNA.

Table R2. PDCoV strand-specific read proportions

Replicate	Total reads	Positive strand reads (%)	Negative strand reads (%)	Reads with ends on different strands (%)
1	947,852	99.40%	0.13%	0.47%
2	1,462,614	99.58%	0.08%	0.34%
3	1,578,964	99.40%	0.12%	0.48%

2.23 - Figure S20 shows what seems to be generally evenly distributed weak interactions, with the maximum signal of 6 reads, and it is unclear of it supports the conclusions of reproducible interactions. Similarly, a small difference is shown in fig S22. Please perform statistical tests.

We thank the reviewer for this request. Statistical tests have been performed for both figures.

1) For Appendix Figure S21 (originally Figure S20)

    100 random control regions matched in length and distance distribution were selected for each virus. For most viruses, the observed interaction counts were significantly higher than random controls (Wilcoxon rank-sum test, $p < 0.01$). For PDCoV, due to its smaller genome, only 30 non-overlapping control regions could be selected. Among these, 19 showed a fold change >1.2 with statistical significance, while the remaining 11 had fold changes within ±1.2 (not significant). All other viruses exhibited significant enrichment for all 100 controls, with the sole exception of one control region in SARS-CoV-2, which showed a fold change <1.2 (but remained statistically significant).

2) For Appendix Figure S23 (originally Figure S22)

The observed interaction counts (from samples with RNA ligase) and their ratio to the negative control (without RNA ligase) were both significantly lower at 48 hpi than at 24 hpi (two-tailed Wilcoxon rank-sum test, $p < 0.001$).

These results confirm that the observed interactions and temporal differences are not due to chance.

2.24 - Fig 6E shows differences in host gene expression in cells expressing a putative PDCoV 3`UTR ORF. It is curious to know what the effect of this overexpression is on viral replication, and it may be easily tested by performing qPCR on these same samples with primers targeting viral RNA.

We appreciate the reviewer's suggestion. Consistent with this, we had included an analysis of viral replication in our initial study. As shown in Appendix Figure S24B, LLC-PK1 cells overexpressing ORF_{UTR3} exhibited significantly higher levels of PDCoV N gene transcripts compared to control cells (empty vector) upon viral infection (two-tailed t-test; P-value = 0.040), indicating that ORF_{UTR3} enhances viral replication. Together with the observation that ORF_{UTR3} suppresses IFN-α1, IFN-β, ISG15 and OAS1 expression (Figure 6E), these data suggest that ORF_{UTR3} promotes PDCoV replication by attenuating the host antiviral innate immune response. This finding has been described in the Results section (lines 515–517).

Reviewer #2:

1.1 - While the observation of condition-dependent shifts in canonical versus non-canonical junction frequency is interesting, it is limited to a minor subsection of the author's analysis of public datasets relating to MERS-CoV and some supporting PEDV infections. A conclusion that the ratio of canonical to non-canonical junctions is influenced by context-dependent selective pressures would not seem fully justified. The authors should consider further infections with varied experimental conditions (e.g., cell type, MOI, virus passage number, etc.) to fully support that conclusion.

We thank the reviewer for this constructive suggestion. The following revisions have been made.

First, the language regarding selective pressures has been substantially toned down. The revised manuscript now states that the ratio of canonical to non-canonical junctions varies under different experimental conditions, suggesting dynamic regulation, without claiming a direct link to selective pressure (Results, lines 175–178).

Second, the analysis has been expanded to all available RNA-seq public datasets that include varied conditions (cell types, MOIs, passage numbers, drug treatments, and viral mutants). This extended analysis (Appendix Figures S5–S6) revealed that:

1) The effect of infection time varies across viruses (Appendix Figure S5): for MERS-CoV and MHV, the proportion of canonical junctions decreases over time; for SARS-CoV and IBV, it increases; for SARS-CoV-2, it generally decreases except for transient increases in Vero E6 cells (24–48 hpi) and A549 cells (18–24 hpi).

2) The accumulation of non-canonical junctions with serial passage is a general phenomenon, observed in five out of six independent passage datasets (Appendix Figure S6A).

3) Certain perturbations markedly alter the ratio (Appendix Figures S6B–E), such as luteolin treatment or a PEDV mutant strain, whereas others (e.g., NHC treatment, IRE1 knockout, ZBTB7A overexpression) have minimal impact.

Thus, the available public data already cover a wide range of experimental conditions and support the conclusion that non-canonical transcription is conditionally regulated. Given that the expanded analysis encompasses diverse conditions, additional infection experiments are not considered essential to support the revised conclusion. The Results section has been updated accordingly (lines 160–174), and overreaching statements about selective pressure have been removed. The revised conclusion reads: "coronavirus transcription exhibits complex, context-dependent dynamics, influenced by factors including passage history, infection time, viral genetic background, and specific environmental cues".

We thank the reviewer for helping us strengthen the evidence base for this observation.

1.2 - When discussing medium and long-range RNA-RNA interactions, and short-range non-canonical junctions' correlation to genomic deletions, the authors refer to a control dataset or analysis. One of the key findings of the manuscript is determinant on the interaction strength being higher than the controls. It is not sufficiently established as to what these controls are nor why these controls are necessary or appropriate. A short statement introducing these controls in either the results or methods section would be highly beneficial.

We thank the reviewer for this important clarification. The nature and purpose of the control sets have been explicitly stated in the Results and Methods sections. The

controls are generated by randomly selecting genomic site pairs matched to the test set in number and inter-site distance distribution (binned into 5 nt intervals). This matching eliminates distance-dependent background, ensuring that observed enrichment reflects genuine spatial proximity. In the Results, brief descriptions of the controls have been added at the first appearance of APA for canonical junctions (lines 247–249) and for deletions (lines 350–352). The Methods section has also been expanded accordingly (lines 755–764).

1.3 - The authors focus on SL1/2/3 as a driver of sgRNA synthesis in SARS-CoV-2 and describe the location of SL1/2/3 as being proximal to 3' end of the genome. By convention, SL1 through SL5 is used to describe stem-loops occurring in the 5' end of the genome. It is unclear what nomenclature the authors are using here and could lead to confusion. Furthermore, this section could be further improved by broadening the scope to either include analysis from other coronaviruses or by commenting on other coronaviruses.

We thank the reviewer for this important clarification. The original nomenclature (SL1/2/3) has been renamed to 3'SL1, 3'SL2 and 3'SL3 throughout the manuscript, including all figures and legends, to avoid confusion with the canonical 5' stem-loops. A clarifying sentence has been added at first appearance (Results, lines 364–366).

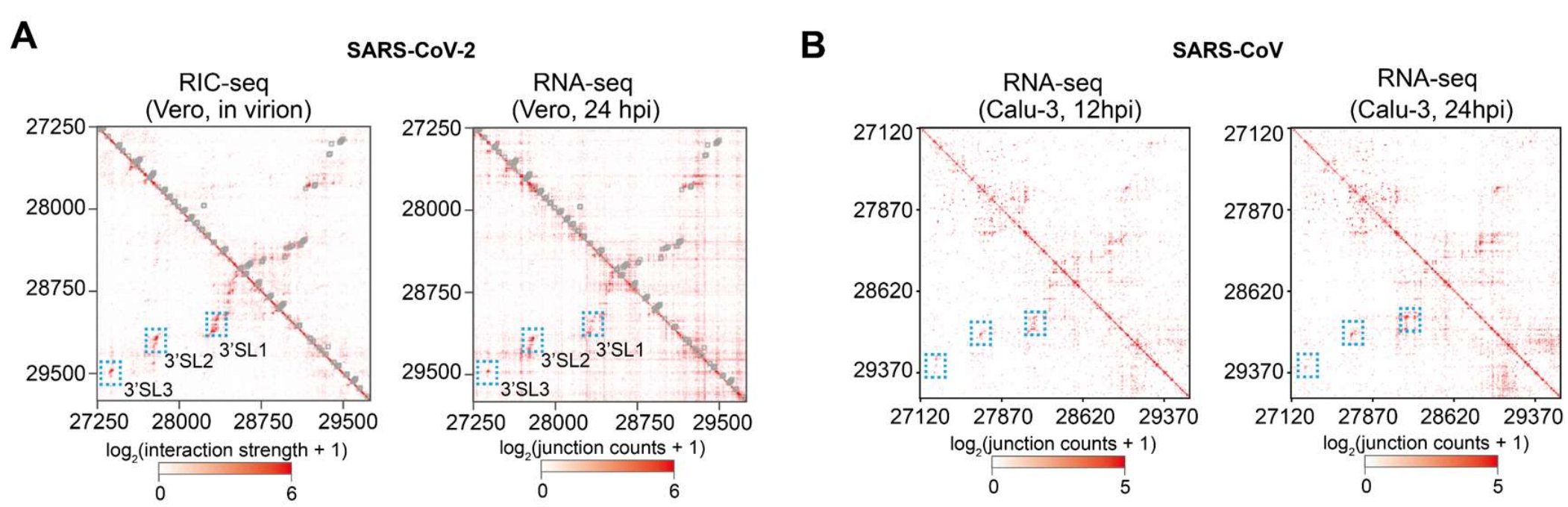

Figure R3. Non-canonical junctions in the 3′SL1-3 region of SARS-CoV-2 and its homologous region in SARS-CoV.

(A) The 3′SL1-3 region of SARS-CoV-2. Two subpanels are shown. The first subpanel displays an RNA-RNA interaction heatmap from RIC-seq data (Vero cells, in virion). The second subpanel shows short-range non-canonical junctions from RNA-seq data (Vero cells, 24 hpi). Grey dots in the upper-right corner indicate predicted base-pairing interactions derived from the RNA secondary structure (each grey dot represents a paired nucleotide). Larger blue boxes in the lower-right corner highlight the specific base pairs that constitute the 3′SL1, 3′SL2 and 3′SL3 stem-loops, which are supported by high-intensity RNA-RNA interactions. (B) The region homologous to 3′SL1-3 in SARS-CoV. Two subpanels show short-range non-canonical junctions from RNA-seq data (Calu-3 cells, 12 hpi and 24 hpi). Larger blue boxes indicate the positions of the homologous region.

To broaden the scope, the 3′ terminal regions of several coronaviruses with available RNA-RNA interaction data (SARS-CoV-2, MERS-CoV, PEDV, PDCoV) were examined. An analogous structural arrangement is supported by interaction data only in SARS-CoV-2. For SARS-CoV, although RNA-RNA interaction data are not available, RNA-seq reads reveal non-canonical junctions within the region homologous to 3'SL1-3 similar to those observed in SARS-CoV-2 (Figure R3), suggesting that a similar structure may exist. In MERS-CoV, PEDV and PDCoV, the 3' terminal ORFs (ORF_{UTR3}) are instead expressed via a different non-canonical mechanism, namely the long-range ORF1a-N junction that generates $sgRNA_{N\text{-}ORF1a}$. This cross-virus diversity has been noted in the Discussion (lines 594–598).

1.4 - The manuscript would benefit if the language, specifically in the first paragraph of the discussion, was toned downward. While I agree with the authors in their conclusion that sgRNA junctions facilitate genomic deletions, they have not demonstrated that sgRNAs themselves facilitate the deletion (Lines 446 - 448). Furthermore, while the authors can suggest that a balance between canonical and non-canonical sgRNA production is important for viral evolution, it has not been conclusively demonstrated here (Lines 450 - 453).

We thank the reviewer for this careful assessment. The original discussion language was too strong given the correlative nature of the data; association between non-canonical sgRNAs and deletion hotspots has been shown, but not causation, and evolutionary implications remain speculative. In the revised manuscript, the original first paragraph has been reorganized and is now located as the third paragraph of the Discussion (lines 561–567). Its language has been substantially toned down, using expressions such as "are associated with" instead of "facilitate", "may influence" instead of "is a critical determinant", and "speculate... may constitute" instead of "position as a critical determinant". It is also explicitly acknowledged that direct functional evidence is still lacking.

Minor issues:

2.1 - The authors provide some details in the methods which are seemingly extraneous. Line 592/593 for example does not add value to the manuscript. The authors might consider removing these sections and rewording some sentences to lower the word count without affecting the integrity of the manuscript.

We thank the reviewer for this suggestion. The entire Methods section has been systematically reviewed and the following improvements have been made:

1) Overly basic descriptions have been removed (e.g., explanation of FAST5 to FASTQ conversion, details of file formats);

2) Redundant phrases have been shortened and related sentences merged;

3) All critical parameters, software versions and analysis steps have been retained to ensure full reproducibility.

The revised Methods section is now more concise while preserving all essential information. The changes are distributed throughout the section (see lines 695–700 for key revisions).

2.2 - Check the spelling of proportion across multiple figures.

We thank the reviewer for catching this typographical error. The word "proportion" was misspelled as "propotion" (missing the second "r") in Figures 2B and 2E. This has been corrected. All other occurrences of the word "proportion" in figures have been verified and are spelled correctly. The revised figures have been uploaded.

2.3 - Line 523/524: Cells were supplemented with 7.5 ug/mL trypsin

We thank the reviewer for catching this error. The word "trypsinization" has been corrected to "trypsin" in the revised manuscript (line 621).

2.4 - Line 526/533: 'In the late stage of replication' - what does this refer to?

We thank the reviewer for pointing out this ambiguous phrasing. In the revised manuscript, we have replaced "In the late stage of replication" with the specific time points (12 hpi for PEDV and 10 hpi for PDCoV) in both the RIC-seq and RNA-seq library preparation sections.

2.5 - Line 529: Provide units as RCF not as RPM.

We have changed the centrifugation speed from "3,000 rpm" to "1,000 × g" in the RIC-seq library preparation section (line 626).

2.6 - Lines 555 - 588: it is currently unclear as to what the authors have done versus what the recommended pipeline is e.g. two different alignment programs are listed. Consider separating the processing of RNA-RNA interactome and RNA-seq data into different sections.

We thank the reviewer for this helpful suggestion. In the revised manuscript, these two workflows have been separated into distinct subsections:

1) Processing of RNA-RNA interactome data. This subsection describes the pipeline for identifying chimeric reads that represent genuine RNA-RNA contacts, including the use of STAR and BWA for alignment, classification of intra- vs. inter-molecular interactions and removal of sgRNA junction-derived chimeric reads (using coordinates from matched RNA-seq samples) to avoid contamination from transcription products.
2) Processing of RNA-seq data. This subsection describes the identification of sgRNA junctions from RNA-seq reads, using the same alignment tools but with the purpose of detecting discontinuous transcription events. The resulting junction lists are used both for quantifying sgRNA abundance and for filtering the RNA-RNA interactome data.

The revised sections now clearly distinguish between the two analyses and avoid the appearance of a single, ambiguous pipeline. The specific changes are located at lines 651 to 693 in the revised Methods.

2.7 - Line 561: 'We refer to adopt the pipeline' - poor phrasing.
We thank the reviewer for catching this error. The poorly phrased sentence has been removed from the revised Methods section.

2.8 - Line 562: '...divided into the following 4 steps' - there are 5 steps listed.
We thank the reviewer for catching this error. The step count has been corrected from 4 to 5 in the revised Methods section.

2.9 - Line 637: Please confirm that the order nucleotides on the x- and y-axis.
We thank the reviewer for this clarification. The matrix shown in equation (1) is a 4×4 scoring matrix used for aligning two RNA sequences. The rows and columns correspond to the four RNA bases (A, U, C, G) in the order A, U, C, G, respectively. Specifically, the row index represents the base in the 5′ sequence, and the column index represents the base in the 3′ sequence. This description has been added in the revised manuscript (lines 738–739) to avoid ambiguity. The matrix itself remains unchanged.

2.10 - Line 666: 'we reverse sequences' - poor phrasing.
As noted in the response to Reviewer 1 (Comment 1.1), parallel RNA-RNA base pairing is rare. The correlation supporting such pairing was re-examined and found to be driven primarily by the N gene sgRNA; after removal of the N gene, the correlation was no longer robust. Based on this re-analysis, all RNA structure predictions that involved parallel strand interactions (original Figure 3C and Figures S6–S9) have been

removed from the manuscript. Consequently, the Methods description that included the phrase “we reverse sequences” has been deleted.

2.11 - Line 669: The constraints placed here need to be explained to an audience not familiar with RNAfold.

As described above (response to 2.10), the original RNA secondary structure predictions that used RNAfold with constraints (intended for parallel-pairing models) have been removed.

For the conventional structures included in the newly added quality control analysis (e.g., the UTRs of PEDV and PDCoV), a different approach based on the RIC-seq method (PMID: 34168138) has been adopted. In this method, the viral genome is segmented into short structural domains. Each domain is folded independently using RNAstructure, with the most significant RNA-RNA interactions detected by RIC-seq applied as folding constraints. Long-range and full-genome structures are then progressively assembled. For regions lacking detectable RNA-RNA interactions, structures are predicted based on minimum free energy. This procedure does not involve RNAfold constraints. The revised Methods (lines 774–781) now describe this workflow, and the previous explanation of RNAfold constraints has been removed.

2.12 - Line 670-673: What were the selected parameters for the screen?

The original screening step was designed to select predicted structures that matched parallel (same-direction) RNA-RNA interactions (TRS-L/TRS-B). As described above, all parallel pairing predictions have been removed from the manuscript, and this screening step has been deleted.

26th Jun 2026

Manuscript Number: MSB-2025-13406R
Title: Cross-Genus Analysis Reveals Architecturally Programmed sgRNA Synthesis Patterns in Coronaviruses

Dear Dr. Wei,

Thank you for the submission of your revised manuscript to Molecular Systems Biology. I am pleased to inform you that we will be able to accept your manuscript pending the following final amendments and appropriate response to reviewers:

1) Please update the Author Checklist with the manuscript id number in the general information table at the top of the checklist.
2) Please note that we now mandate that all corresponding authors list an ORCID digital identifier. Currently an ORCID ID is missing for Dr. Fan Wang. An email with instructions to Dr. Wang was sent on June 4th. We encourage all authors to supply an ORCID identifier, which will be linked to their name for unambiguous name identification.
3) Please format the Data availability section according to the example below.
"The datasets and computer code produced in this study are available in the following databases:
- Chip-Seq data: Gene Expression Omnibus GSE46748 (https://www.ncbi.nlm.nih.gov/geo/query/acc.cgi?acc=GSE46748)
- Modeling computer scripts: GitHub (https://github.com/SysBioChalmers/GECKO/releases/tag/v1.0)
- [data type]: [full name of the resource] [accession number/identifier] ([doi or URL or identifiers.org/DATABASE:ACCESSION])"
4) Our journal encourages inclusion of *data citations in the reference list* to directly cite datasets that were re-used and obtained from public databases. Data citations in the article text are distinct from normal bibliographical citations and should directly link to the database records from which the data can be accessed. In the main text, data citations are formatted as follows: "Data ref: Smith et al, 2001" or "Data ref: NCBI Sequence Read Archive PRJNA342805, 2017". In the Reference list, data citations must be labeled with "[DATASET]". A data reference must provide the database name, accession number/identifiers and a resolvable link to the landing page from which the data can be accessed at the end of the reference. Further instructions are available at: https://link.springer.com/journal/44320/submission-guidelines#cms-Reference-guidelines
5) Please rename "Competing Interests" to "Disclosure and competing interests statement". In addition, please be aware that employment in a biotech company should be disclosed in the statement. We updated our journal's competing interests policy in January 2022 and request authors to consider both actual and perceived competing interests. Please review the policy https://link.springer.com/partners/embo-press/editorial-policies#Competing%20interest%20disclosures and update your competing interests if necessary.
6) Please remove the author contributions from the manuscript and specify author contributions in our submission system. CRediT has replaced the traditional author contributions section because it offers a systematic machine-readable author contributions format that allows for more effective research assessment. You are encouraged to use the free text boxes beneath each contributing author's name to add specific details on the author's contribution. More information is available in our guide to authors: https://link.springer.com/journal/44320/submission-guidelines#cms-Manuscript-organisation-and-formatting
7) Please correct the reference citation in the reference list to be alphabetical (not numerical). Where there are more than 10 authors on a paper, only the first 10 should be listed, followed by "et al.". DOIs should only be used for preprints and datasets that have not been published yet. Please check "Author Guidelines" for more information: https://link.springer.com/journal/44320/submission-guidelines#cms-Reference-guidelines
8) In the Methods, please take care of the following:
- Please be sure to include a sentence in the Methods as to whether or not the cell lines were recently authenticated and tested for mycoplasma contamination. Please also be sure to update the Author Checklist with this information and where it can be found in the manuscript.
- Please ensure that a statement on whether or not blinding was done is included in the Methods even if no blinding was done. Please also be sure to update the Author Checklist with this information and where it can be found in the manuscript.

9) Please remove the Reagents and Tools Table from the main manuscript as you have already it uploaded it as a separate file.
10) Please place individual sections of the manuscript in the following order: Title page - Abstract & Keywords - Introduction - Results - Discussion - Methods - Data Availability - Acknowledgements - Disclosure and Competing Interests Statement - References - Figure Legends - (Main Tables with legends if applicable) - Expanded View Figure Legends.
11) For the figures and figure legends, please take care of the following:
- Please remove all figures from main manuscript file and leave only main figure legends placed after the references.
- Please make sure to update the callouts of all figures in the main manuscript text. All callouts should be listed sequentially. Currently figure callouts are missing for Dataset EV1-EV5. In addition, there is no panel D in Figure 4, but it is cited and mentioned in the figure legend.
- EV Dataset legends should be uploaded as a separate tab/sheet in each Excel file.
- Please note that the sub-figure 4D is not labeled in the figure panel in the manuscript. This needs to be rectified.
- Please note that the exact p values are not provided in the legends of 6E.
- Please note that information related to n is missing in the legends 2E.
12) Please upload the Appendix as a single PDF (no separate image files are needed).

13) Please note that funding information should be given in the "Acknowledgements" section. Please ensure that all funding sources are entered into the manuscript submission system and also stated in the manuscript.
14) Please remove the synopsis image from the synopsis text word document and upload it as a separate high-resolution jpeg or png file. The dimensions must be 550 pixels wide x 300 pixels high.
15) As part of the EMBO Publications transparent editorial process initiative (see our policy here: https://www.embopress.org/transparent-process#Review_Process), Molecular Systems Biology will publish online a Peer Review File (PRF) to accompany accepted manuscripts. This file will be published in conjunction with your paper and will include the anonymous referee reports, your point-by-point response and all pertinent correspondence relating to the manuscript. Let us know whether you agree with the publication of the PRF and as here, if you want to remove or not any figures from it prior to publication. Please note that the Authors checklist will be published at the end of the PRF.
16) After your paper is published, we may promote it on social media. If you have any handles or hashtags for Bluesky you would like included, please let us know.
17) Please provide a point-by-point letter INCLUDING my comments as well as the reviewer's reports and your detailed responses (as Word file).

I look forward to reading a new revised version of your manuscript as soon as possible.

Yours sincerely,

Poonam Bheda, PhD
Scientific Editor
Molecular Systems Biology

Reviewer #1:

The revised manuscript is clear and well supported, and the authors have addressed all previous comments. The current claims are in line with the data, and the explanations of experiments and analyses have greatly improved.

Two minor comments remain:

1. Data analysis methods are still ambiguous, specifically, lines 664-667. Please specify exactly which aligner was used and how for each dataset. If both STAR and BWA were used, please explain how the outputs were combined.
2. Primers used for qPCR detection of the PDCoV N RNA are missing from the reagents table.

Reviewer #2:

The authors have satisfactorily addressed all of my original comments.

Dear Dr. Bheda,

Thank you for your positive decision on our manuscript and for providing the final formatting requirements. We have carefully addressed all the editorial comments and the remaining minor points raised by the reviewers. A detailed point-by-point response to all comments is provided below.

We confirm that we agree with the publication of the Peer Review File and have no figures to remove from it.

We hope that the revised manuscript now meets all the requirements for publication in Molecular Systems Biology.

Yours sincerely,

Dengguo Wei

National Key Laboratory of Agricultural Microbiology, College of Veterinary Medicine, Huazhong Agricultural University

No.1, Shizishan Street, Hongshan District, Wuhan, Hubei Province 430070, P.R.China

Tel.: +86 27 87282608

Fax: +86 27 87282608

Editorial Comments

1) Please update the Author Checklist with the manuscript id number in the general information table at the top of the checklist.

We have updated the Author Checklist with the manuscript ID (MSB-2025-13406) in the general information table at the top of the checklist.

2) Please note that we now mandate that all corresponding authors list an ORCID digital identifier. Currently an ORCID ID is missing for Dr. Fan Wang. An email with instructions to Dr. Wang was sent on June 4th. We encourage all authors to supply an ORCID identifier, which will be linked to their name for unambiguous name identification.

We have communicated with Dr. Fan Wang, and his ORCID ID (https://orcid.org/0000-0003-2664-9214) has now been linked to his account and associated with the manuscript in the submission system.

3) Please format the Data availability section according to the example below. "The datasets and computer code produced in this study are available in the following databases:

- Chip-Seq data: Gene Expression Omnibus GSE46748 (https://www.ncbi.nlm.nih.gov/geo/query/acc.cgi?acc=GSE46748)
- Modeling computer scripts: GitHub (https://github.com/SysBioChalmers/GECKO/releases/tag/v1.0)
- [data type]: [full name of the resource] [accession number/identifier] ([doi or URL or identifiers.org/DATABASE:ACCESSION])"

The Data Availability section has been reformatted according to the journal's example. It now reads:

The datasets and computer code produced in this study are available in the following databases:

- RIC-seq and RNA-seq data for PEDV and PDCoV: National Genomics Data Center BioProject PRJCA026550 (https://ngdc.cncb.ac.cn/bioproject/browse/PRJCA026550)
- Analysis scripts: GitHub (https://github.com/WenZi0809/RNA-architecture-underlies-discontinuous-transcription-and-evolution-of-coronavirus)

4) Our journal encourages inclusion of data citations in the reference list to directly cite datasets that were re-used and obtained from public databases. Data citations in the article text are distinct from normal bibliographical citations and should directly link to the database records from which the data can be accessed. In the main text, data citations are formatted as follows: "Data ref: Smith et al, 2001" or "Data ref: NCBI Sequence Read Archive PRJNA342805, 2017". In the Reference list, data citations must be labeled with "[DATASET]". A data reference must provide the database name, accession number/identifiers and a resolvable link to the landing page from which the data can be accessed at the end of the reference.

We have added data citations for the public datasets re-analyzed in this study. In the main text, these are cited as "Data ref: Mao et al, 2022" for the PEDV time-course

dataset (PRJNA801927), with corresponding entries added to the Reference list with the "[DATASET]" tag, including the database name, accession number, and resolvable link to the data.

5) Please rename "Competing Interests" to "Disclosure and competing interests statement". In addition, please be aware that employment in a biotech company should be disclosed in the statement. We updated our journal's competing interests policy in January 2022 and request authors to consider both actual and perceived competing interests. Please review the policy https://link.springer.com/partners/embo-press/editorial-policies#Competing%20interest%20disclosures and update your competing interests if necessary.
The section heading has been renamed to "Disclosure and competing interests statement." The statement has been reviewed and updated according to the journal's policy. The authors declare no competing interests.

6) Please remove the author contributions from the manuscript and specify author contributions in our submission system. CRediT has replaced the traditional author contributions section because it offers a systematic machine-readable author contributions format that allows for more effective research assessment. You are encouraged to use the free text boxes beneath each contributing author's name to add specific details on the author's contribution.
The Author contributions section has been removed from the manuscript. Author contributions have been specified in the submission system using the CRediT format.

7) Please correct the reference citation in the reference list to be alphabetical (not numerical). Where there are more than 10 authors on a paper, only the first 10 should be listed, followed by "et al.". DOIs should only be used for preprints and datasets that have not been published yet.
The reference list has been reformatted to alphabetical order. All entries with more than 10 authors have been truncated to the first 10 authors followed by "et al."

8) In the Methods, please take care of the following:
- Please be sure to include a sentence in the Methods as to whether or not the cell lines were recently authenticated and tested for mycoplasma contamination. Please also be sure to update the Author Checklist with this information and where it can be found in the manuscript.
- Please ensure that a statement on whether or not blinding was done is included in the Methods even if no blinding was done. Please also be sure to update the Author Checklist with this information and where it can be found in the manuscript.
Both required statements have been added to the Methods section:

- A statement on cell line authentication and mycoplasma testing has been included in the "Cell culture and virus infection" subsection: "Vero E6 and LLC-PK1 cells were not authenticated by STR profiling. Both cell lines tested negative for mycoplasma contamination."

- A statement on blinding has been added to the "Overexpression of ORF_{UTR3} and qRT-PCR" subsection: "No blinding was applied during sample processing or data acquisition."

The Author Checklist has been updated accordingly, with references to the corresponding sections in the manuscript.

9) Please remove the Reagents and Tools Table from the main manuscript as you have already uploaded it as a separate file.

The Reagents and Tools Table has been removed from the main manuscript file. It remains available as a separate uploaded file.

10) Please place individual sections of the manuscript in the following order: Title page - Abstract & Keywords - Introduction - Results - Discussion - Methods - Data Availability - Acknowledgements - Disclosure and Competing Interests Statement - References - Figure Legends - (Main Tables with legends if applicable) - Expanded View Figure Legends.

The manuscript sections have been reordered according to the journal's requirements. The current order is: Title page, Abstract & Keywords, Introduction, Results, Discussion, Methods, Data Availability, Acknowledgements, Disclosure and Competing Interests Statement, References and Figure Legends.

11) For the figures and figure legends, please take care of the following:
- Please remove all figures from main manuscript file and leave only main figure legends placed after the references.
- Please make sure to update the callouts of all figures in the main manuscript text. All callouts should be listed sequentially. Currently figure callouts are missing for Dataset EV1-EV5. In addition, there is no panel D in Figure 4, but it is cited and mentioned in the figure legend.
- EV Dataset legends should be uploaded as a separate tab/sheet in each Excel file.
- Please note that the sub-figure 4D is not labeled in the figure panel in the manuscript. This needs to be rectified.
- Please note that the exact p values are not provided in the legends of 6E.
- Please note that information related to n is missing in the legends 2E.

All changes are reflected in the revised manuscript and figure files：

- All figures have been removed from the main manuscript file; only figure legends remain, placed after the references.
- Callouts for Dataset EV1-EV5 have been added to the main text where each dataset is first mentioned, and the callout sequence has been verified to be sequential. In addition, to streamline the supplementary data, two datasets previously containing redundant information on genomic deletions (EV3 and EV5) have been merged into a single dataset (EV2), and the remaining datasets have been renumbered according to their order of appearance in the text. The EV Dataset legends have been added as separate tabs/sheets in each corresponding Excel file.

- Sub-figure 4D has been labeled in the figure panel.
- Exact p-values have been added to the legend of Figure 6E.
- The sample size information (n) has been added to the legend of Figure 2E.

12) Please upload the Appendix as a single PDF (no separate image files are needed).
All Appendix figures have been consolidated into a single PDF file as requested.

13) Please note that funding information should be given in the "Acknowledgements" section. Please ensure that all funding sources are entered into the manuscript submission system and also stated in the manuscript.
All funding sources have been listed in the Acknowledgements section and have also been entered into the manuscript submission system.

14) Please remove the synopsis image from the synopsis text word document and upload it as a separate high-resolution jpeg or png file. The dimensions must be 550 pixels wide x 300 pixels high.
The synopsis image has been removed from the synopsis text document and uploaded as a separate high-resolution PNG file (550 × 300 pixels).

15) As part of the EMBO Publications transparent editorial process initiative, Molecular Systems Biology will publish online a Peer Review File (PRF) to accompany accepted manuscripts. This file will be published in conjunction with your paper and will include the anonymous referee reports, your point-by-point response and all pertinent correspondence relating to the manuscript. Let us know whether you agree with the publication of the PRF and as here, if you want to remove or not any figures from it prior to publication. Please note that the Authors checklist will be published at the end of the PRF.
We agree with the publication of the Peer Review File and have no figures to remove from it.

16) After your paper is published, we may promote it on social media. If you have any handles or hashtags for Bluesky you would like included, please let us know.
Please feel free to promote the paper. We do not have specific Bluesky handles to suggest at this time.

17) Please provide a point-by-point letter INCLUDING my comments as well as the reviewer's reports and your detailed responses (as Word file).
This document serves as the complete point-by-point letter, including all editorial comments, reviewer reports, and detailed responses.

Reviewer #1
The revised manuscript is clear and well supported, and the authors have addressed all previous comments. The current claims are in line with the data, and the explanations of experiments and analyses have greatly improved.

Two minor comments remain:

1. Data analysis methods are still ambiguous, specifically, lines 664-667. Please specify exactly which aligner was used and how for each dataset. If both STAR and BWA were used, please explain how the outputs were combined.

We thank the reviewer for this careful reading. The Methods section (lines 560–564) has been revised to clarify the alignment strategy and how outputs from STAR and BWA were combined. The revised text now reads:

1. Fastp (Chen et al, 2018) is used for quality control, and reads are then aligned to the reference genome using STAR for both standard and chimeric alignments, while unaligned reads are re-aligned with BWA to maximize chimeric fragment recovery;

2. The alignment outputs from STAR and BWA are merged, and paired reads are identified from the combined output;

The sequential use of the two aligners and the integration of their outputs is now clearly explained.

2. Primers used for qPCR detection of the PDCoV N RNA are missing from the reagents table.

We thank the reviewer for pointing out this omission. The primers used for qPCR detection of the PDCoV N gene (forward: 5′-TTCGTGGCAATGGAGTTC-3′; reverse: 5′-GGATGGAGGAATCGGAGTA-3′) have now been added to the Reagents and Tools Table.

Reviewer #2

The authors have satisfactorily addressed all of my original comments.

We thank the reviewer for their positive assessment and are glad that all concerns have been addressed to their satisfaction.

14th Jul 2026
Manuscript number: MSB-2025-13406RR
Title: Cross-Genus Analysis Reveals Architecturally Programmed sgRNA Synthesis Patterns in Coronaviruses

Dear Dr. Wei,

Thank you again for sending us your revised manuscript. We are now satisfied with the modifications made and I am pleased to inform you that your paper has been accepted for publication.

Your manuscript will be processed for publication by EMBO Press. It will be copy edited and you will receive page proofs prior to publication. Please note that you will be contacted by Springer Nature Author Services to complete licensing and payment information.

You may qualify for financial assistance for your publication charges - either via a Springer Nature fully open access agreement or an EMBO initiative. Check your eligibility: https://link.springer.com/journal/44320/how-to-publish-with-us

Should you be planning a Press Release on your article, please notify the production team via this form in order to coordinate publication and release dates: https://forms.cloud.microsoft/e/eH4A8JVP6g

If you have any questions, please do not hesitate to contact the Editorial Office. Thank you for your contribution to Molecular Systems Biology.

Yours sincerely,

Sincerely,

Poonam Bheda, PhD
Scientific Editor
Molecular Systems Biology

>>> Please note that it is Molecular Systems Biology policy for the transcript of the editorial process (containing referee reports and your response letter) to be published as an online supplement to each paper. If you do NOT want this, you will need to inform the Editorial Office via email immediately. More information is available here: https://link.springer.com/partners/embo-press/editorial-policies#Peer%20review

EMBO Press Author Checklist

Corresponding Author Name: Dengguo Wei
Journal Submitted to: Molecular Systems Biology
Manuscript Number: MSB-2025-13406

USEFUL LINKS FOR COMPLETING THIS FORM
The EMBO Journal - Author Guidelines
EMBO Reports - Author Guidelines
Molecular Systems Biology - Author Guidelines
EMBO Molecular Medicine - Author Guidelines

Reporting Checklist for Life Science Articles (updated January
This checklist is adapted from Materials Design Analysis Reporting (MDAR) Checklist for Authors. MDAR establishes a minimum set of requirements in transparent reporting in the life sciences (see Statement of Task: 10.31222/osf.io/9sm4x). Please follow the journal's guidelines in preparing your
Please note that a copy of this checklist will be published alongside your article.

Abridged guidelines for figures
1. Data
The data shown in figures should satisfy the following conditions:
- the data were obtained and processed according to the field's best practice and are presented to reflect the results of the experiments in an accurate and unbiased manner.
- ideally, figure panels should include only measurements that are directly comparable to each other and obtained with the same assay.
- plots include clearly labeled error bars for independent experiments and sample sizes. Unless justified, error bars should not be shown for technical
- if n<5, the individual data points from each experiment should be plotted. Any statistical test employed should be justified.
- Source Data should be included to report the data underlying figures according to the guidelines set out in the authorship guidelines on Data

2. Captions
Each figure caption should contain the following information, for each panel where they are relevant:
- a specification of the experimental system investigated (eg cell line, species name).
- the assay(s) and method(s) used to carry out the reported observations and measurements.
- an explicit mention of the biological and chemical entity(ies) that are being measured.
- an explicit mention of the biological and chemical entity(ies) that are altered/varied/perturbed in a controlled manner.
- the exact sample size (n) for each experimental group/condition, given as a number, not a range;
- a description of the sample collection allowing the reader to understand whether the samples represent technical or biological replicates (including how many animals, litters, cultures, etc.).
- a statement of how many times the experiment shown was independently replicated in the laboratory.
- definitions of statistical methods and measures:
  - common tests, such as t-test (please specify whether paired vs. unpaired), simple χ2 tests, Wilcoxon and Mann-Whitney tests, can be unambiguously identified by name only, but more complex techniques should be described in the methods section;
  - are tests one-sided or two-sided?
  - are there adjustments for multiple comparisons?
  - exact statistical test results, e.g., P values = x but not P values < x;
  - definition of 'center values' as median or average;
  - definition of error bars as s.d. or s.e.m.

Please complete ALL of the questions below.
Select "Not Applicable" only when the requested information is not relevant for your study.

Materials

Newly Created Materials	Information included in the manuscript?	In which section is the information available? (Reagents and Tools Table, Materials and Methods, Figures, Data Availability Section)
New materials and reagents need to be available; do any restrictions apply?	Not Applicable	

Antibodies	Information included in the manuscript?	In which section is the information available? (Reagents and Tools Table, Materials and Methods, Figures, Data Availability Section)
For **antibodies** provide the following information:<br>- Commercial antibodies: RRID (if possible) or supplier name, catalogue number and or/clone number<br>- Non-commercial: RRID or citation	Not Applicable	

DNA and RNA sequences	Information included in the manuscript?	In which section is the information available? (Reagents and Tools Table, Materials and Methods, Figures, Data Availability Section)
**Short novel DNA or RNA including primers, probes**: provide the sequences.	Yes	Materials and Methods

Cell materials	Information included in the manuscript?	In which section is the information available? (Reagents and Tools Table, Materials and Methods, Figures, Data Availability Section)
**Cell lines:** Provide species information, strain. Provide accession number in repository **OR** supplier name, catalog number, clone number, and/**OR** RRID.	Yes	Materials and Methods
**Primary cultures:** Provide species, strain, sex of origin, genetic modification status.	Not Applicable	
Report if the cell lines were recently **authenticated** (e.g., by STR profiling) and tested for mycoplasma contamination.	Yes	Materials and Methods

Experimental animals	Information included in the manuscript?	In which section is the information available? (Reagents and Tools Table, Materials and Methods, Figures, Data Availability Section)
**Laboratory animals or Model organisms:** Provide species, strain, sex, age, genetic modification status. Provide accession number in repository **OR** supplier name, catalog number, clone number, **OR** RRID.	Not Applicable	
**Animal observed in or captured from the field:** Provide species, sex, and age where possible**.**	Not Applicable	
Please detail **housing and husbandry conditions**.	Not Applicable	

Plants and microbes	Information included in the manuscript?	In which section is the information available? (Reagents and Tools Table, Materials and Methods, Figures, Data Availability Section)
**Plants:** provide species and strain, ecotype and cultivar where relevant, unique accession number if available, and source (including location for collected wild specimens).	Not Applicable	
**Microbes:** provide species and strain, unique accession number if available, and source.	Yes	Materials and Methods

Human research participants	Information included in the manuscript?	In which section is the information available? (Reagents and Tools Table, Materials and Methods, Figures, Data Availability Section)
If collected and within the bounds of privacy constraints report on age, sex and gender or ethnicity for all study participants.	Not Applicable	

Core facilities	Information included in the manuscript?	In which section is the information available? (Reagents and Tools Table, Materials and Methods, Figures, Data Availability Section)
If your work benefited from core facilities, was their service mentioned in the acknowledgments section?	Not Applicable	

Design

Study protocol	Information included in the manuscript?	In which section is the information available? (Reagents and Tools Table, Materials and Methods, Figures, Data Availability Section)
If study protocol has been **pre-registered, provide DOI in the manuscript**. For clinical trials, provide the trial registration number **OR** cite DOI.	Not Applicable	
Report the **clinical trial registration number** (at ClinicalTrials.gov or equivalent), where applicable.	Not Applicable	

Laboratory protocol	Information included in the manuscript?	In which section is the information available? (Reagents and Tools Table, Materials and Methods, Figures, Data Availability Section)
Provide DOI OR other citation details if **external detailed step-by-step protocols** are available.	Not Applicable	

Experimental study design and statistics	Information included in the manuscript?	In which section is the information available? (Reagents and Tools Table, Materials and Methods, Figures, Data Availability Section)
Include a statement about **sample size** estimate even if no statistical methods were used.	Not Applicable	
Were any steps taken to minimize the effects of subjective bias when allocating animals/samples to treatment (e.g. **randomization procedure**)? If yes, have they been described?	Not Applicable	
Include a statement about **blinding** even if no blinding was done.	Yes	Materials and Methods
Describe **inclusion/exclusion criteria** if samples or animals were excluded from the analysis. Were the criteria pre-established?<br><br>If sample or data points were omitted from analysis, report if this was due to attrition or intentional exclusion and provide justification.	Yes	Materials and Methods
For every figure, are **statistical tests** justified as appropriate? Do the data meet the assumptions of the tests (e.g., normal distribution)? Describe any methods used to assess it. Is there an estimate of variation within each group of data? Is the variance similar between the groups that are being statistically compared?	Yes	Materials and Methods; Figures

Sample definition and in-laboratory replication	Information included in the manuscript?	In which section is the information available? (Reagents and Tools Table, Materials and Methods, Figures, Data Availability Section)
In the figure legends: state number of times the experiment was **replicated** in laboratory.	Yes	Figures
In the figure legends: define whether data describe **technical or biological replicates**.	Yes	Figures

Ethics

Ethics	Information included in the manuscript?	In which section is the information available? (Reagents and Tools Table, Materials and Methods, Figures, Data Availability Section)
Studies involving **human participants**: State details of **authority granting ethics approval** (IRB or equivalent committee(s), provide reference number for approval.	Not Applicable	
Studies involving **human participants**: Include a statement confirming that **informed consent** was obtained from all subjects and that the experiments conformed to the principles set out in the WMA Declaration of Helsinki and the Department of Health and Human Services Belmont Report.	Not Applicable	
Studies involving **human participants:** For publication of **patient photos**, include a statement confirming that consent to publish was obtained.	Not Applicable	
Studies involving experimental **animals**: State details of **authority granting ethics approval** (IRB or equivalent committee(s), provide reference number for approval. Include a statement of compliance with ethical regulations.	Not Applicable	
Studies involving **specimen and field samples:** State if relevant **permits** obtained, provide details of authority approving study; if none were required, explain why.	Not Applicable	

Dual Use Research of Concern (DURC)	Information included in the manuscript?	In which section is the information available? (Reagents and Tools Table, Materials and Methods, Figures, Data Availability Section)
Could your study fall under dual use research restrictions? Please check biosecurity documents and list of **select agents and toxins** (CDC): https://www.selectagents.gov/sat/list.htm	Not Applicable	
If you used a select agent, is the security level of the lab appropriate and reported in the manuscript?	Not Applicable	
If a study is subject to dual use research of concern regulations, is the name of the **authority granting approval and reference number** for the regulatory approval provided in the manuscript?	Not Applicable	

Reporting

The MDAR framework recommends adoption of discipline-specific guidelines, established and endorsed through community initiatives. Journals have their own policy about requiring specific guidelines and recommendations to complement MDAR.

Adherence to community standards	Information included in the manuscript?	In which section is the information available? (Reagents and Tools Table, Materials and Methods, Figures, Data Availability Section)
State if relevant guidelines or checklists (e.g., **ICMJE, MIBBI, ARRIVE, PRISMA**) have been followed or provided.	Not Applicable	
For **tumor marker prognostic studies,** we recommend that you follow the **REMARK** reporting guidelines (see link list at top right). See author guidelines, under 'Reporting Guidelines'. Please confirm you have followed these guidelines.	Not Applicable	
For **phase II and III randomized controlled trials**, please refer to the **CONSORT** flow diagram (see link list at top right) and submit the CONSORT checklist (see link list at top right) with your submission. See author guidelines, under 'Reporting Guidelines'. Please confirm you have submitted this list.	Not Applicable	

Data Availability

Data availability	Information included in the manuscript?	In which section is the information available? (Reagents and Tools Table, Materials and Methods, Figures, Data Availability Section)
Have **primary datasets** been deposited according to the journal's guidelines (see 'Data Deposition' section) and the respective accession numbers provided in the Data Availability Section?	Yes	Data Availability Section
Were **human clinical and genomic datasets** deposited in a public access-controlled repository in accordance to ethical obligations to the patients and to the applicable consent agreement?	Not Applicable	
Are **computational models** that are central and integral to a study available without restrictions in a machine-readable form? Were the relevant accession numbers or links provided?	Not Applicable	
If publicly available data were reused, provide the respective **data citations in the reference list**.	Yes	Data Availability Section
